# Age-Specific Quantification of Overweight/Obesity Risk Factors From Infancy to Adolescence and Differences by Educational Level of Parents

**DOI:** 10.3389/ijph.2023.1605798

**Published:** 2023-11-15

**Authors:** Claudia Börnhorst, Wolfgang Ahrens, Stefaan De Henauw, Monica Hunsberger, Denéz Molnár, Luis A. Moreno, Paola Russo, Anton Schreuder, Elida Sina, Michael Tornaritis, Stefanie Vandevijvere, Thomas Veidebaum, Tanja Vrijkotte, Kathleen Wijnant, Maike Wolters

**Affiliations:** ^1^ Leibniz Institute for Prevention Research and Epidemiology (BIPS), Bremen, Germany; ^2^ Institute of Statistics, Faculty of Mathematics and Computer Science, University of Bremen, Bremen, Germany; ^3^ Department of Public Health and Primary Care, Ghent University, Ghent, Belgium; ^4^ School of Public Health and Community Medicine, Institute of Medicine, Sahlgrenska Academy, University of Gothenburg, Gothenburg, Sweden; ^5^ Department of Pediatrics, Medical School, University of Pécs, Pécs, Hungary; ^6^ GENUD (Growth, Exercise, Nutrition and Development) Research Group, Faculty of Health Sciences, Instituto Agroalimentario de Aragón (IA2), Instituto de Investigación Sanitaria Aragón (IIS Aragón), University of Zaragoza, Zaragoza, Spain; ^7^ Centro de Investigación Biomédica en Red de Fisiopatología de la Obesidad y Nutrición (CIBERObn), Madrid, Spain; ^8^ Institute of Food Sciences, National Research Council, Avellino, Italy; ^9^ Amsterdam University Medical Center, Amsterdam, Netherlands; ^10^ Department of Social Medicine Public and Occupational Health, Amsterdam Public Health Research Institute, Amsterdam UMC, University of Amsterdam, Amsterdam, Netherlands; ^11^ Research and Educational Institute of Child Health, Strovolos, Cyprus; ^12^ Sciensano, Directorate Epidemiology and Public Health, Brussels, Belgium; ^13^ Estonian Centre of Behavioral and Health Sciences, National Institute for Health Development, Tallinn, Estonia

**Keywords:** body mass index, childhood overweight/obesity, IDEFICS/I.Family cohort, overweight/obesity risk factors, parental education

## Abstract

**Objectives:** To explore the age-dependent associations between 26 risk factors and BMI in early life, and differences by parental educational level.

**Methods:** Data of 10,310 children (24,155 measurements) aged 2–16 years participating in a multi-centre European cohort from 2007 to 2014 were utilized. Trajectories of overweight/obesity risk factors and their age-specific associations with BMI were estimated using polynomial mixed-effects models.

**Results:** Exposure to most unfavourable factors was higher in the low/medium compared to the high education group, e.g., for PC/TV time (12.6 vs. 10.6 h/week). Trajectories of various risk factors markedly changed at an age of 9–11 years. Having a family history of obesity, maternal BMI, pregnancy weight gain and birth weight were positively associated with BMI trajectories throughout childhood/adolescence in both education groups; associations of behavioural factors with BMI were small. Parental unemployment and migrant background were positively associated with BMI in the low/medium education group.

**Conclusion:** Associations of risk factors with BMI trajectories did not essentially differ by parental education except for social vulnerabilities. The age period of 9–11 years may be a sensitive period for adopting unfavourable behaviours.

## Introduction

Overweight/obesity (OW/OB) has reached alarming proportions among children and adolescents, both in high and in low/middle income countries [1]. Childhood OW/OB is linked to adulthood OW/OB, and in turn to a higher risk of chronic diseases including certain types of cancer [2], cardio-metabolic diseases [3] as well as increased mortality and premature death [4]. Several factors have been associated with childhood OW/OB. These can be divided into non-modifiable factors (from a child’s perspective) such as socio-economic status [5], genetic predisposition [6], family structure [7] and pre-, post- and perinatal factors [8, 9] versus modifiable behavioural factors such as sleep [10], diet, sedentary time and physical activity [11]. The persistently high prevalence of OW/OB in children and adolescents in Western societies [1] underlines the pivotal role of risk factors to which the child is not irrevocably exposed to, i.e., modifiable factors.

Recent studies reaffirmed that particularly socio-economically disadvantaged groups such as children of low-educated or unemployed parents or from low-income families are at increased risk of obesity and subsequent metabolic disturbances in Western countries [12–14]. Moreover, unfavourable health-related behaviours were shown to be more prevalent in children of lower educated parents [15]. To date, health interventions have attained only small effects on reducing the risk of OW/OB [16, 17], and particularly vulnerable groups have only been reached to a limited degree [18, 19].

Research into the time windows when OW/OB-related behaviours are shaped, e.g., critical developmental periods such as school entry or start of puberty, is still lacking. Furthermore, periods when non-modifiable factors exert the strongest effects on weight status are hardly known. In this context, impediments to a favourable behaviour change and the role of social vulnerabilities seem to be of pivotal importance. To close this knowledge gap, this study explores 1) trajectories of a large range of OW/OB-related risk factors as well as 2) associations between OW/OB risk factors and body mass index (BMI) development from childhood to adolescence in children of parents with low/medium vs. high educational level based on data of the pan-European IDEFICS (Identification and Prevention of Dietary- and Lifestyle-Induced Health Effects in Children and Infants)/I.Family cohort [20]. Throughout the analyses, we differentiate between factors being modifiable (e.g., diet and physical activity) vs. non-modifiable factors from a child’s perspective (e.g., early life factors like breast feeding duration or vulnerabilities like migrant background).

The results will help to identify the most important risk factors out of a large number of risk factors acting throughout childhood and adolescence and broaden our understanding on potential time windows during which modifiable and non-modifiable OW/OB risk factors exert their most detrimental effects on OW/OB. To the best of our knowledge, this is the first study exploring a large number of risk factor trajectories and their associations with OW/OB based on a European children cohort considering social vulnerabilities.

## Methods

### Study Population and Data

All analyses are based on the data of the IDEFICS/I. Family cohort, a multi-centre population-based study aiming to investigate the causes of diet- and lifestyle-related diseases in children, adolescents and their families [20, 21]. The baseline survey wave (W0) was conducted in 2007/2008 in eight European countries (Belgium, Cyprus, Estonia, Germany, Hungary, Italy, Spain and Sweden) and included children aged 2.0–9.9 years. In total, 16,229 children fulfilling the inclusion criteria participated. The survey included interviews with parents concerning demographics, family life, health-related behaviours and dietary intakes as well as physical examinations of the children. All measurements were taken using standardized procedures in all eight countries. Additional details on the IDEFICS/I. Family study can be obtained from [20, 21]. A follow-up examination (W1) was conducted in 2009/2010 and the same standardized assessments were applied in 11,043 children who had participated at W0 and in 2,544 newly recruited children. A second follow-up examination (W2) took place in 2013/2014, when 7,118 of the children participating already in W0 or W1 were included. In W2, children aged 12 years or older self-reported their health-related behaviours, wellbeing and family life.

Before children entered the study, parents provided written informed consent. Additionally, all children ≥12 years gave written consent, while younger children gave oral assent in addition to parental consent for the examinations and sample collection. Ethical approval was obtained from the institutional review boards of all eight study centres. The IDEFICS/I. Family cohort is registered under ISRCTN62310987.

### Outcome: BMI

Height (cm) was measured to the nearest 0.1 cm with a calibrated stadiometer (Seca 225 stadiometer, Birmingham, United Kingdom), body weight (kg) was measured in fasting state in light underwear on a calibrated scale accurate to 0.1 kg (adapted Tanita BC 420 MA for children ≤6 years, BC 418 MA for children >6 years, Tanita Europe GmbH, Sindelfingen, Germany). BMI was calculated as weight (kg) divided by height (m) squared. BMI measurements were converted to age- and sex-specific z-scores using the extended criteria of the International Obesity Task Force [22].

### Educational Level of Parents

Highest educational level of parents was categorized according to the International Standard Classification of Education (ISCED) [23]. The maximum ISCED level of both parents was dichotomized (low/medium = ISCED levels 0, 1, 2, 3, 4, 5 vs. high = ISCED levels 6, 7, 8) and used for stratification in the analyses. In case of single parents, the highest educational level of that parent was used.

### Exposures

For our analyses, we selected 13 OW/OB risk factors that are non-modifiable focussing on early life factors and familial/social vulnerabilities: family history of obesity (yes vs. no), maternal BMI (kg/m^2^), smoking during pregnancy (never/rarely vs. several occasions a week/daily), weight gain during pregnancy (kg), pre-term delivery (yes vs. no), birth weight (1 unit∼100 g), total breast feeding (in months), age at introduction of solid foods (month), migrant status (yes vs. no), number of children in household, being an only child (yes vs. no), one-parent family (yes vs. no), parental unemployment (yes vs. no). We further selected 13 modifiable risk factors focussing on health-related behaviours and wellbeing: major frustrations, e.g., at school (yes vs. no/missing), wellbeing score (ranging from 0 to 48; assessed with the KINDL-R [24, 25]), nocturnal sleep duration (h/night), number of media in bedroom, average PC/TV time (h/week), membership in sports club (yes vs. no), active transport (yes vs. no), as well as dietary intakes of water (times/day), fruits (times/day), vegetables (times/day), sweetened drinks (times/day), savoury fast or snack food (times/day) and simple sugar foods (times/day).

Variables were selected based on previous literature as well as availability of data in the IDEFICS/I. Family cohort and comparability across waves. All exposures as well as covariates used for adjustment (e.g., pubertal status and variables reflecting family life and consumer attitudes) are described in detail in Supplementary Material S1. Supplementary Material S2 depicts a directed acyclic graph (DAG) with the assumed associations among risk factors. The DAG was used to identify confounders and corresponding minimal adjustment sets for our exposures.

### Analysis Dataset

In the present analysis, we considered all children that participated in at least two assessment waves of IDEFICS/I.Family. For each child, waves with more than five missing values (out of the considered variables) were excluded from the analysis. The remaining missing values were imputed as detailed in Supplementary Material S3.

**FIGURE 1:**
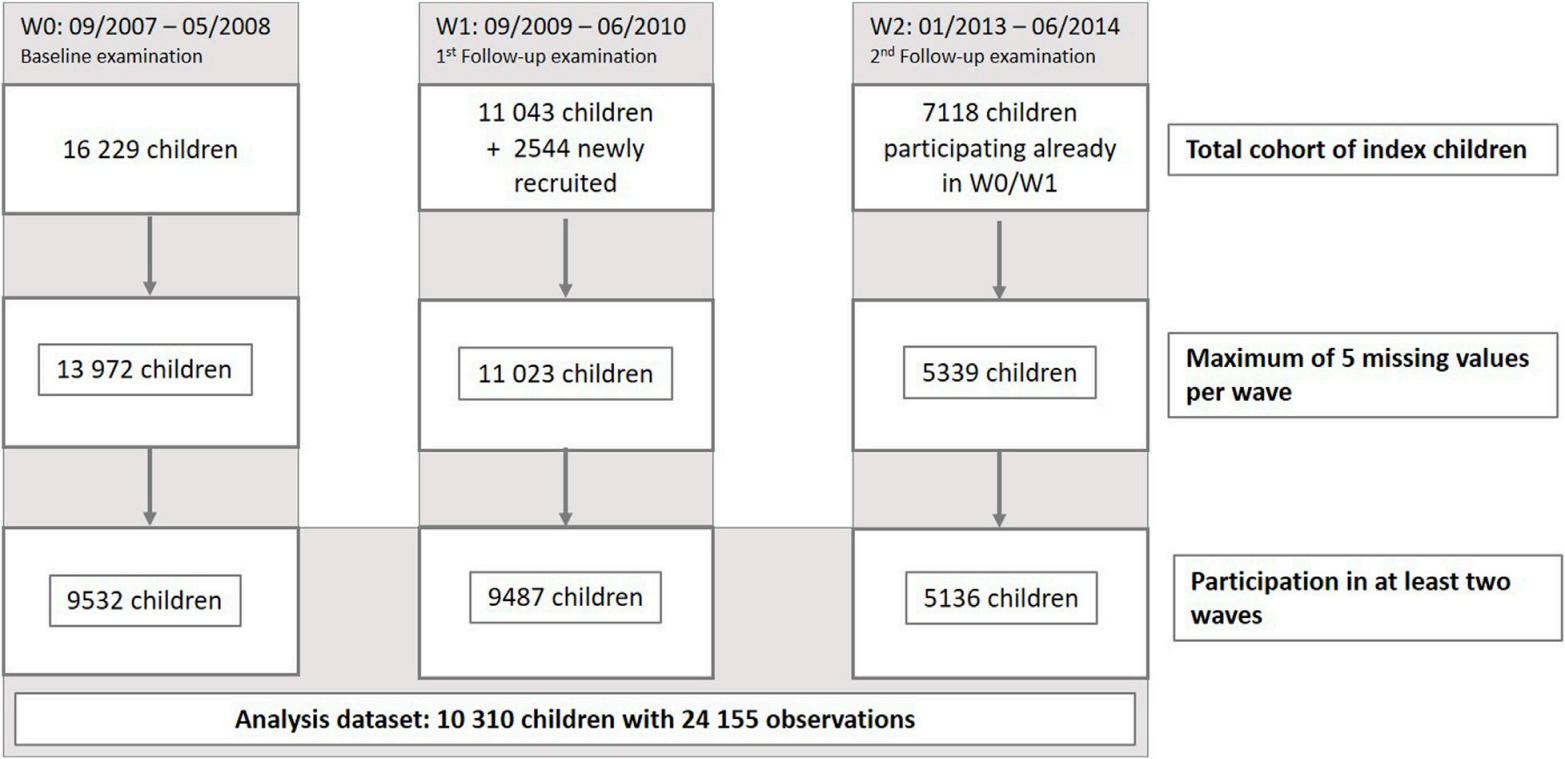
Flow chart depicting the selection process leading to the final study sample (IDEFICS/I.Family study, eight European countries, 2007–2014).

The analysis group consisted of 10,310 children in total, of which 6,775 had participated in two and 3,535 in three examination waves (9,532 at W0, 9,487 at W1 and 5,136 at W3). A flow chart depicting the selection process leading to this analysis group is shown in Figure 1.

### Estimation of Age-Dependent Changes (Trajectories) of OW/OB Risk Factors

Polynomial linear mixed-effects models [26] with a random intercept and random slope and three powers of age were used to assess the development of continuous risk factors from infancy to adolescence; analogously logistic mixed models including a random intercept and three powers of age were used to estimate the evolution of binary risk factors. All risk factor models were adjusted for sex and region of residence (North/Central Europe, Southern Europe, Eastern Europe) and estimated stratified by low/medium vs. high educational level of the parents (classified according to the International Standard Classification of Education; ISCED, 2011 [23]). Least square means (also called marginal means) were calculated as a linear combination (sum) of the estimated effects from our polynomial mixed-effects models (using the LSMEANS statement in SAS Proc MIXED). Least square means were estimated at selected ages to plot the risk factor trajectories and compare the risk factor changes in boys and girls from 3 to 15 years of age. A common limitation of polynomial models is that the effect estimates at the lower and upper end of the covered age range are less stable. For this reason, we only report estimates from 3 to 15 years of age (while our models were estimated based on data covering the age span from 2 to 16 years) and estimates for the ages 3 and 15 should be interpreted with caution.

### Estimation of Age-Dependent Associations Between Risk Factors and BMI

Polynomial linear mixed models with two levels (measurement occasion and individual) and three powers of age were used to assess the age-dependent associations between the modifiable and non-modifiable risk factors and BMI [27]. An unstructured covariance matrix was chosen for modelling the covariance of the random effects. Continuous co-variables were centred before model fitting to obtain meaningful model estimates.

For each exposure of interest, a separate model was fitted including interaction terms of that exposure with age, as well as confounders. All models included sex and geographic region and corresponding interactions with age. Further confounders were selected for each exposure based on the DAG depicted in Supplementary Material S2. Supplementary Material S4 provides a table listing all exposures and corresponding adjustment sets.

Effect estimates from polynomial mixed-effects models are in general difficult to interpret. In order to directly obtain estimates for the associations of the risk factors with BMI at selected ages (3, 6, 9, 12, and 15 years), all models were reparametrized by centring the age variables at ages 3, 6, 9, 12, and 15 years, respectively.

Least square means were calculated to plot and compare BMI trajectories of children with different exposure levels (e.g., of children that sleep 8 vs. 10 h/night) while standardizing over all included confounders.

All analyses were performed stratified by highest educational level of the parents (low/medium vs. high). The group of children with low parental ISCED level was too small to be regarded separately.

Analyses were performed using SAS^®^ statistical software version 9.4 (SAS Institute, Inc., Cary, NC, United States). Proc MI was used for the multiple imputation and Proc MIXED for the linear mixed-effects models. Effect estimates for the multiple imputed datasets were combined using Proc MIANALYZE and 99% confidence intervals (99% CI) were estimated to account at least partially for multiple testing. Effects were considered as statistically significant if the confidence interval does not include the null value.

## Results

Baseline characteristics are displayed in Table 1 stratified by low/medium vs. high educational level of parents. The mean age at baseline was 6.0 years (SD: 1.8 years) and the sexes were almost balanced (50.7% males, 49.3% females). The percentage of children with a migration background (14.6% vs. 11.4%), living with a single parent (17.7% vs. 9.1%) as well as the percentage being an only child (22.6% vs. 16.8%) were higher in the low/medium compared to the high education group. While the mean total breastfeeding duration (6.1 months vs. 7.7 months) and percentage of children being member in a sports club (41.2% vs. 52.1%) was lower in the low/medium education group, the mean number of media in the bedroom (1.1 vs. 0.5 media), average PC/TV time (12.6 vs. 10.6 h/week) and consumption frequency of sweetened drinks (2.1 vs. 1.6 times/day) were higher in the low/medium vs. high education group.

**TABLE 1:** Baseline (W0) characteristics: means and standard deviation of continuous variables and numbers and percentages of categorical variables for the total baseline sample as well as stratified by educational level of parents. (IDEFICS/IFamily study, eight European countries, 2007–2014).

Continuous variables	All (*N* = 9,532)		Low/medium ISCED^a^ (*N* = 4,583)		High ISCED^a^ (*N* = 4,949)	
	Mean	Std	Mean	Std	Mean	Std
Child’s BMI [kg/m^2^]	16.4	2.4	16.8	2.7	16.0	2.0
z-score of BMI by Cole (2012)	0.3	1.2	0.5	1.2	0.1	1.1
Age [years]	6.0	1.8	6.0	1.8	5.9	1.8
Maternal BMI [kg/m^2^]	23.9	4.3	24.6	4.7	23.2	3.8
Pregnancy weight gain [kg]	14.0	5.5	14.1	5.8	13.9	5.2
Child’s birth weight [g]	3356.2	563.2	3323.5	566.4	3386.4	558.7
Introduction of solid foods [month]	5.3	2.2	5.3	2.3	5.2	2.2
Duration of total breast feeding [months]	6.9	6.4	6.1	6.3	7.7	6.5
Number of children in household	2.1	0.8	2.1	0.9	2.1	0.8
Wellbeing score	40.3	4.5	39.8	4.7	40.8	4.3
Nocturnal sleep duration [hours/night]	10.3	1.0	10.2	1.0	10.3	0.9
Number of media in bedroom	0.8	1.2	1.1	1.3	0.5	1.0
PC/TV time [hours/week]	11.6	7.1	12.6	7.7	10.6	6.4
Water [times/day]	3.1	1.5	3.2	1.5	3.0	1.4
Vegetables [times/day]	1.2	0.9	1.1	0.9	1.3	0.9
Fruits [times/day]	1.4	1.1	1.3	1.2	1.4	1.0
Savoury fast and snack food [times/day]	0.5	0.6	0.5	0.7	0.5	0.6
Added sugar food [times/day]	1.2	1.0	1.2	1.2	1.1	0.9
Sweetened drinks [times/day]	1.9	1.7	2.1	1.9	1.6	1.5

Categorical variables	Value	*N*	%	*N*	%	*N*	%
Sex	Female	4,698	49.3	2,238	48.8	2,460	49.7
Migration	Yes	1,232	12.9	667	14.6	565	11.4
Family history of obesity	Yes	2,057	21.6	997	21.8	1,060	21.4
Smoking during pregnancy	Yes	889	9.3	625	13.6	264	5.3
Preterm delivery	Yes	1,053	11	523	11.4	530	10.7
Only child	Yes	1,865	19.6	1,034	22.6	831	16.8
Single parent family	Yes	1,261	13.2	813	17.7	448	9.1
Unemployed for at least 1 year	Yes	190	2.0	138	3.0	52	1.1
Major frustrations	Yes	601	6.3	303	6.6	298	6.0
Membership in a sports club	Yes	4,468	46.9	1,888	41.2	2,580	52.1
Active transport	Yes	3,406	35.7	1,758	38.4	1,648	33.3

Note: The analysis sample consists of 10,310 children out of which 9,532 participated at baseline.

^a^ ISCED, International Standard Classification of Education [23].

### Trajectories of Risk Factors

**FIGURE 2:**
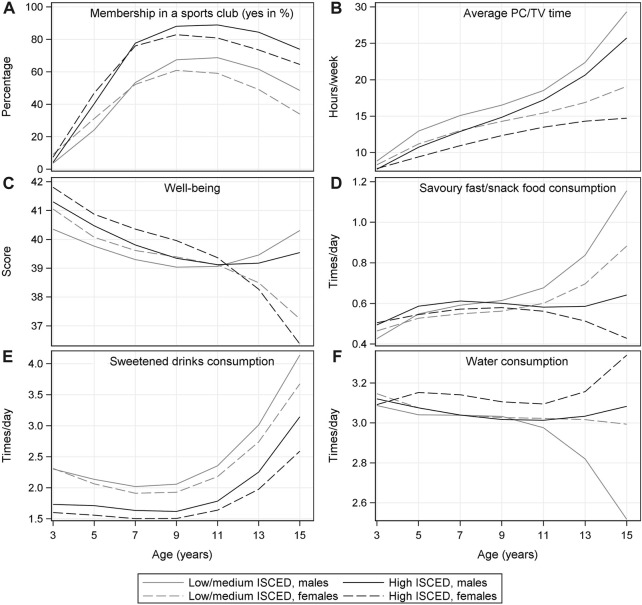
Trajectories of selected risk factors from 3 to 15 years of age by sex and educational level of parents (low/medium vs. high ISCED level); marginal means estimated based on the fractional polynomial mixed effects models (IDEFICS/I.Family study, eight European countries, 2007–2014).

Figure 2 displays the trajectories of selected modifiable risk factors from 3 to 15 years of age stratified by sex and educational level; corresponding numbers and confidence intervals as well as the graphical displays of the trajectories of all remaining time-varying risk factors are shown in Supplementary Material S5. Risk factor trajectories differed by parental educational attainment and sex (see Figure 2 and Supplementary Material S5). From the age of 4 years, sports club membership was higher in boys and among children with high parental educational level. The average PC/TV time continuously increased until adolescence (e.g., from 8.8 to 29.3 h/week in boys in the low/medium education group) and was generally higher among boys and in the low/medium education group. While the consumption of savoury snacks and fast food increased in boys and girls from the low/medium education group after the age of 9, it slightly declined among girls in the high education group. The consumption of sweetened drinks was higher in the low/medium education group and among boys across all ages. However, it increased in all groups after the age of 9 years. Conversely, water consumption decreased in boys with low/medium parental education after the age of 9 years.

The slope of the trajectories changed for many risk factors at the ages of 9–11 years, e.g., the consumption frequency of savoury snacks/fast food and sweetened drinks increased; the mean wellbeing score increased in boys while it decreased in girls. The proportion of children with a sports club membership peaked at 89% at the age of 10–11 years and declined thereafter.

### Age-Specific Associations of Risk Factors With BMI

#### Non-Modifiable Risk Factors

Table 2 depicts the effect estimates and 99% CIs for the associations between the 13 non-modifiable risk factors and BMI at ages 3, 6, 9, 12 and 15 years stratified by parental educational level. With respect to the early life factors, associations with BMI were mainly similar comparing children with low/medium vs. high parental education. For instance, maternal weight gain during pregnancy and birth weight were positively associated with BMI in both education groups from 3 years of age onwards. Also, the family history of obesity and maternal BMI were found to be positively associated with the BMI of the child from age 6 onwards in both education groups with the strengths of the associations increasing with age. For the family history of obesity, effect estimates were larger in the low/medium (increasing from *β* = 0.71 at age 6 to *β* = 1.50 at age 15) compared to the high education group (increasing from *β* = 0.32 at age 6 to *β* = 0.83 at age 15). Smoking during pregnancy revealed a positive association with BMI particularly at age 15 in the low/medium education group (*β* = 1.40 [0.43; 2.37]; 99% CI in brackets).

**TABLE 2:** Effect estimates (β coefficients; printed in bold) and 99% confidence intervals obtained from polynomial linear mixed models for the associations between non-modifiable risk factors (from a child’s perspective) and BMI at the age of 3, 6, 9, 12 and 15 (IDEFICS/I.Family study, eight European countries, 2007–2014).

	Educational level	3 years			6 years			9 years			12 years			15 years		
		β	LCL	UCL	β	LCL	UCL	β	LCL	UCL	β	LCL	UCL	β	LCL	UCL
**Family weight status**																
Family history of obesity (yes vs. no)	Low/medium	**0.28**	−0.11	0.67	**0.71**	0.48	0.95	**1.12**	0.82	1.42	**1.42**	0.99	1.84	**1.50**	0.73	2.27
	High	**0.09**	−0.18	0.35	**0.32**	0.14	0.50	**0.60**	0.37	0.82	**0.82**	0.49	1.15	**0.83**	0.23	1.43
Maternal BMI (kg/m^2^)	Low/medium	**−0.01**	−0.05	0.02	**0.05**	0.04	0.07	**0.11**	0.08	0.14	**0.13**	0.10	0.15	**0.10**	0.05	0.15
	High	**0.03**	0.00	0.05	**0.07**	0.05	0.08	**0.13**	0.11	0.15	**0.15**	0.12	0.17	**0.05**	0.00	0.10
**Pre-natal factors**																
Smoking during pregnancy (Never/rarely vs. several occasions a week/daily)	Low/medium	**−0.08**	−0.52	0.37	**0.10**	−0.18	0.37	**0.19**	−0.16	0.54	**0.51**	0.00	1.02	**1.40**	0.43	2.37
	High	**0.09**	−0.49	0.67	**0.18**	−0.14	0.49	**0.28**	−0.13	0.68	**0.45**	−0.14	1.04	**0.72**	−0.42	1.87
Weight gain during pregnancy (kg)	Low/medium	**0.04**	0.01	0.06	**0.02**	0.01	0.04	**0.02**	−0.01	0.04	**0.02**	−0.01	0.05	**0.03**	−0.03	0.09
	High	**0.03**	0.01	0.05	**0.03**	0.02	0.05	**0.04**	0.02	0.06	**0.06**	0.03	0.09	**0.09**	0.04	0.13
**Peri/post-natal factors**																
Pre-term delivery (yes vs. no)	Low/medium	**−0.31**	−0.80	0.19	**0.01**	−0.28	0.30	**0.10**	−0.27	0.47	**0.35**	−0.18	0.88	**1.23**	0.28	2.18
	High	**−0.29**	−0.63	0.05	**−0.17**	−0.39	0.06	**−0.19**	−0.48	0.10	**−0.11**	−0.54	0.32	**0.40**	−0.42	1.22
Birth weight (1 unit∼100 g)	Low/medium	**0.06**	0.03	0.09	**0.05**	0.03	0.07	**0.04**	0.02	0.06	**0.04**	0.01	0.07	**0.05**	0.00	0.11
	High	**0.06**	0.04	0.08	**0.05**	0.04	0.07	**0.06**	0.04	0.08	**0.06**	0.04	0.09	**0.04**	−0.01	0.08
Total breast feeding (in months)	Low/medium	**0.02**	0.00	0.04	**−0.01**	−0.02	0.01	**−0.01**	−0.03	0.01	**0.00**	−0.03	0.02	**−0.01**	−0.06	0.05
	High	**−0.01**	−0.03	0.01	**−0.01**	−0.02	0.00	**−0.02**	−0.03	−0.01	**−0.02**	−0.05	0.00	**−0.01**	−0.05	0.03
Age at introduction of solid foods (month)	Low/medium	**−0.02**	−0.10	0.05	**−0.02**	−0.06	0.02	**−0.01**	−0.06	0.04	**−0.01**	−0.08	0.06	**−0.06**	−0.20	0.09
	High	**−0.01**	−0.06	0.05	**0.02**	−0.01	0.05	**0.03**	−0.01	0.08	**0.04**	−0.02	0.11	**0.06**	−0.06	0.18
**Family structure/vulnerabilities**																
Migrant status (yes vs. no)	Low/medium	**0.17**	−0.11	0.45	**0.29**	0.02	0.56	**0.63**	0.25	1.00	**0.61**	0.12	1.11	**−0.17**	−1.10	0.77
	High	**−0.02**	−0.26	0.21	**0.03**	−0.20	0.25	**0.04**	−0.25	0.34	**0.19**	−0.23	0.60	**0.46**	−0.32	1.23
Number of children	Low/medium	**0.07**	−0.11	0.26	**−0.07**	−0.14	0.01	**−0.10**	−0.18	−0.01	**−0.04**	−0.17	0.08	**0.08**	−0.28	0.43
in household	High	**−0.03**	−0.16	0.10	**−0.09**	−0.16	−0.03	**−0.16**	−0.22	−0.09	**−0.11**	−0.22	−0.01	**0.15**	−0.13	0.43
Only child (yes vs. no)	Low/medium	**−0.14**	−0.44	0.17	**0.14**	−0.02	0.31	**0.19**	0.00	0.39	**0.10**	−0.15	0.35	**−0.12**	−0.70	0.47
	High	**−0.05**	−0.28	0.18	**0.17**	0.03	0.32	**0.35**	0.19	0.52	**0.39**	0.17	0.61	**0.13**	−0.35	0.61
One-parent family (yes vs. no)	Low/medium	**−0.35**	−1.16	0.46	**0.00**	−0.28	0.29	**0.16**	−0.12	0.45	**0.00**	−0.51	0.51	**−0.56**	−2.44	1.31
	High	**−0.05**	−1.17	1.07	**−0.17**	−0.57	0.23	**−0.29**	−0.63	0.06	**0.06**	−0.53	0.65	**1.34**	−1.08	3.76
Unemployment (yes vs. no)	Low/medium	**−0.10**	−0.47	0.27	**0.02**	−0.10	0.15	**0.22**	0.07	0.37	**0.48**	0.21	0.75	**0.83**	0.01	1.64
	High	**−0.07**	−0.47	0.32	**0.05**	−0.09	0.19	**0.15**	−0.01	0.30	**0.12**	−0.13	0.38	**−0.20**	−0.88	0.47

LCL, lower 99% confidence limit; UCL, upper 99% confidence limit.

Continuous variables were centred to their rounded mean: maternal BMI centred to 23 kg/m^2^, weight gain during pregnancy centred to 14 kg, birth weight centred to 3,300 g and divided by 100, total breast feeding duration centred to 7 months, age at introduction of solid foods centred to 5 months, number of children in household centred to 2.

Separate models were estimated for each exposure. Models for the different exposures were adjusted for confounders as detailed in Supplementary Material S4. Cells marked in grey indicate effect estimates where the 99% confidence interval does not contain the zero.

Differences between the low/medium vs. high parental education groups were further observed with regard to family structure and vulnerabilities: migrant status (up to age 12) and unemployment (ages 9–15) showed positive associations with BMI in the low/medium education group only. Being an only child was associated with a higher BMI in the high education group, but effect estimates pointed to the same direction in the low/medium education group. A larger number of children in the household was associated with a lower BMI in both education groups.

#### Modifiable Risk Factors

Associations of wellbeing and the behavioural risk factors with BMI were in general small and mainly appeared beyond the age of 9 years (Table 3). Again, associations with BMI were similar comparing the two education groups, except for some of the dietary variables. In both education groups, longer sleep duration, membership in a sports club and active transport were associated with a lower BMI at age 9 and/or 12 years. The number of media in the bedroom was positively associated with BMI at ages 6 and/or 9 and PC/TV time from ages 9 to 15. Regarding the dietary variables, some effect estimates changed direction between age 9 and 15. For example, water consumption was positively associated with BMI at age 9 and negatively associated at age 15 while added sugar food consumption and savoury snack/fast food (low/medium education group only) showed a negative association at age 9 and a positive association at age 15. For vegetable consumption we observed a negative association with BMI in the high education group from ages 9 to 12.

**TABLE 3:** Effect estimates (β coefficients; printed in bold) and 99% confidence intervals obtained from polynomial linear mixed models for the associations between modifiable risk factors and body mass index (BMI) at the age of 3, 6, 9, 12 and 15 years (IDEFICS/I.Family study, eight European countries, 2007–2014).

	Educational level	3 years			6 years			9 years			12 years			15 years		
		β	LCL	UCL	β	LCL	UCL	β	LCL	UCL	β	LCL	UCL	β	LCL	UCL
**Wellbeing**																
Major frustrations (yes vs. no/missing)	Low/medium	**−0.37**	−1.10	0.36	**−0.08**	−0.31	0.14	**0.02**	−0.14	0.19	**0.18**	−0.09	0.45	**0.60**	−0.20	1.41
	High	**−0.31**	−0.94	0.32	**0.08**	−0.10	0.25	**0.05**	−0.09	0.20	**0.10**	−0.13	0.33	**0.70**	−0.03	1.43
Wellbeing score	Low/medium	**−0.01**	−0.04	0.02	**0.00**	−0.01	0.02	**−0.02**	−0.03	−0.01	**−0.02**	−0.04	0.00	**0.06**	0.00	0.12
	High	**0.00**	−0.03	0.02	**0.00**	−0.01	0.01	**−0.01**	−0.02	0.00	**−0.02**	−0.03	0.00	**−0.03**	−0.08	0.02
**Behavioural factors**																
Nocturnal sleep duration (hours/night)	Low/medium	**0.05**	−0.09	0.19	**−0.05**	−0.12	0.02	**−0.10**	−0.41	0.20	**−0.14**	−0.25	−0.04	**−0.14**	−0.43	0.15
	High	**−0.01**	−0.12	0.10	**−0.02**	−0.08	0.04	**−0.09**	−0.22	0.04	**−0.13**	−0.22	−0.04	**−0.15**	−0.38	0.08
Number of media in bedroom	Low/medium	**−0.10**	−0.22	0.02	**0.05**	0.00	0.09	**0.07**	0.03	0.11	**0.03**	−0.03	0.09	**−0.03**	−0.21	0.15
	High	**−0.03**	−0.16	0.09	**0.06**	0.02	0.11	**0.05**	0.02	0.09	**0.01**	−0.04	0.06	**0.04**	−0.10	0.18
Average PC/TV time (h/week)	Low/medium	**−0.01**	−0.03	0.01	**0.00**	−0.01	0.01	**0.01**	0.00	0.02	**0.02**	0.01	0.03	**0.03**	0.01	0.05
	High	**−0.01**	−0.03	0.01	**0.00**	0.00	0.01	**0.01**	0.01	0.02	**0.02**	0.01	0.03	**0.03**	0.01	0.05
Membership in sports club (yes vs. no)	Low/medium	**0.04**	−0.31	0.40	**0.04**	−0.07	0.15	**−0.12**	−0.23	−0.01	**−0.26**	−0.44	−0.07	**−0.19**	−0.76	0.38
	High	**−0.03**	−0.25	0.20	**0.07**	−0.02	0.16	**−0.10**	−0.21	0.00	**−0.20**	−0.37	−0.03	**0.11**	−0.35	0.58
Active transport (yes vs. no)	Low/medium	**0.16**	−0.14	0.46	**−0.06**	−0.18	0.06	**−0.16**	−0.28	−0.03	**−0.12**	−0.33	0.08	**0.04**	−0.57	0.65
	High	**−0.05**	−0.26	0.17	**0.01**	−0.08	0.11	**−0.10**	−0.19	0.00	**−0.18**	−0.33	−0.02	**−0.03**	−0.47	0.41
**Dietary intakes**																
Water (times/day)	Low/medium	**−0.03**	−0.13	0.06	**−0.01**	−0.05	0.03	**0.07**	0.03	0.12	**0.03**	−0.03	0.09	**−0.33**	−0.51	−0.15
	High	**−0.04**	−0.11	0.03	**0.01**	−0.02	0.04	**0.08**	0.04	0.11	**0.02**	−0.03	0.08	**−0.29**	−0.43	−0.15
Fruits (times/day)	Low/medium	**−0.01**	−0.13	0.11	**−0.02**	−0.07	0.03	**0.02**	−0.03	0.07	**0.06**	−0.01	0.12	**0.01**	−0.19	0.21
	High	**−0.03**	−0.13	0.08	**0.00**	−0.04	0.04	**0.01**	−0.03	0.05	**−0.02**	−0.08	0.04	**−0.13**	−0.30	0.03
Vegetables (times/day)	Low/medium	**0.05**	−0.11	0.21	**−0.01**	−0.07	0.05	**−0.03**	−0.09	0.02	**−0.04**	−0.10	0.02	**−0.09**	−0.28	0.10
	High	**−0.02**	−0.12	0.09	**0.01**	−0.03	0.06	**−0.09**	−0.14	−0.05	**−0.13**	−0.19	−0.07	**0.10**	−0.04	0.24
Sweetened drinks (times/day)	Low/medium	**−0.03**	−0.10	0.05	**−0.01**	−0.04	0.02	**−0.03**	−0.06	0.00	**0.02**	−0.01	0.05	**0.15**	0.05	0.25
	High	**−0.05**	−0.12	0.02	**−0.02**	−0.05	0.01	**0.00**	−0.04	0.03	**0.03**	−0.01	0.06	**0.08**	−0.02	0.18
Savoury fast or snack food (times/day)	Low/medium	**−0.09**	−0.35	0.16	**−0.08**	−0.16	0.00	**−0.09**	−0.15	−0.02	**0.01**	−0.07	0.10	**0.36**	0.08	0.63
	High	**−0.02**	−0.23	0.18	**−0.08**	−0.16	0.00	**−0.01**	−0.07	0.06	**−0.02**	−0.11	0.08	**−0.32**	−0.67	0.03
Added sugar foods (times/day)	Low/medium	**−0.03**	−0.16	0.09	**−0.03**	−0.08	0.02	**−0.07**	−0.12	−0.02	**−0.01**	−0.08	0.06	**0.27**	0.08	0.46
	High	**−0.13**	−0.23	−0.02	**−0.04**	−0.09	0.02	**−0.07**	−0.13	−0.02	**−0.10**	−0.18	−0.02	**0.01**	−0.24	0.27

LCL, lower 99% confidence limit; UCL, upper 99% confidence limit.

Continuous variables were centred to their rounded mean: wellbeing score centred to 40, sleep duration centred to 10 h/night, average PC/TV time centred to 14 h/week, water consumption centred to 3 times/day, fruits to 2 times/day, vegetables to 1 time/day, sweetened drinks to 2 times/day, simple sugar foods to 1 time/day.

Separate models were estimated for each exposure. Models for the different exposures were adjusted for confounders as detailed in Supplementary Material S4. Cells marked in grey indicate effect estimates where the 99% confidence interval does not contain the zero.

#### BMI Trajectories at Selected Exposure Levels

**FIGURE 3:**
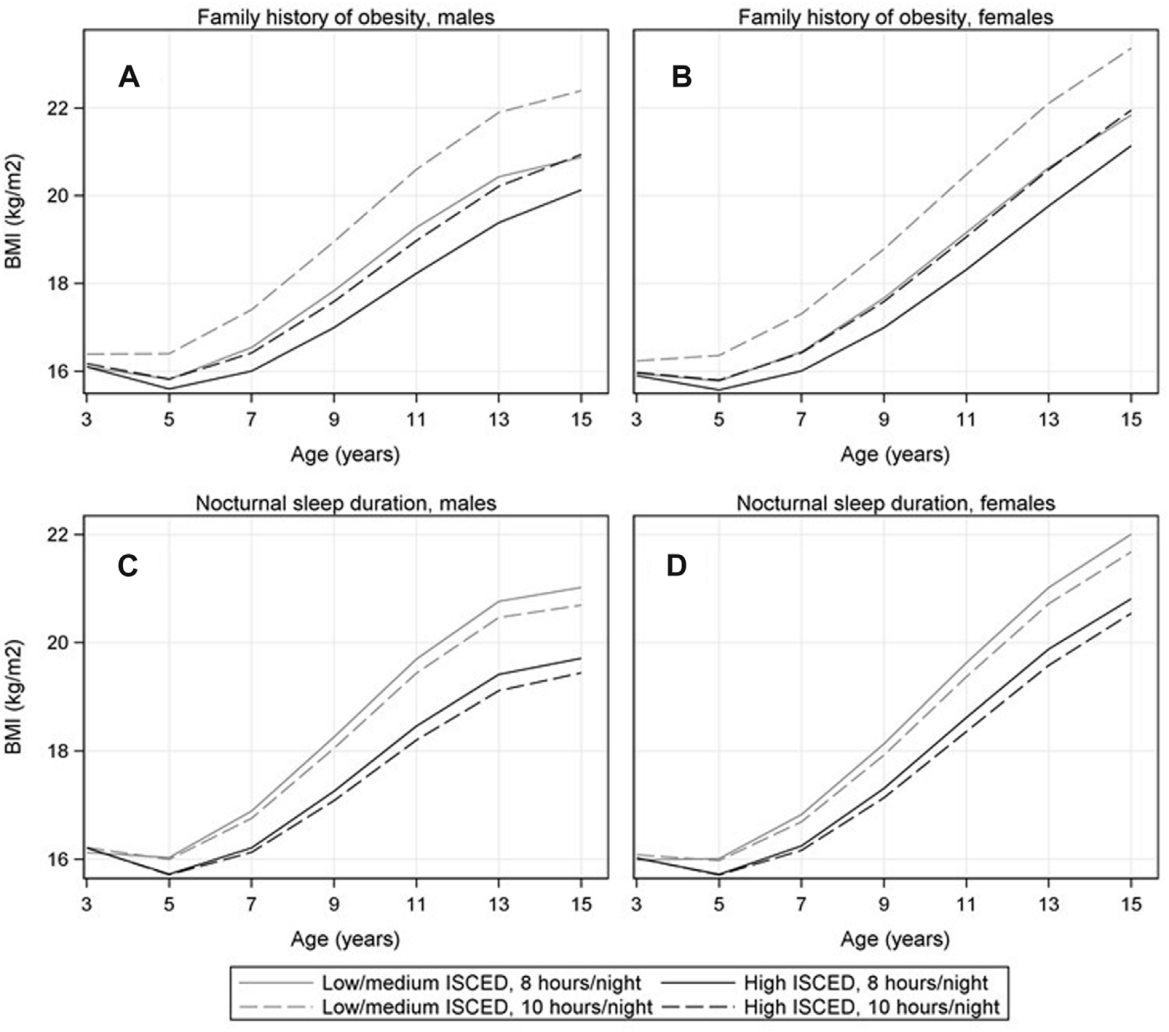
Mean BMI trajectories comparing children with vs. without family history of obesity [panel **(A)** in males, panel **(B)** in females] and BMI trajectories comparing children that sleep 8 vs. 10 h/night [panel **(C)** in males; panel **(D)** in females] stratified by educational level of parents (low/medium vs. high ISCED level). The numbers represent the marginal means estimated based on the confounder-adjusted fractional polynomial mixed effects models, i.e., the mean values at fixed exposure levels were standardized over covariates (IDEFICS/I.Family study, eight European countries, 2007–2014).

Figure 3 and Supplementary Material S6 compare BMI trajectories of children at certain fixed exposure levels (e.g., 8 h vs. 10 h nocturnal sleep duration) while standardizing over all covariates. In general, the BMI trajectories were higher in the low/medium vs. high education group. Large differences appeared when comparing children with vs. without family history of obesity (see Figures 3A, B) whereas for most behavioural factors the observed differences were rather small within the same education group (see, e.g., Figures 3C, D for sleep duration, and Supplementary Material S6 for all other exposures).

## Discussion

We studied a wide range of risk factors for OW/OB during the early life course as well as differences by educational level of parents. Our results confirm that large differences exist concerning the age-specific prevalence of risk factors during childhood/adolescence comparing children of parents with a low/medium vs. high educational level. Further differences exist between boys and girls. The higher prevalence of OW/OB risk factors in children with low/medium parental education is well documented in previous literature [28] and is likely to explain the higher OW/OB prevalence in that group. Nevertheless, the associations of most risk factors with BMI are similar among children of both education groups. This suggests that a risk factor, if present, will have its detrimental effect on BMI independently of the parental educational level.

### Non-Modifiable Risk Factors

Our data confirm the effects of well-established risk factors acting early in life, e.g., for high pregnancy weight gain [29], high birth weight, and high maternal pre-pregnancy BMI [30, 31], and further demonstrate the persistence of these effects into adolescence. For example, our data reveal maternal weight gain during pregnancy and maternal BMI to be associated with an increased BMI even 15 years after birth.

### Modifiable Risk Factors

In line with previous studies, membership in a sports club and active transport [32] as proxies for physical activity were negatively associated with BMI while higher screen time [33] as proxy for sedentary behaviour was positively associated with BMI. These associations were observed from the age of 9–12 years onwards and independent of the parental educational level. Moreover, sleep duration turned out to be a protective factor for BMI, supporting previous studies [34]. A recent study that did not observe effects of behavioural factors on adiposity in children up to the age of 5 years suspected that young children were less likely to, e.g., consume fast food or engage in PA [35]. Consequently, the lower variability in such health behaviours may make it more difficult to detect effects on adiposity in young children. This may explain why associations of behavioural factors with BMI were mainly observed in later childhood/adolescence in our study.

Some of the effect estimates of the dietary variables changed their direction at approximately 12 years of age which corresponds to the age when we switched from parental reports to self-reports. For fruit and vegetable consumption, parent and child reports were previously shown to differ at the individual, but not at the group level [36]. Consequently, the authors of that study concluded that parent and child reports can be used in determinant studies. Nevertheless, we cannot preclude that our findings may be partially explained by differences in social desirability bias and differential reporting behaviour in parents and teens [37, 38]. In addition, dietary data are particularly prone to misreporting [37] which may be another reason for the partly unexpected findings for water, added sugar foods and savoury fast/snack foods.

Beyond that, our estimated risk factor trajectories indeed point to the age period of 9–11 years being a potential turning point for many health behaviours. Certain risk factors such as average PC/TV time, consumption frequency of sweetened drinks and savoury fast and snack foods increased after the age of 10. This may be explained by increased peer influence and stronger autonomy of children getting older. A recent paper by [39] revealed that the peer resemblance of health behaviours surpasses the sibling resemblance by the age of 9–10 years. Moreover, use of digital media increases with age. This may lead to a higher exposure to advertisements for unhealthy foods, which in turn is associated with less healthy food and beverage intake [40, 41].

### Sex-Specific Differences in Risk Factor Trajectories

A sports club membership was more common in boys than in girls in our study, which has been previously observed across European countries [42]. One reason may be an insufficient variety of activities that suits girl’s preferences [42]. Boys also spent more time with PC/TV than girls which was also reported for primary school children across Europe. Different cultural values regarding sedentary behaviours of boys and girls may be responsible for this difference which in turn influence parental support [42]. Again in agreement with other studies, the consumption of sweetened drinks was higher among boys than girls [43–45] which may partly be explained by the higher screen time of boys [40, 45]. In our study, at the ages of 9–11 years, the mean wellbeing score increased in boys while it decreased in girls. A study in 11 to 15 years-old German adolescents also reported lower life satisfaction and self-related health in girls than in boys. Furthermore, girls reported psychosomatic health complaints more often than boys with the frequency increasing with age [46]. Potential reasons for this gap may be sex-specific differences in pubertal development, e.g., physical changes and the development of self-identity as well as different coping strategies for increased school stress of boys and girls [46].

### Social Vulnerability and Family Structure

Previous studies showed social vulnerabilities such as unemployment [47] and having a migration background [48] to be risk factors for childhood OW/OB. We found unemployment and migration background to be associated with a higher BMI particularly in children with low/medium parental education. It has been suggested that the socio-economic position of parents may exacerbate or buffer the effect social vulnerabilities may have on different health-related behaviours and stress [28, 48]. Unemployment affects not only the economic resources but reduces social integration and life satisfaction. A migration background can result in language barriers and a lack of acceptance by people of the new country which are stressors for migrant children [48]. Furthermore, risk factors for OW/OB like unfavourable dietary patterns, shorter sleep duration [49] and sedentary behaviours have been reported to be more prevalent among children with a migration background [50]. Nevertheless, the OW/OB risk can vary depending on the reasons of migration, the similarity with the host country and the language spoken at home [48] which may be causes that could explain why the associations are stronger in the low/medium ISCED group. Further social vulnerabilities which have previously been shown to increase the risk of childhood OW/OB include living in a single parent family [51] or being an only child [52]. In our study only the latter was found to be associated with the children’s BMI and this association was stronger in the high compared to the low/medium education group. In line with the literature [53] we found a higher number of children in the household to decrease the risk for childhood OW/OB. This association was not observed at the age of 3 years when siblings may not have been born yet for many children and not at the age of 15 years when siblings may have already moved out or become less important because of a higher degree of independence.

In general, associations between the single behavioural factors and BMI were rather small. Nevertheless, multi-factorial interventions addressing several modifiable factors may still have a large impact on BMI development as confirmed by previous meta-analyses [16, 54].

### Limitations and Strengths

We acknowledge several limitations: Although information on the type of occupation and family income would have been available, we focussed on differences by parental educational level in our study. Employment status was previously shown to be less related to childhood OW/OB [5] and reported income is known to be more prone to misreporting and typically contains many missing values (as also in our data). In addition, educational level may not only reflect the socio-economic status but is further a marker for health literacy [55] which is likely to be linked to many modifiable OW/OB risk factors [56]. Highly educated parents and their children were more likely to participate in our cohort, i.e., selection towards a healthier, well-educated population is likely [57].

All exposure variables except for maternal BMI at W2 were based on self- or proxy-reports and may hence be affected by recall and social-desirability bias where the latter may even differ between self-reports vs. parental reports. We further used rather simple indicators to reflect dietary intakes of foods considered healthy/unhealthy to ease interpretation, but these cannot capture the complexity of the diet as a whole. The use of polynomial models with three powers of age is still a compromise between model complexity, adequate model fit and interpretability. As we modelled mean BMI trajectories starting after an age of 2 years and hence with only one local minimum (adiposity rebound) we considered models with three powers of age to provide sufficient flexibility [26].

Strengths of our study include the highly standardized and quality-controlled assessment procedures, the large multi-centre study with a prospective design, the use of a DAG for confounder selection and the sophisticated statistical methods used to estimate the exposure trajectories and their associations with BMI covering the early life course from infancy to adolescence. Our study is unique in that it considered a large number of known and suspected OW/OB risk factors simultaneously while adjusting for a comprehensive set of confounders. The overall credibility of our results is supported by the fact that associations with most well-established risk factors for OW/OB are replicated by our analysis.

### Conclusion

The age-specific prevalence of many OW/OB risk factors largely differs by educational level of parents, especially with view to dietary variables. At approximately 9–11 years of age certain risk factors like the consumption of sweetened drinks and savoury fast and snack foods markedly increase. This may be explained by increased peer influence and stronger autonomy as well as by higher exposure to advertisements for unhealthy foods as children grow older. Sex-specific differences were found for PC/TV use that increases much stronger in boys than in girls and for wellbeing that decreases in girls while it increases in boys beyond the age of 11 years. Associations of risk factors with BMI trajectories did not essentially differ by parental educational level except for social vulnerabilities like migration status and unemployment which seem to exert more detrimental effects in children of parents with low/medium education. BMI trajectories also differed between children with vs. without a family history of obesity, while modifiable behavioural factors showed mainly small associations with BMI. Although the single effects may appear small, multi-factorial interventions addressing several modifiable factors may still have a large impact on BMI development.
