# Supplementary material for: Age-Specific Quantification of Overweight/Obesity Risk Factors From Infancy to Adolescence and Differences by Educational Level of Parents
Source: Int J Public Health. 2023 Nov 15;68:1605798. doi: 10.3389/ijph.2023.1605798 (PMC10684735; doi:10.3389/ijph.2023.1605798)
Supplement: Supplementary file 1 [file DataSheet1.PDF]

International Journal of Public Health

Article: Age-specific quantification of overweight/obesity risk factors from infancy to adolescence and differences by educational level of parents.

Online Resources

This document contains the following online resources:

- Supplementary Material S1: Description of exposures
- Supplementary Material S2: Directed acyclic graph (DAG) depicting the causal relations assumed among various risk factors for obesity and BMI
- Supplementary Material S3: Details on multiple imputation
- Supplementary Material S4: Flow chart depicting selection process leading to final study sample
- Supplementary Material S5: Table listing the adjustment sets for the 26 exposures
- Supplementary Material S6: Figures and tables depicting the marginal mean values of the time-varying exposures from infancy to adolescence
- Supplementary Material S7: Figures and tables depicting the marginal mean values of BMI from infancy to adolescence at fixed exposure levels

Supplementary Material S1: Description of exposures

Description of obesity risk factors

Unless otherwise stated, exposure/covariate information was obtained from parentally reported questionnaires or self-reported questionnaires (in children/teens ≥ 12 years) completed at the W0, W1 and W2 surveys. Questions on pregnancy-related variables were posed to biologic mothers only. Several variables were dichotomized in order to reduce model complexity considering the large number of included risk factors.

Non-modifiable factors

Age (in years), sex (female; ref male), region of residence (North/Central Europe including Belgium, Sweden and Germany, Southern Europe including Cyprus, Italy, Spain and Eastern Europe including Estonia and Hungary), family history of obesity (diagnosed obesity in at least one parent, grandparent or sibling; yes vs no) and migrant status (at least one parent born in foreign country vs both parents born in the country of residence) were considered as non-modifiable risk factors in the analyses.

Educational level of parents

Highest educational level of parents was categorized according to the International Standard Classification of Education (ISCED) [1]. The maximum ISCED level of both parents was dichotomized (low/medium=ISCED levels 0,1,2,3,4,5 vs high=ISCED levels 6,7,8) and used for stratification in the analyses.

Early life factors

For early life factors, information was assessed in W0 via questionnaires and updated by W1 values in case of missing W0 data.

Maternal body mass index (BMI; kg/m^2): Maternal BMI at W0, W1 and W2 was calculated as weight (kg) divided by height (m) squared where weight/height were self-reported in W0 and W1 and measured in W2.

Smoking of mother during pregnancy: For smoking frequency answer categories ranged from 'Never', 'Rarely, at max once a month', 'Several occasions a week' to 'Daily'. To enhance stability of model estimates, the original smoking categories were dichotomized into 'Never/rarely' vs. 'At least several occasions a week/daily'.

Weight gain during pregnancy (kg): Information was obtained from biological mothers via questionnaires.

Gestational age of new-born at delivery: A binary indicator was constructed for children delivered at term vs. children born pre-term (≤ 37th gestational week; yes vs no), information was obtained from biological mothers via questionnaires.

Birth weight (g) of the child was reported by mothers.

Total breastfeeding duration: Starting and ending months of exclusive breastfeeding and breastfeeding combinations with solid foods and/or formula milk were used to derive the total breastfeeding duration.

Age at introduction of solid foods (month): This variable provides information on the earliest age at which one of the following food items were introduced: cereals, vegetables, fruits, meat, cow milk.

Family structure/vulnerabilities

Number of children in household: Information on the number of younger siblings, older siblings and siblings of the same age living in the household was used to calculate the number of children in household (number of siblings plus the child itself) in W0 and W1. In W2 the number of persons in the household below the age of 18 was queried in the questionnaire and used correspondingly as indicator of the number of children in the household.

Being an only child (yes vs no): Information on the number of siblings was used to derive an indicator for a child being an only child at W0, W1 and W2.

One-parent family (yes vs no): In W0 and W1 the child was considered to live in a single parent family in case it was stated in the questionnaire that the child lives either most of the time with the mother or with the father. In W2, single parenthood was assumed if the child lives only with one parent (step-parent or biological parent in the household) but no other adult except for older siblings. This information was obtained from an interview with an adult household member who was asked for the relationship of all household members to the child and whether the child lives part-time in another household.

Unemployment (yes vs no): Unemployment was assumed if either the mother or the father answered that he/she is currently unemployed, for a year or more.

Consumer attitudes

Answers on the following statements were dichotomized (Disagree/moderately disagree/unsure vs Moderately agree/agree) to obtain indicators on consumer behaviour and food literacy: “I compare labels to select most nutritious food”, “I have more confidence in food products that I have seen advertised than in unadvertised foods” and “We use a lot of ready-to-eat foods in our household”. The variables were used as covariates for adjustment.

Family life

In W0 and W1, the following statement was dichotomized (Not true/hardly true vs Rather true/exactly true) and used as indicator for family life: “We often go on trips together”. In W2 we used information on the question “How often do you do some of the following things with your parents - Take trips, travel, or go visiting?” (dichotomized as Never/About once a year vs About once a month/About once a week/Everyday as proxy for the statement in W0/W1.

The statement “At home it is laid down quite exactly what is allowed and what not” was dichotomized (Not true/hardly true vs Rather true/exactly true) in W0/W1 as proxy for family rules. In W2 we derived a score based on answers on 7 questions concerning rules about TV and other media use. If the parent answered to have at least 3 out of the 7 rules, it was considered comparable to the W0/W1 statement of having laid down quite exactly what is allowed at home and what not. The variables were used as covariates for adjustment.

Family food environment

The following dichotomized variables were used to reflect the family food environment: “The kids help in the kitchen, e.g. they peel the potatoes and cut the vegetables” (Disagree/moderately disagree/unsure vs Moderately agree/agree), “Parent sits down with child when eating meals” (Never/rarely/sometimes vs Often/always), “Frequency of child eating while doing something else, e.g. watching TV, playing, sitting at a computer, looking at a book” (Never or rarely/several times per week vs Once a day/on several occasions per day). The variables were used as covariates for adjustment.

Well-being

Well-being score: Psychosocial well-being was measured with 16 items of four subscales of the “KINDL-R Questionnaire for Measuring Health-Related Quality of Life (HRQoL) in Children and Adolescents” (emotional well-being, self-esteem, family life and relations to friends) [2; 3]. At W2, response categories corresponded to the original 5-point Likert scale (never, seldom, sometimes, often, all the time). At W0 and W1 the two highest response categories were combined into one category. Therefore, we deviated from the original scoring (1-5 points per item) and assigned 0 points for “Never” and 3 points for both “Often” and “All the time” (at follow-up) or “Often/All the time” (at baseline), respectively (six negatively worded items were coded reversely). Consequently, the score ranged from 0-48 with a higher score indicating a higher well-being.

As additional indicator for well-being we included the following: “Child encountered major frustrations, e.g. at school or with peers” (yes vs no/missing).

Behavioural factors

Nocturnal sleep duration (hours/night): At W0, information on sleep duration was collected in the context of a standardized 24-h recall. Next to questions on dietary intakes, parents were asked about their child's get up time in the morning as well as bed time (hour/minute) of the previous day. Nocturnal sleep duration was calculated as difference between bed time and get up time resulting in a continuous estimate of sleep hours per night as described previously [4]. At W1 and W2, participants reported sleep duration in hours and minutes in self-completion questionnaires, i.e. the instructions read as follows: "What is the amount of time the child sleeps during a 24-hour period on weekdays? Give separate information for night time sleep and naps in the daytime." Analogously, information was collected for weekend days/vacations. The weighted average of nocturnal sleep duration was calculated as follows: (nocturnal sleep duration on weekdays*5 + nocturnal sleep duration on weekend days*2) / 7.

Number of media in bedroom: The number of media devices reported to be in children's'/teens' bedroom was used as an indicator for media use [5].

Average PC/TV time (hours/week): The time spent with audiovisual media was calculated based on the reported hours/minutes watching TV/video/DVD and hours/minutes sitting in front of a computer/game console. A weighted average over weekdays and weekend days was calculated.

Physical activity (hours per week) was calculated based on reported hours/minutes playing outdoors and hours/minutes spent in a sports club. The weighted average over weekday and weekend days was used.

Membership in sports club (yes vs no): A variable indicating whether the child was member in a sports club was used as another proxy for physical activity.

Active transport (yes vs no): Active transport to/from kindergarten or school was considered in case the child usually gets to and from kindergarten/school either walking or cycling.

Dietary variables

In W0, W1 and W2 information on food frequencies were assessed based on the so-called Children's Eating Habits Questionnaire–food frequency section (CEHQ-FFQ). The CEHQ-FFQ was designed as a screening tool to assess eating behaviours associated with risk of overweight, obesity and general health in children. It covered 43 food times in W1, 46 in W2 and 58 in W3 with the following answer categories: 'never/less than once a week', '1–3 times a week', '4–6 times a week', '1 time per day', '2 times per day', '3 times per day', '4 or more times per day' and in W1 and W2 'I have no idea'. These categories were converted into

times per week ranging from 0 up to 30. Based on the converted food consumption frequencies, we calculated the following variables that were used in the present analyses:

- *Water consumption (times/day)*
- *Fruit consumption (times/day):* Sum of fresh fruits without sugar added and fresh fruits with sugar added
- *Vegetable consumption (times/day):* Sum of cooked vegetables, legumes, and raw vegetables
- *Sweetened drinks (times/day):* In W0 and W1, sum of fruit juices, sweetened drinks including sports drinks, bottled or canned tea, syrup-based drinks and similar, diet coke or diet soft drinks and sweetened milk; in W2 additionally carbonated sugar sweetened drinks, artificially sweetened drinks, sweetened coffee and sweetened tea.
- *Savoury fast and snack food (times/day):* Sum of pizza as main dish, hamburger, hot dog, kebab, wrap, falafel, snacks like crips, corn crips, popcorn and snacks like savoury pastries and fritters.
- *Added sugar foods (times/day):* Sum of snacks like chocolate, candy bars, candies, loose candies, marshmallow, biscuits, packaged cakes, or pastries and puddings, ice cream, milk- or fruit-based bars.

Pubertal status: Only at W3, pubertal status (yes vs no; yes if menarche had already occurred in girls or if voice alterations had already started or were completed in boys) was self-reported by children 8 years and older based on questions adapted from Carskadon and Acebo [6].

Supplementary Material S2

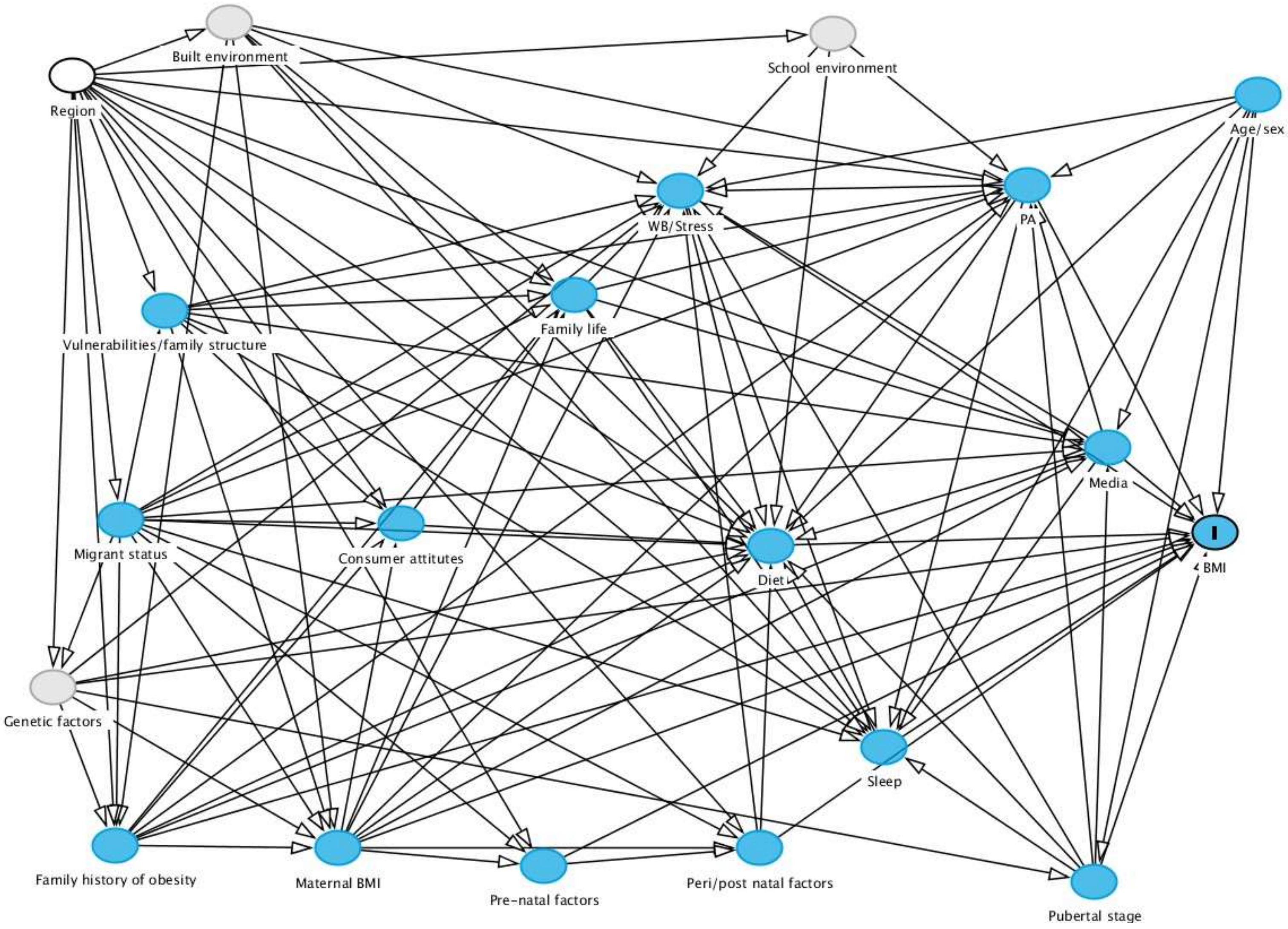

Figure S2: Directed acyclic graph (DAG) depicting the causal relations assumed among various risk factors for obesity and BMI. The DAG was build using DAGitty (http://www.dagitty.net/). Some associations are likely to be bi-directional (e.g. well-being and sleep duration). In such cases we selected the association for which most research evidence exists or the one for which the association was assumed to be stronger. Some variables were further grouped (e.g. dietary variables like water consumption, added sugar food, etc were grouped as “Diet”) to reduce the complexity of the DAG. The DAG further includes three unobserved factors indicated in grey – genetic factors, built environment and school environment.

Supplementary Material S3

Multiple imputation

For the multiple imputation (MI), data were arranged in wide format, i.e. a new variable is built for each repeated measurement (W0, W1, W2). Then standard FCS (fully conditional specification) MI was performed with 10 replicates. All outcomes and exposures at the different assessment waves used in the final analyses were included in the MI procedure. The percentages of missing values ranged from 0% (age, sex) up to 42% (sleep duration at W0) depending on the variable considered. For the majority of variables less than 5% of the values were missing. The outcome did not contain any missing values. In case data of a complete assessment wave were missing for a participant, that (imputed) wave was not considered in the later analysis. When imputing the data of W2, the imputation dataset contained only children participating at W2 and their data from W0 and W1 in order not to reduce the efficiency of the imputations due to the large number of children not providing data in W2. The relative efficiency was >96% for all variables indicating good imputation quality.

Huque et al. [7] recently compared seven MI methods for handling missing values in longitudinal and clustered data in the context of fitting linear mixed models (LMM) with both random intercepts and slopes. They demonstrated that standard FCS outperformed even LMM-based MI approaches if measurements occur at the same time points for all individuals in longitudinal studies, especially in case only the covariates (but not the outcome) is imputed. This corroborated the choice of using standard FCS in our setting.

Supplementary Material 4

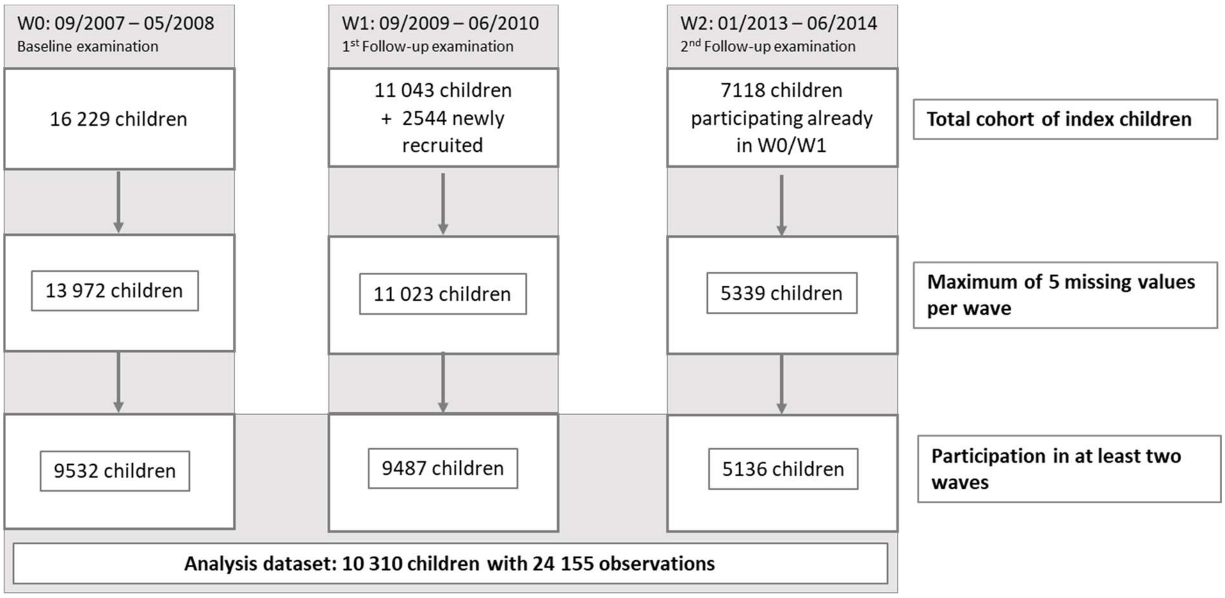

Figure S4: Flow chart depicting the selection process leading to the final study sample.

Supplementary Material S5

Exposure	Adjustment set
Family weight status	
Family history of obesity	Migrant status, region, *built environment, genetic factors*
Maternal BMI	Migrant status, family history of obesity, one-parent family, number of children, unemployment, region, *genetic factors, built environment*
Pre-natal factors	
Smoking during pregnancy	Maternal BMI, migrant status, region
Weight gain during pregnancy	Smoking during pregnancy, maternal BMI, migrant status, region
Peri/post-natal factors	
Pre-term delivery	Weight gain, smoking during pregnancy, maternal BMI, migrant status, region
Birth weight	Pre-term delivery, weight gain, smoking during pregnancy, maternal BMI, migrant status, region
Total breastfeeding	Birth weight, pre-term delivery, weight gain, smoking during pregnancy, maternal BMI, migrant status, region
Age at introduction of solid foods	Total breastfeeding, birth weight, pre-term delivery, weight gain, smoking during pregnancy, maternal BMI, migrant status, region
Family structure/vulnerabilities	
Migrant status	Region
Number of children in household	Migrant status, region
Only child	Migrant status, region
One-parent family	Migrant status, region
Unemployment	Migrant status, region
Well-being	
Major frustrations, e.g. at school	Age/sex, family often goes on trips, rules laid down quite exactly, parent sit down with child while eating, maternal BMI, average PC/TV time, number of media in bedroom, migrant status, sports club membership, active transport, total breastfeeding duration, pubertal stage, region, one-parent family, number of children, unemployment, *built environment, genetic factors, school environment*
Well-being score	Age/sex, major frustrations at school, family often goes on trips, rules laid down quite exactly, parent sit down with child while eating, maternal BMI, average PC/TV time, number of media in bedroom, migrant status, sports club membership, active transport, total breastfeeding duration, pubertal stage, region, one-parent family, number of children, unemployment, *built environment, genetic factors, school environment*
Behavioural factors	
Nocturnal sleep duration	Age/sex, rules laid down quite exactly, parent sit down with child while eating, family often goes on trips, number of media in bedroom, average TV/PC time, migrant status, sports club membership, active transport, pubertal stage, region, one-parent family, number of children, unemployment, well-being score, major frustrations at school, *built environment*
Number of media in bedroom	Age/sex, family history of obesity, rules laid down quite exactly, maternal BMI, migrant status, pubertal stage, region, one-parent family, number of children, unemployment
Average PC/TV time	Age/sex, number of media in bedroom, family history of obesity, rules laid down quite exactly, maternal BMI, migrant status, pubertal stage, region, one-parent family, number of children, unemployment
Membership in sports club	Age/sex, family history of obesity, family often goes on trips, rules laid down quite exactly, parent sits down with child while eating, maternal BMI, media, migrant status, pubertal stage, region, one-parent family, number of children, unemployment, *school environment, built environment*
Active transport	Age/sex, family history of obesity, family often goes on trips, rules laid

	down quite exactly, parent sits down with child while eating, maternal BMI, media, migrant status, pubertal stage, region, one-parent family, number of children, unemployment, *school environment, built environment*
Diet: water	Age/sex, sweetened drinks, family history of obesity, maternal BMI, average PC/TV time, number of media in bedroom, migrant status, club membership, active transport, total breastfeeding duration, introduction of solid foods, pubertal stage, region, sleep duration, well-being score, major frustrations at school, *genetic factors*
Diet: fruits	Age/sex, family history of obesity, maternal BMI, average PC/TV time, number of media in bedroom, migrant status, club membership, active transport, total breastfeeding duration, introduction of solid foods, pubertal stage, region, sleep duration, well-being score, major frustrations at school, *genetic factors*
Diet: vegetables	Age/sex, family history of obesity, maternal BMI, average PC/TV time, number of media in bedroom, migrant status, club membership, active transport, total breastfeeding duration, introduction of solid foods, pubertal stage, region, sleep duration, well-being score, major frustrations at school, *genetic factors*
Diet: sweetened drinks	Age/sex, water, family history of obesity, maternal BMI, average PC/TV time, number of media in bedroom, migrant status, club membership, active transport, total breastfeeding duration, introduction of solid foods, pubertal stage, region, sleep duration, well-being score, major frustrations at school, *genetic factors*
Diet: savoury fast or snack food	Age/sex, family history of obesity, maternal BMI, average PC/TV time, number of media in bedroom, migrant status, club membership, active transport, total breastfeeding duration, introduction of solid foods, pubertal stage, region, sleep duration, well-being score, major frustrations at school, *genetic factors*
Diet: added sugar foods	Age/sex, family history of obesity, maternal BMI, average PC/TV time, number of media in bedroom, migrant status, club membership, active transport, total breastfeeding duration, introduction of solid foods, pubertal stage, region, sleep duration, well-being score, major frustrations at school, *genetic factors*

Table S5: Exposures considered in the analyses and minimal adjustment sets. Variables listed in grey indicated unobserved factors that we were not able to adjust for

The adjustment sets were selected based on the DAG presented in Supplementary Material 2. Unobserved variables that we were not able to adjust for but would have been important are indicated in grey. In case the adjustment set indicated by the DAG contains knots that summarize several variables (e.g. family life), the variable(s) out of that knot considered as most important with regard to the specific exposure-outcome relation were chosen (e.g. from the knot family life, the variable family rules was chosen for adjustment when average TV/PC time was the exposure of interest). Variables within a certain knot are mutually adjusted only in case this is plausible considering the assumed causal relations (e.g. in case of peri-/post-natal factors breastfeeding is adjusted for birth weight but not vice versa).

Supplementary Material S6

The following figures and tables depict the marginal mean values of the time-varying exposures stratified by sex and ISCED level for the ages 3, 5, 7, 9, 11, 13 and 15 years. For instance, on the following page the marginal mean estimates of maternal BMI (kg/m^2) are plotted against age, stratified by sex and ISCED level. The table presents the corresponding numbers as well as lower and upper 95% confidence intervals.

LCL: lower 95% confidence limit

UCL: upper 95% confidence limit

Summary of results

In line with the higher average PC/TV time, also the number of media in the bedroom was higher in boys and in the low/medium education group and increased with age. Similar to the increase of sweetened drinks consumption in all groups, more added sugar foods were consumed from the age of 11 years, however with a decreasing trend in high education girls. Vegetable consumption consistently increased with age and was higher in girls than in boys within both education groups. In parallel with the consistently decreasing well-being in girls, the percentage of major frustrations in school increased with increasing age, particularly in girls and boys in the low/medium education group from the age of 13 years and 9 years, respectively. The percentage of children using an active form of transport to/from school was higher at younger ages in the low/medium education group but increased in the high education group and became higher as compared to the low/medium education group from the age of 9-10 years.

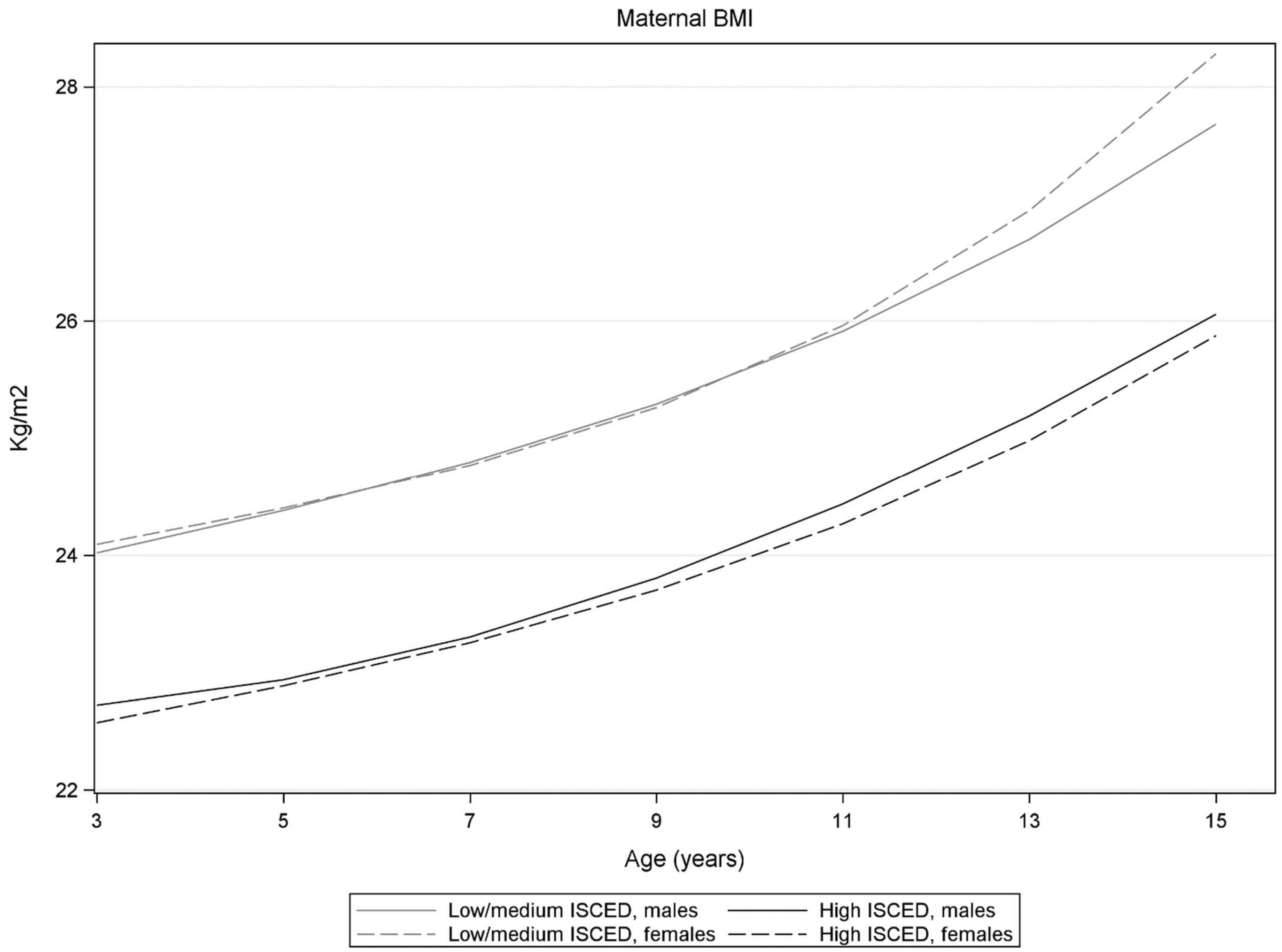

		Maternal BMI (kg/m2)					
		Low/medium ISCED			High ISCED		
**Sex**	**Age**	**Mean**	**LCL**	**UCL**	**Mean**	**LCL**	**UCL**
Male	3	24.0	23.7	24.3	22.7	22.5	22.9
	5	24.4	24.2	24.6	22.9	22.8	23.1
	7	24.8	24.6	25.0	23.3	23.2	23.5
	9	25.3	25.1	25.5	23.8	23.6	24.0
	11	25.9	25.7	26.1	24.4	24.3	24.6
	13	26.7	26.4	27.0	25.2	25.0	25.4
	15	27.7	27.2	28.2	26.1	25.7	26.4
Female	3	24.1	23.8	24.4	22.6	22.4	22.8
	5	24.4	24.2	24.6	22.9	22.7	23.0
	7	24.8	24.6	25.0	23.3	23.1	23.4
	9	25.3	25.1	25.5	23.7	23.5	23.9
	11	26.0	25.7	26.2	24.3	24.1	24.4
	13	26.9	26.7	27.2	25.0	24.8	25.2
	15	28.3	27.8	28.8	25.9	25.5	26.2

Figure and Table: Marginal means of maternal BMI stratified by sex and education level for the ages 3, 5, 7, 9, 11, 13 and 15 years.

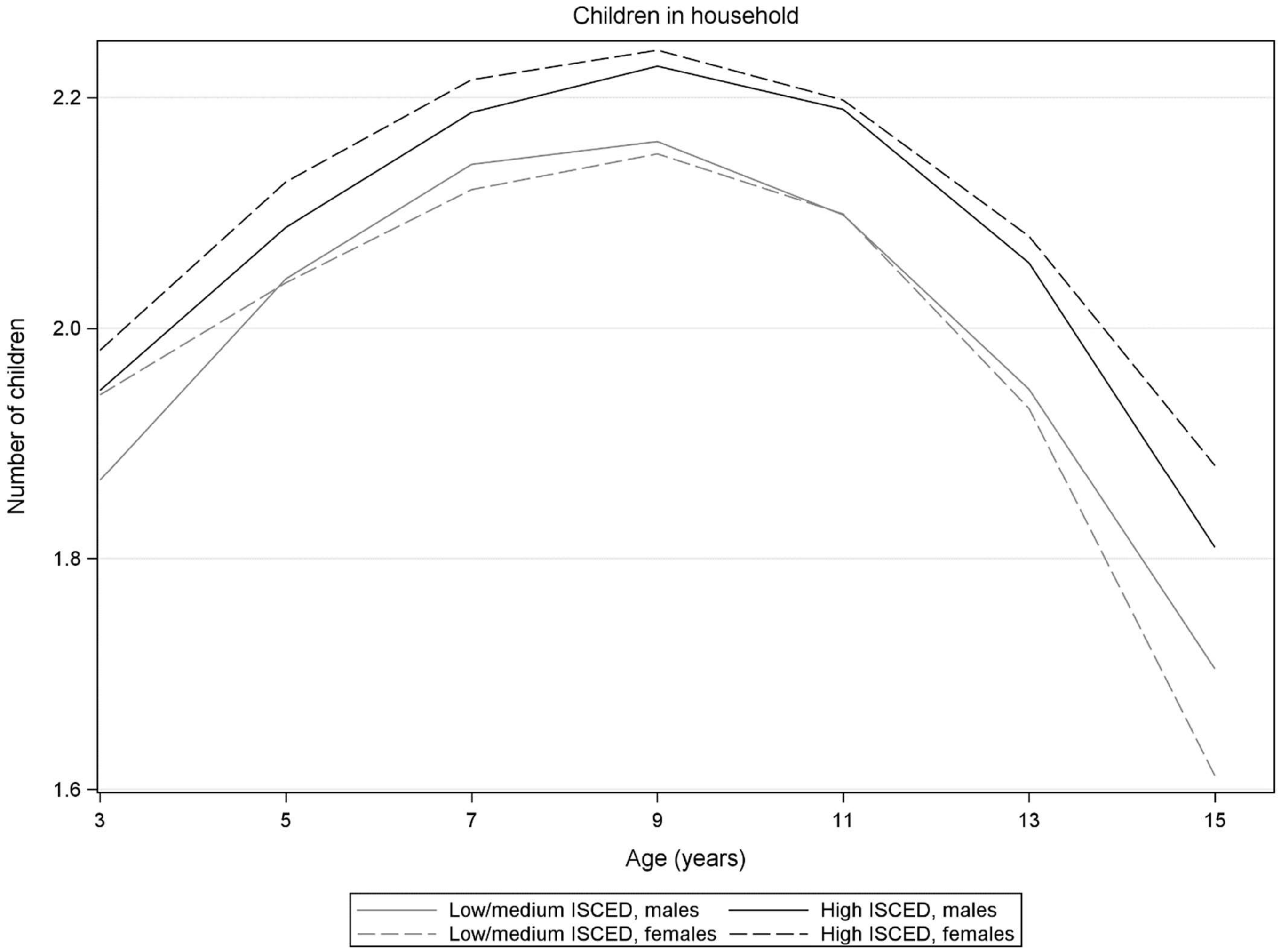

		Number of children in household					
		Low/medium ISCED			High ISCED		
**Sex**	**Age**	**Mean**	**LCL**	**UCL**	**Mean**	**LCL**	**UCL**
Male	3	1.9	1.8	1.9	1.9	1.9	2.0
	5	2.0	2.0	2.1	2.1	2.1	2.1
	7	2.1	2.1	2.2	2.2	2.2	2.2
	9	2.2	2.1	2.2	2.2	2.2	2.3
	11	2.1	2.1	2.1	2.2	2.2	2.2
	13	1.9	1.9	2.0	2.1	2.0	2.1
	15	1.7	1.6	1.8	1.8	1.7	1.9
Female	3	1.9	1.9	2.0	2.0	1.9	2.0
	5	2.0	2.0	2.1	2.1	2.1	2.2
	7	2.1	2.1	2.2	2.2	2.2	2.2
	9	2.2	2.1	2.2	2.2	2.2	2.3
	11	2.1	2.1	2.1	2.2	2.2	2.2
	13	1.9	1.9	2.0	2.1	2.0	2.1
	15	1.6	1.5	1.7	1.9	1.8	2.0

Figure and Table: Marginal means of number of children in household stratified by sex and education level for the ages 3, 5, 7, 9, 11, 13 and 15 years.

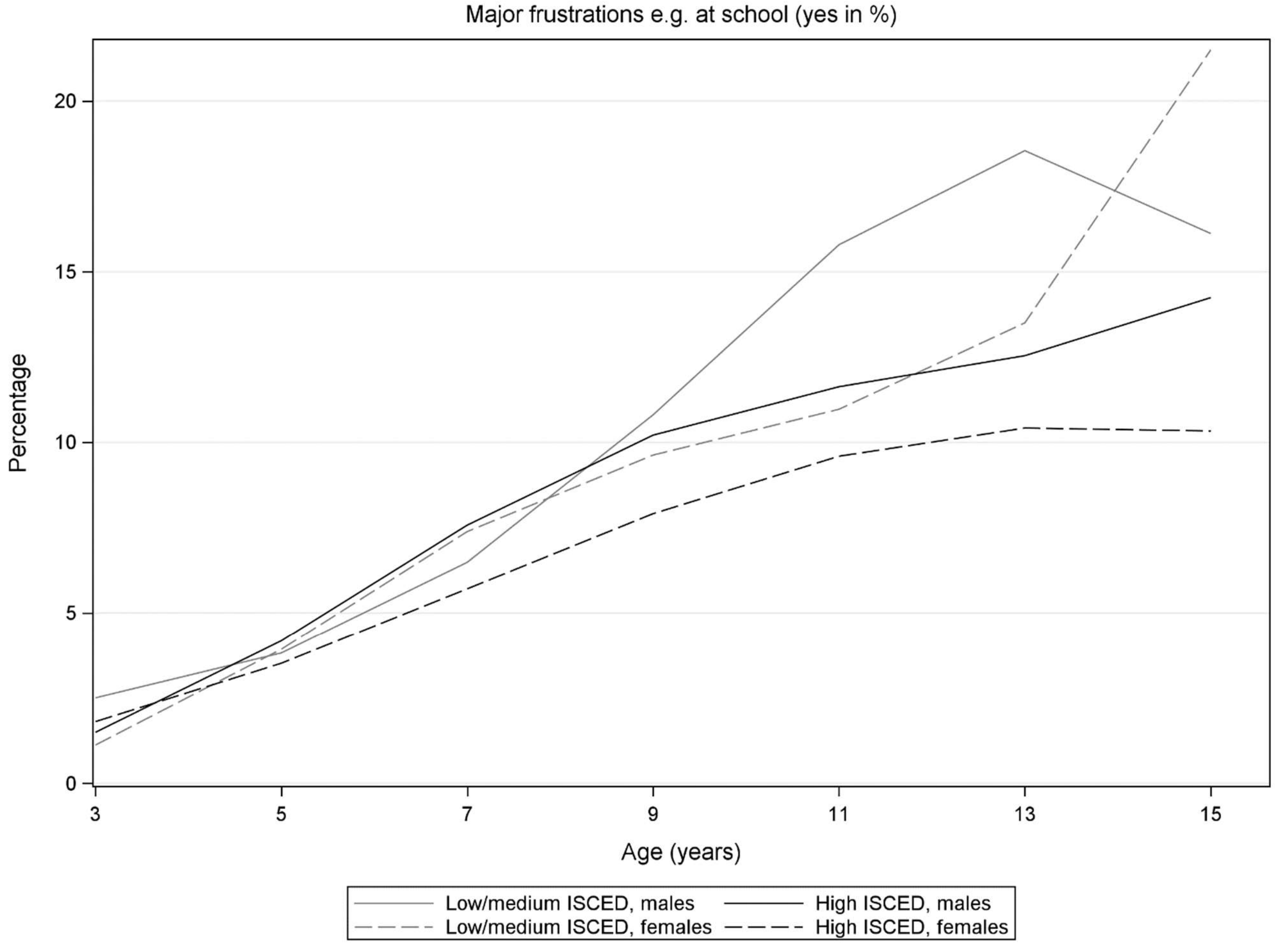

		Major frustrations (yes in %)					
		Low/medium ISCED			High ISCED		
**Sex**	**Age**	**Mean**	**LCL**	**UCL**	**Mean**	**LCL**	**UCL**
Male	3	0.03	0.01	0.04	0.01	0.01	0.03
	5	0.04	0.03	0.05	0.04	0.03	0.05
	7	0.06	0.06	0.07	0.08	0.07	0.09
	9	0.11	0.10	0.12	0.10	0.09	0.11
	11	0.16	0.14	0.18	0.12	0.10	0.13
	13	0.19	0.16	0.21	0.13	0.11	0.15
	15	0.16	0.10	0.24	0.14	0.09	0.22
Female	3	0.01	0.01	0.02	0.02	0.01	0.03
	5	0.04	0.03	0.05	0.04	0.03	0.04
	7	0.07	0.06	0.09	0.06	0.05	0.07
	9	0.10	0.09	0.11	0.08	0.07	0.09
	11	0.11	0.09	0.13	0.10	0.08	0.11
	13	0.14	0.11	0.16	0.10	0.09	0.13
	15	0.22	0.14	0.32	0.10	0.06	0.17

Figure and Table: Marginal means of major frustrations stratified by sex and education level for the ages 3, 5, 7, 9, 11, 13 and 15 years.

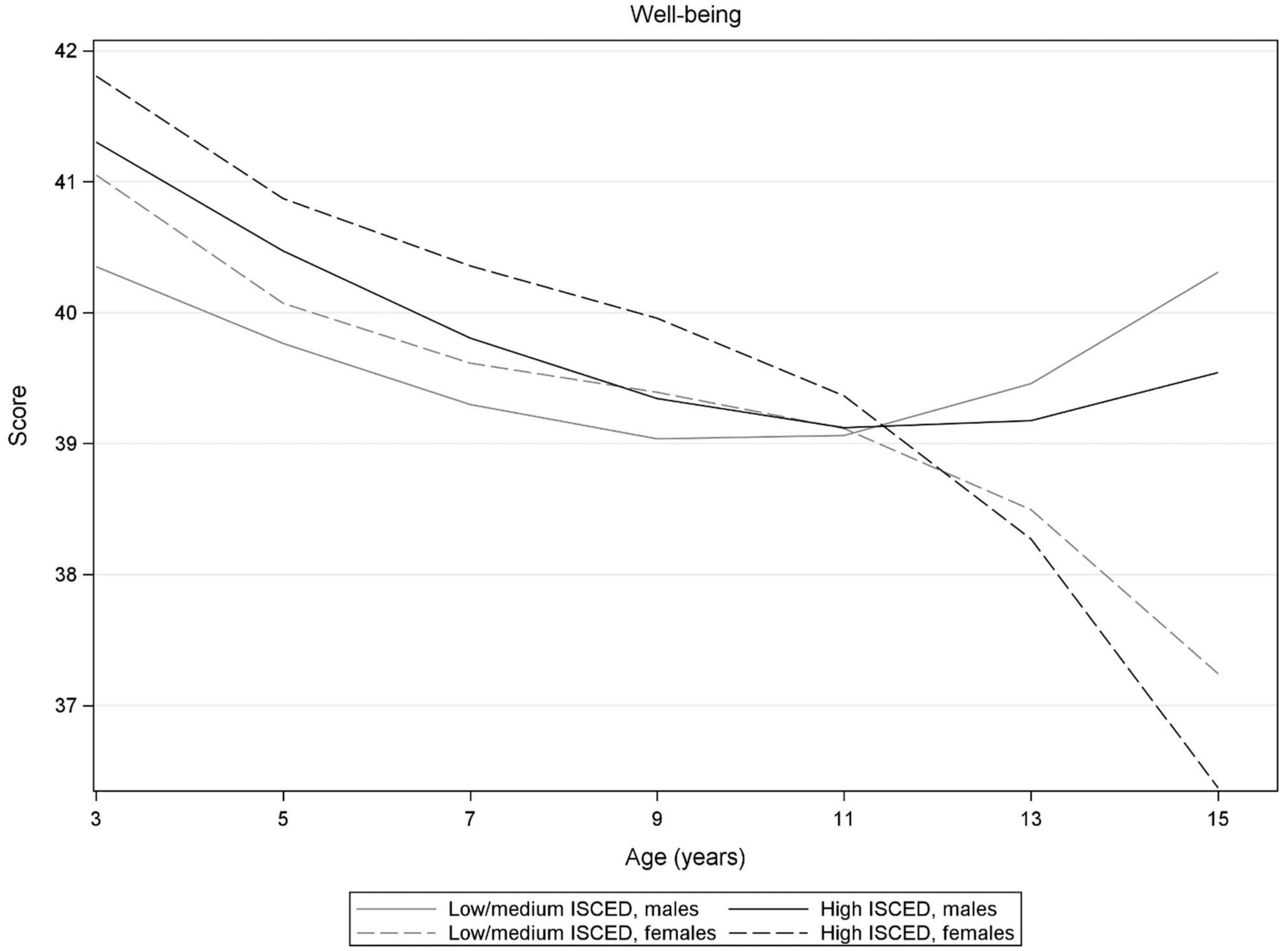

		Well-being Score					
		Low/medium ISCED			High ISCED		
Sex	Age	Mean	LCL	UCL	Mean	LCL	UCL
Male	3	40.4	39.9	40.8	41.3	40.9	41.7
	5	39.8	39.5	40.0	40.5	40.3	40.7
	7	39.3	39.1	39.5	39.8	39.6	40.0
	9	39.0	38.8	39.2	39.3	39.2	39.5
	11	39.1	38.8	39.3	39.1	38.9	39.3
	13	39.5	39.1	39.8	39.2	38.9	39.5
	15	40.3	39.5	41.1	39.5	38.8	40.3
Female	3	41.1	40.5	41.6	41.8	41.4	42.2
	5	40.1	39.8	40.3	40.9	40.7	41.1
	7	39.6	39.4	39.8	40.4	40.2	40.5
	9	39.4	39.2	39.6	40.0	39.8	40.1
	11	39.1	38.9	39.4	39.4	39.1	39.6
	13	38.5	38.2	38.8	38.3	38.0	38.6
	15	37.2	36.4	38.0	36.4	35.6	37.1

Figure and Table: Marginal means of well-being score stratified by sex and education level for the ages 3, 5, 7, 9, 11, 13 and 15 years.

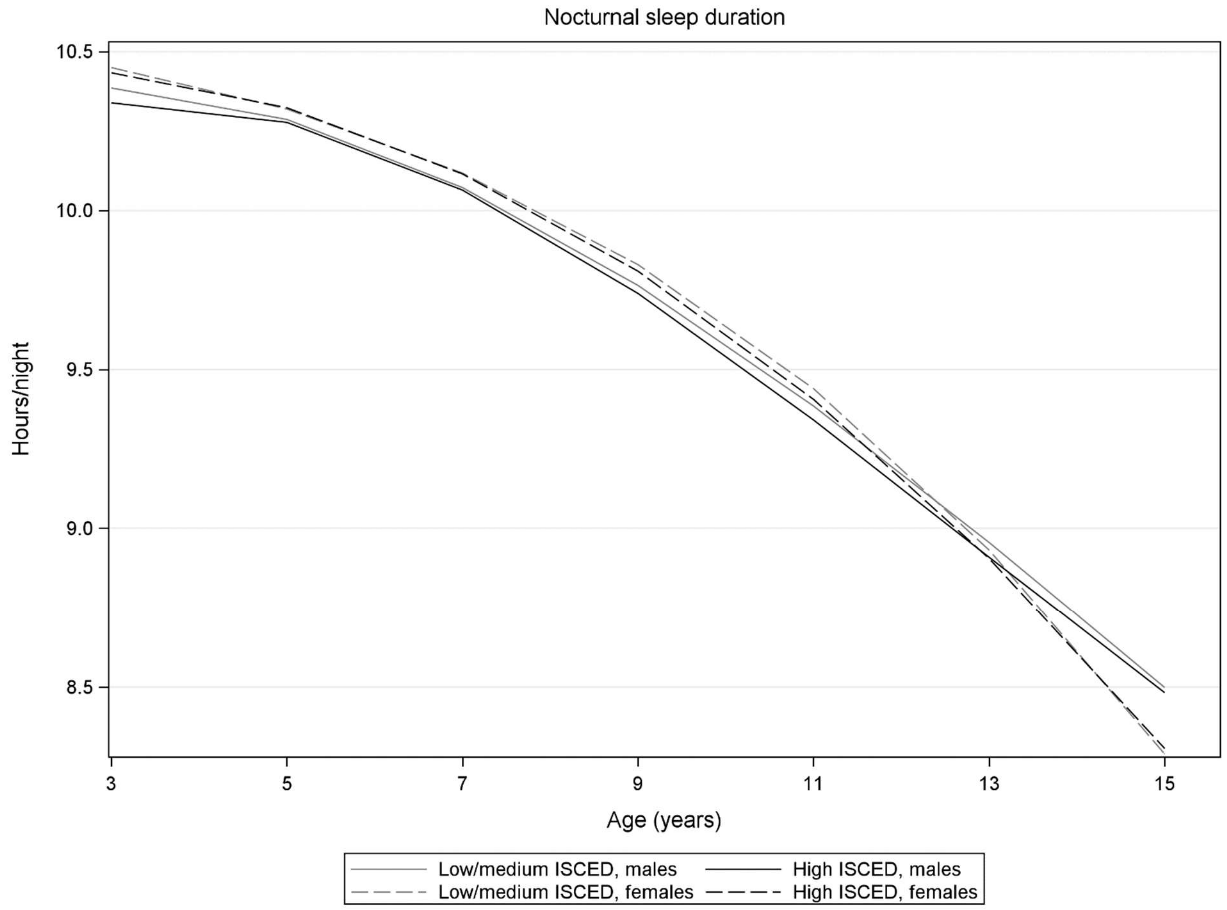

		Nocturnal sleep duration (hours/night)					
		Low/medium ISCED			High ISCED		
**Sex**	**Age**	**Mean**	**LCL**	**UCL**	**Mean**	**LCL**	**UCL**
Male	3	10.4	10.3	10.5	10.3	10.3	10.4
	5	10.3	10.2	10.3	10.3	10.2	10.3
	7	10.1	10.0	10.1	10.1	10.0	10.1
	9	9.8	9.7	9.8	9.7	9.7	9.8
	11	9.4	9.3	9.4	9.3	9.3	9.4
	13	9.0	8.9	9.0	8.9	8.9	9.0
	15	8.5	8.3	8.7	8.5	8.4	8.6
Female	3	10.4	10.4	10.5	10.4	10.4	10.5
	5	10.3	10.3	10.4	10.3	10.3	10.4
	7	10.1	10.1	10.1	10.1	10.1	10.1
	9	9.8	9.8	9.9	9.8	9.8	9.8
	11	9.4	9.4	9.5	9.4	9.4	9.4
	13	8.9	8.9	9.0	8.9	8.9	9.0
	15	8.3	8.1	8.4	8.3	8.2	8.4

Figure and Table: Marginal means of nocturnal sleep duration stratified by sex and education level for the ages 3, 5, 7, 9, 11, 13 and 15 years.

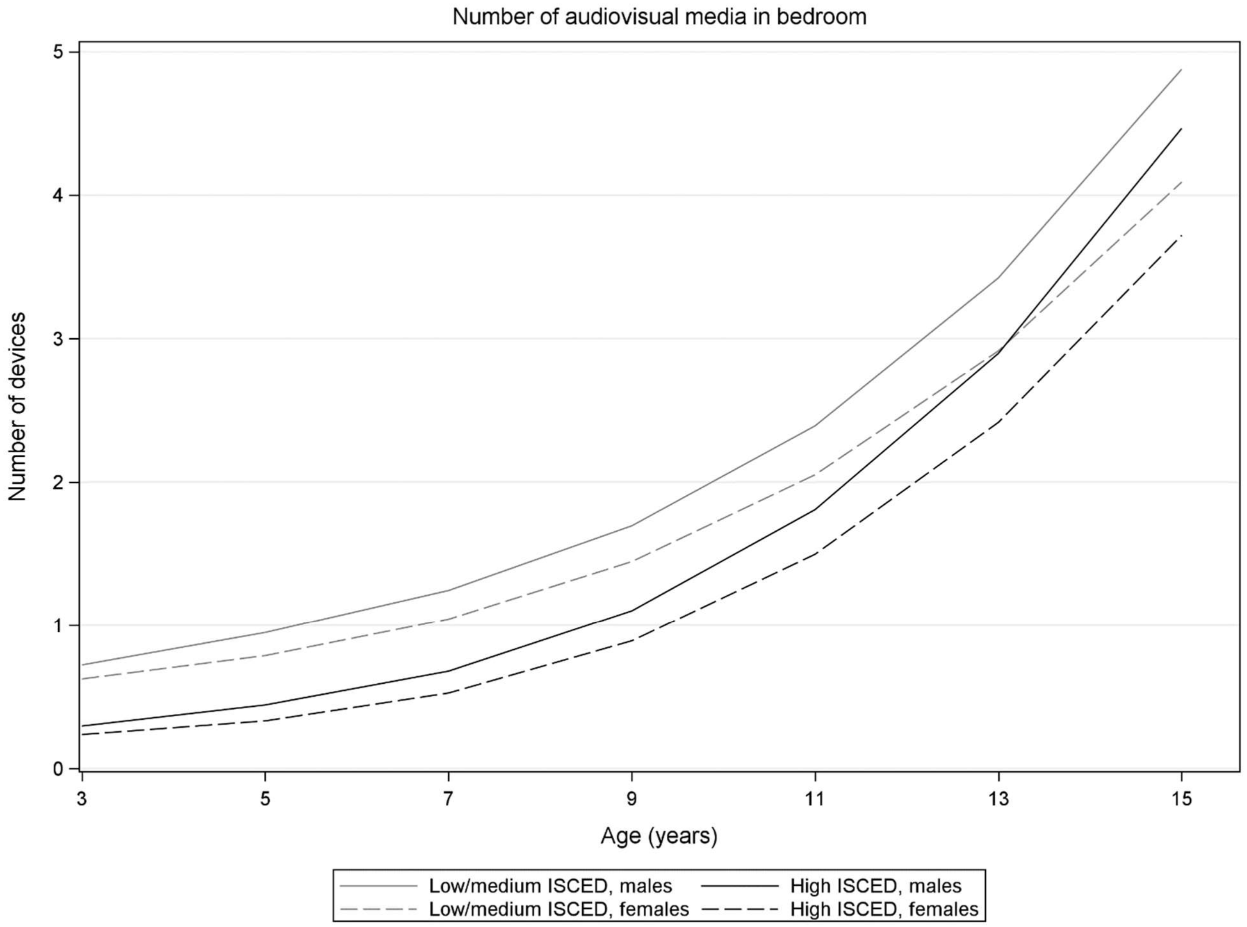

		Number of media in bedroom					
		Low/medium ISCED			High ISCED		
Sex	Age	Mean	LCL	UCL	Mean	LCL	UCL
Male	3	0.7	0.6	0.8	0.3	0.2	0.4
	5	0.9	0.9	1.0	0.4	0.4	0.5
	7	1.2	1.2	1.3	0.7	0.6	0.7
	9	1.7	1.6	1.8	1.1	1.1	1.2
	11	2.4	2.3	2.5	1.8	1.7	1.9
	13	3.4	3.3	3.5	2.9	2.8	3.0
	15	4.9	4.6	5.1	4.5	4.3	4.7
Female	3	0.6	0.5	0.8	0.2	0.2	0.3
	5	0.8	0.7	0.8	0.3	0.3	0.4
	7	1.0	1.0	1.1	0.5	0.5	0.6
	9	1.4	1.4	1.5	0.9	0.8	0.9
	11	2.1	2.0	2.1	1.5	1.4	1.6
	13	2.9	2.8	3.0	2.4	2.3	2.5
	15	4.1	3.9	4.3	3.7	3.5	3.9

Figure and Table: Marginal means of number of media in bedroom stratified by sex and education level for the ages 3, 5, 7, 9, 11, 13 and 15 years.

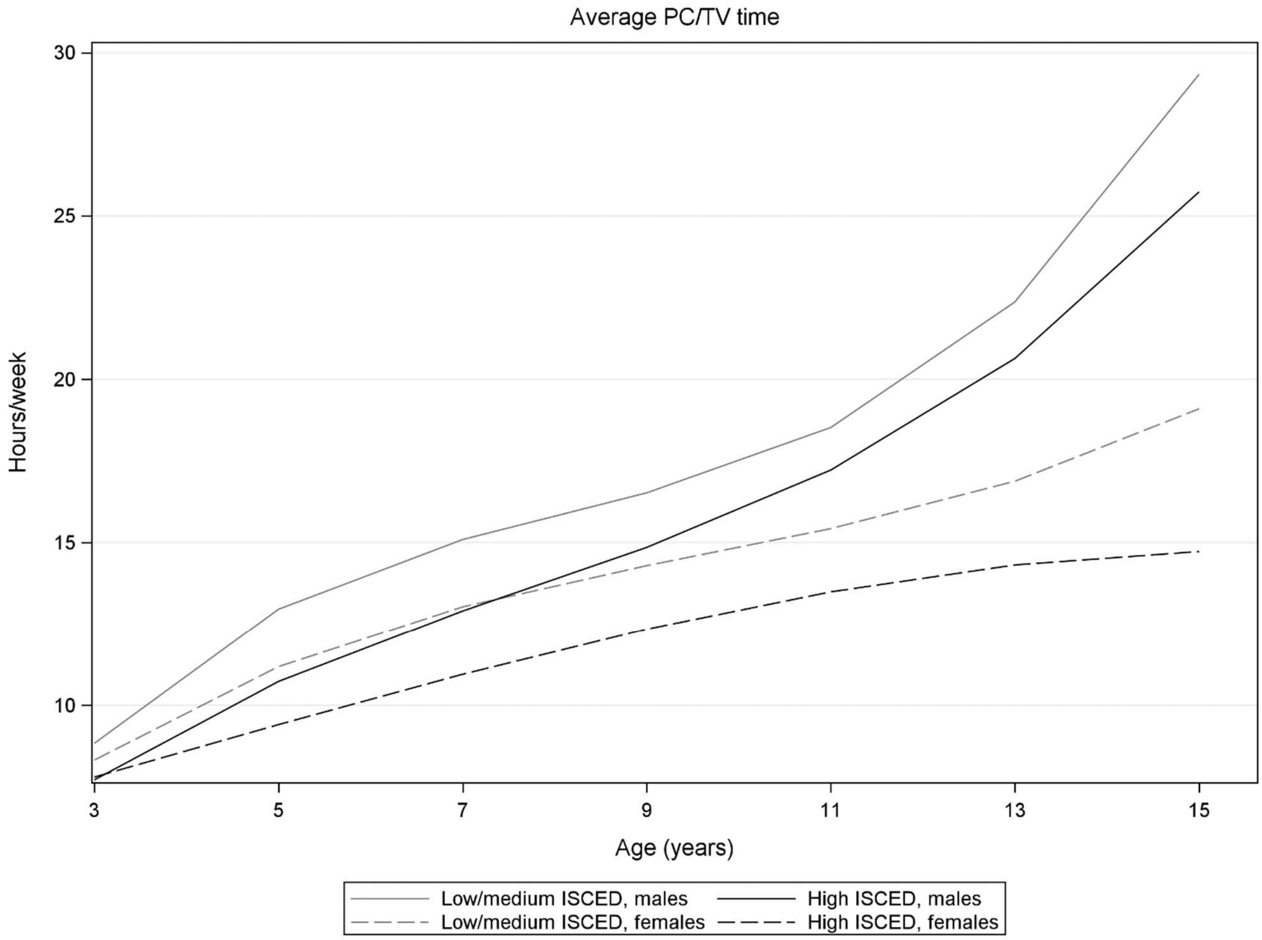

Sex	Age	Average PC/TV time (hours/week) Low/medium ISCED Mean	LCL	UCL	High ISCED Mean	LCL	UCL
Male	3	8.8	8.1	9.6	7.7	7.2	8.3
	5	13.0	12.6	13.3	10.7	10.5	11.0
	7	15.1	14.8	15.4	12.9	12.7	13.1
	9	16.5	16.2	16.8	14.8	14.6	15.1
	11	18.5	18.1	19.0	17.2	16.8	17.6
	13	22.4	21.7	23.0	20.6	20.1	21.2
	15	29.3	27.8	30.8	25.7	24.6	26.9
Female	3	8.3	7.5	9.2	7.8	7.3	8.4
	5	11.2	10.8	11.6	9.4	9.1	9.7
	7	13.0	12.7	13.3	10.9	10.7	11.2
	9	14.3	14.0	14.6	12.3	12.1	12.6
	11	15.4	15.0	15.9	13.5	13.1	13.9
	13	16.9	16.2	17.5	14.3	13.8	14.9
	15	19.1	17.6	20.6	14.7	13.6	15.9

Figure and Table: Marginal means of average TV/PC time stratified by sex and education level for the ages 3, 5, 7, 9, 11, 13 and 15 years.

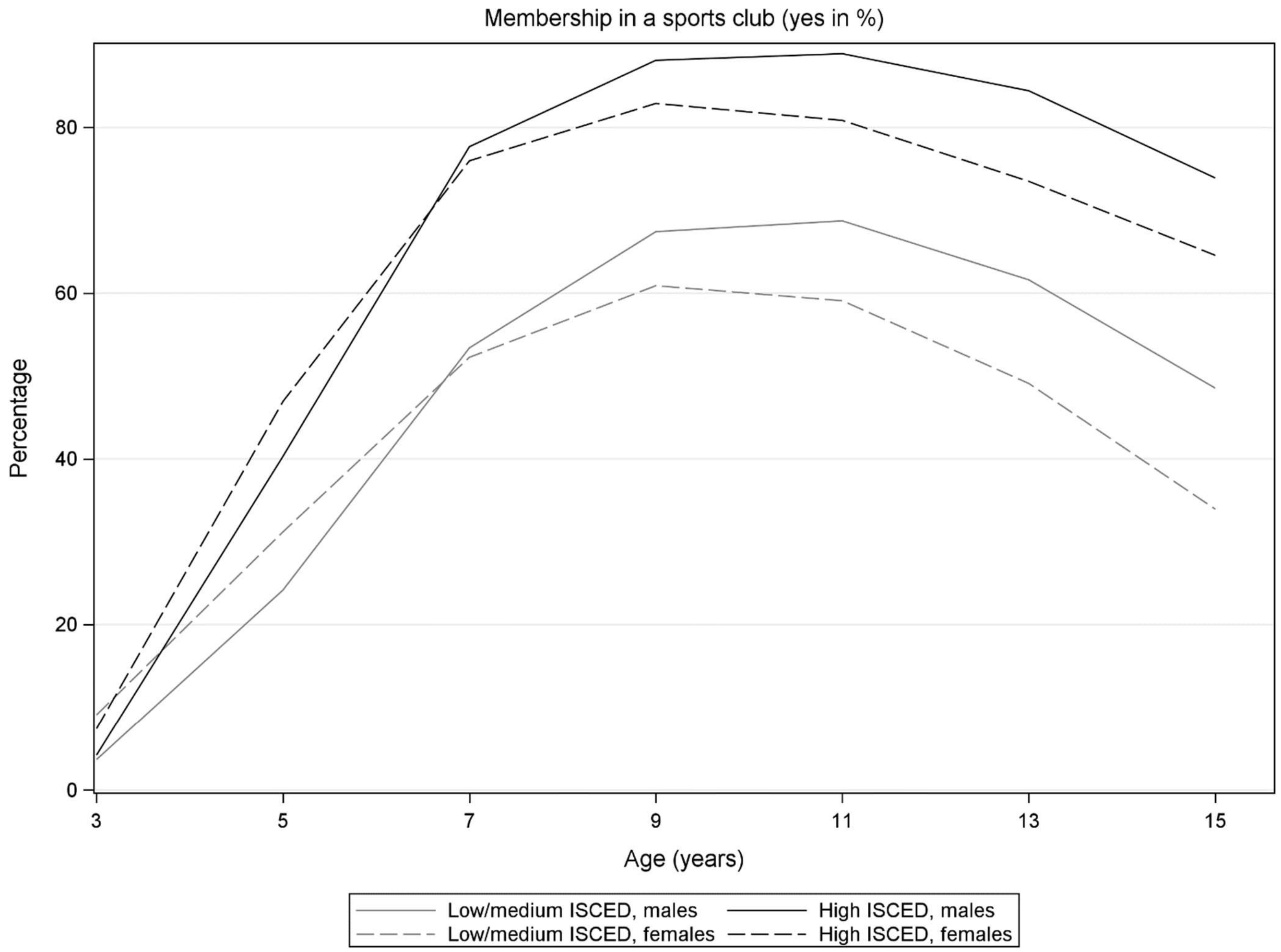

		Membership in a sports club (yes in %)					
		Low/medium ISCED			High ISCED		
**Sex**	**Age**	**Mean**	**LCL**	**UCL**	**Mean**	**LCL**	**UCL**
Male	3	0.04	0.03	0.05	0.04	0.03	0.05
	5	0.24	0.22	0.26	0.40	0.38	0.43
	7	0.53	0.51	0.56	0.78	0.76	0.79
	9	0.67	0.65	0.70	0.88	0.87	0.89
	11	0.69	0.66	0.71	0.89	0.87	0.90
	13	0.62	0.58	0.65	0.84	0.82	0.87
	15	0.49	0.39	0.58	0.74	0.66	0.81
Female	3	0.09	0.07	0.12	0.07	0.06	0.09
	5	0.31	0.29	0.34	0.47	0.44	0.50
	7	0.52	0.50	0.55	0.76	0.74	0.78
	9	0.61	0.59	0.63	0.83	0.81	0.84
	11	0.59	0.56	0.62	0.81	0.79	0.83
	13	0.49	0.45	0.53	0.73	0.70	0.77
	15	0.34	0.26	0.43	0.65	0.55	0.73

Figure and Table: Marginal means of membership in sports club stratified by sex and education level for the ages 3, 5, 7, 9, 11, 13 and 15 years.

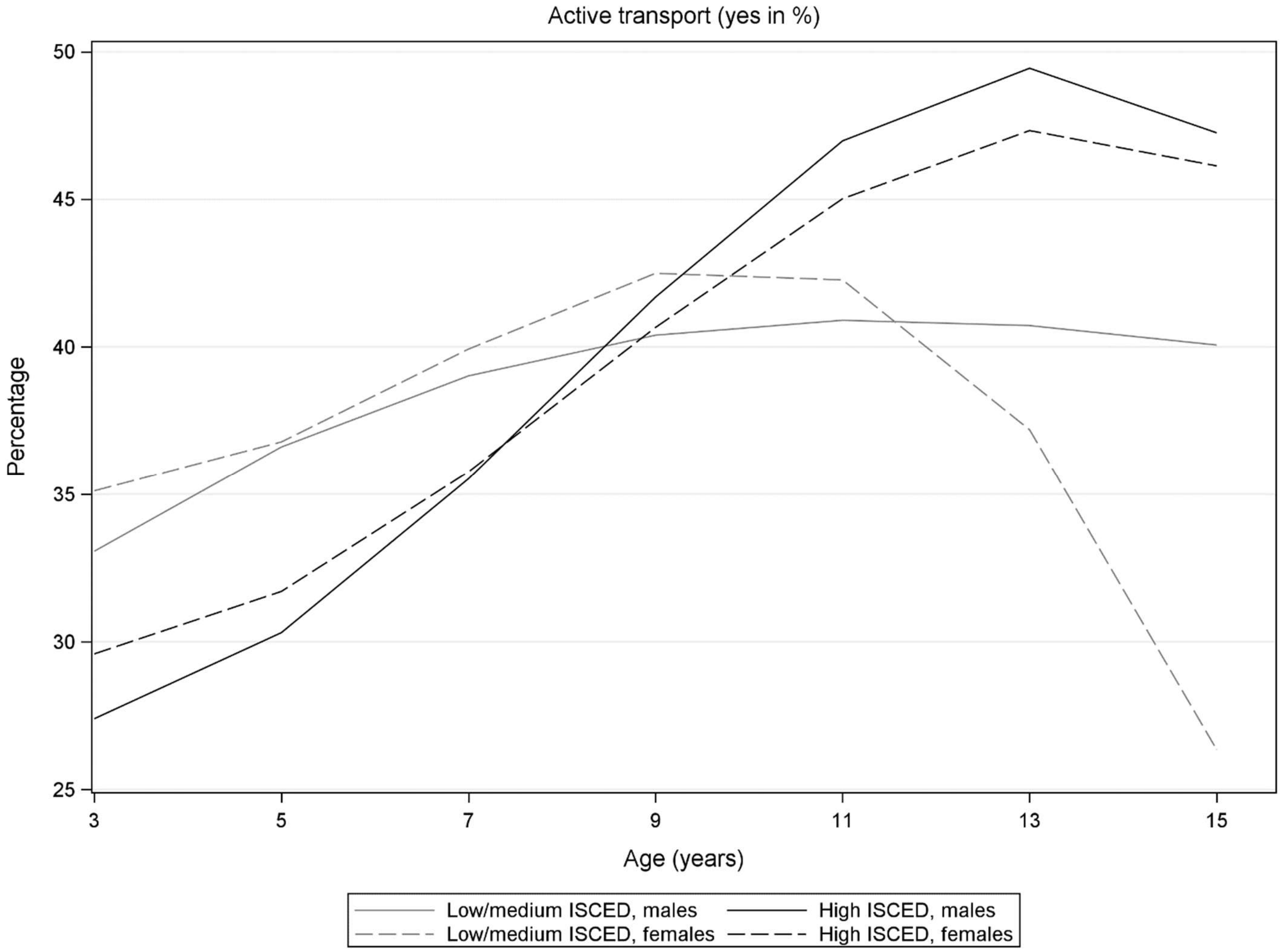

		Active transport (yes in %)					
		Low/medium ISCED			High ISCED		
**Sex**	**Age**	**Mean**	**LCL**	**UCL**	**Mean**	**LCL**	**UCL**
Male	3	0.33	0.29	0.38	0.27	0.24	0.31
	5	0.37	0.35	0.39	0.30	0.29	0.32
	7	0.39	0.37	0.41	0.36	0.34	0.37
	9	0.40	0.39	0.42	0.42	0.40	0.43
	11	0.41	0.38	0.43	0.47	0.44	0.50
	13	0.41	0.37	0.44	0.49	0.46	0.53
	15	0.40	0.31	0.49	0.47	0.38	0.56
Female	3	0.35	0.30	0.40	0.30	0.26	0.34
	5	0.37	0.35	0.39	0.32	0.30	0.34
	7	0.40	0.38	0.42	0.36	0.34	0.38
	9	0.42	0.41	0.44	0.41	0.39	0.42
	11	0.42	0.40	0.45	0.45	0.42	0.48
	13	0.37	0.34	0.41	0.47	0.44	0.51
	15	0.26	0.20	0.35	0.46	0.37	0.55

Figure and Table: Marginal means of active transport stratified by sex and education level for the ages 3, 5, 7, 9, 11, 13 and 15 years.

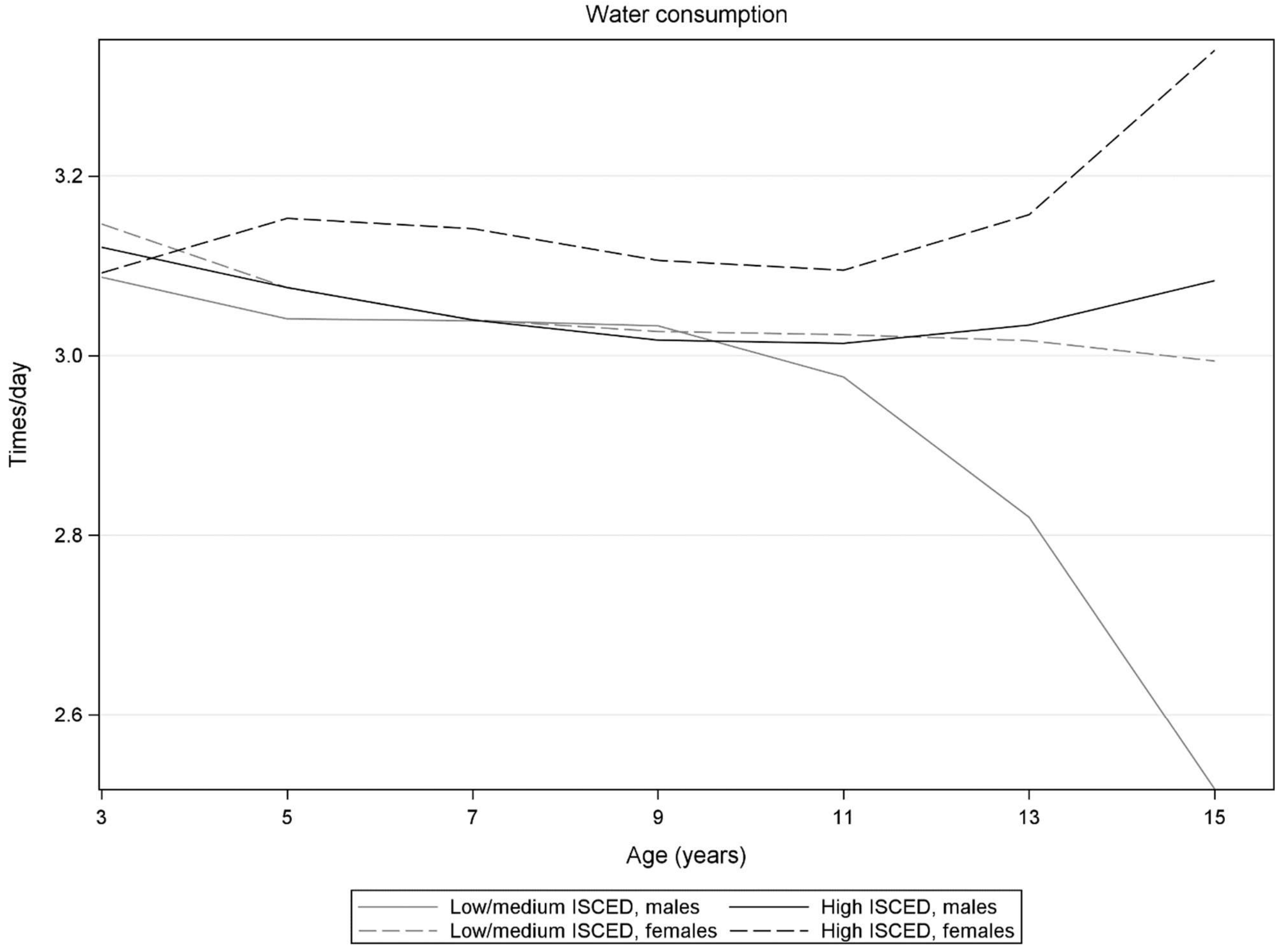

		Water consumption (times/day)					
		Low/medium ISCED			High ISCED		
**Sex**	**Age**	**Mean**	**LCL**	**UCL**	**Mean**	**LCL**	**UCL**
Male	3	3.1	3.0	3.2	3.1	3.0	3.2
	5	3.0	3.0	3.1	3.1	3.0	3.1
	7	3.0	3.0	3.1	3.0	3.0	3.1
	9	3.0	3.0	3.1	3.0	3.0	3.1
	11	3.0	2.9	3.0	3.0	3.0	3.1
	13	2.8	2.7	2.9	3.0	2.9	3.1
	15	2.5	2.3	2.7	3.1	2.9	3.3
Female	3	3.1	3.0	3.3	3.1	3.0	3.2
	5	3.1	3.0	3.1	3.2	3.1	3.2
	7	3.0	3.0	3.1	3.1	3.1	3.2
	9	3.0	3.0	3.1	3.1	3.1	3.2
	11	3.0	3.0	3.1	3.1	3.0	3.2
	13	3.0	2.9	3.1	3.2	3.1	3.2
	15	3.0	2.8	3.2	3.3	3.1	3.5

Figure and Table: Marginal means of water consumption stratified by sex and education level for the ages 3, 5, 7, 9, 11, 13 and 15 years.

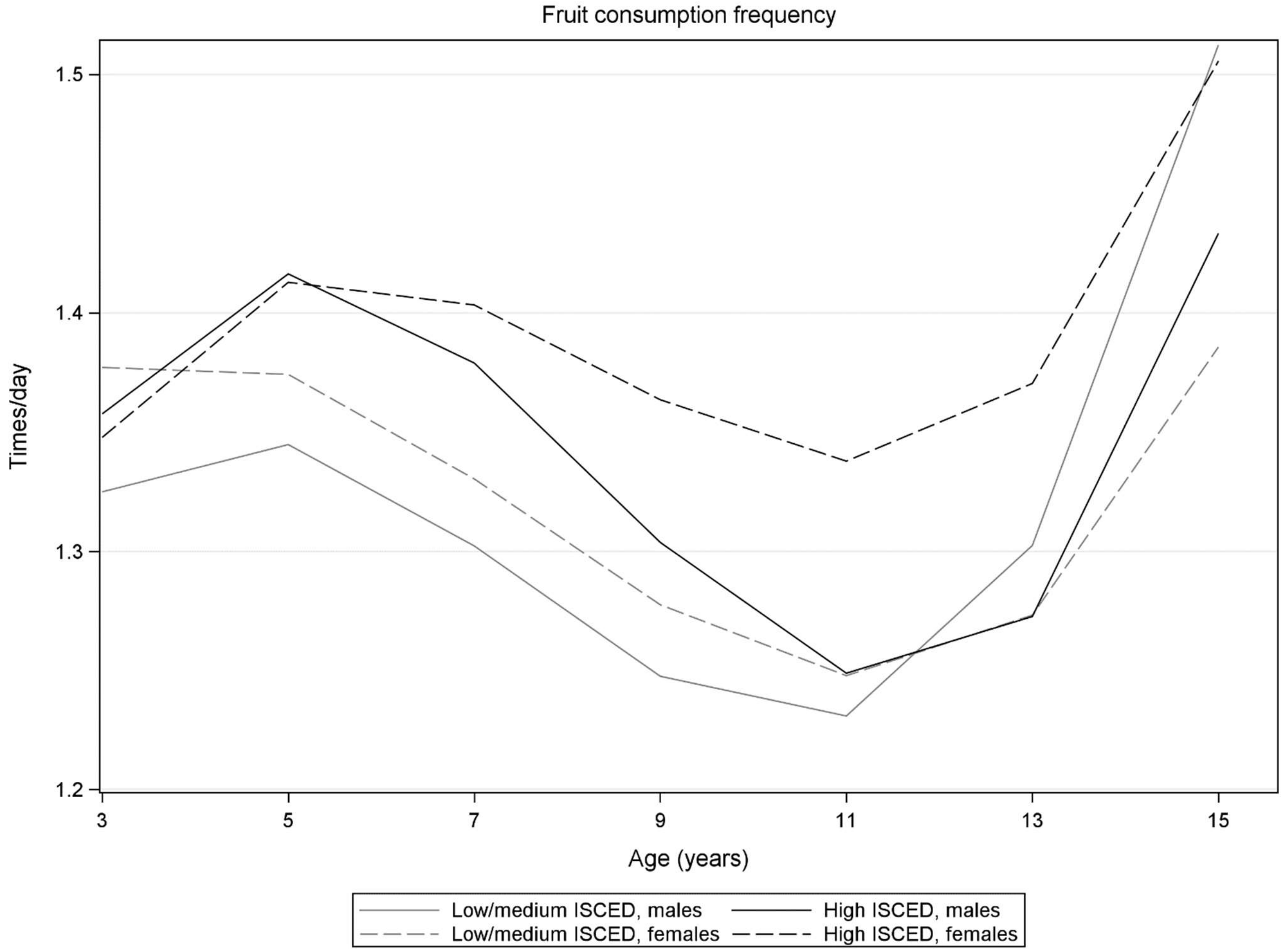

		Fruits consumption frequency (times/day)					
		Low/medium ISCED			High ISCED		
Sex	Age	Mean	LCL	UCL	Mean	LCL	UCL
Male	3	1.3	1.2	1.4	1.4	1.3	1.4
	5	1.3	1.3	1.4	1.4	1.4	1.5
	7	1.3	1.3	1.3	1.4	1.3	1.4
	9	1.2	1.2	1.3	1.3	1.3	1.3
	11	1.2	1.2	1.3	1.2	1.2	1.3
	13	1.3	1.2	1.4	1.3	1.2	1.3
	15	1.5	1.3	1.7	1.4	1.3	1.6
Female	3	1.4	1.2	1.5	1.3	1.3	1.4
	5	1.4	1.3	1.4	1.4	1.4	1.5
	7	1.3	1.3	1.4	1.4	1.4	1.4
	9	1.3	1.2	1.3	1.4	1.3	1.4
	11	1.2	1.2	1.3	1.3	1.3	1.4
	13	1.3	1.2	1.4	1.4	1.3	1.4
	15	1.4	1.2	1.6	1.5	1.3	1.7

Figure and Table: Marginal means of fruit consumption stratified by sex and education level for the ages 3, 5, 7, 9, 11, 13 and 15 years.

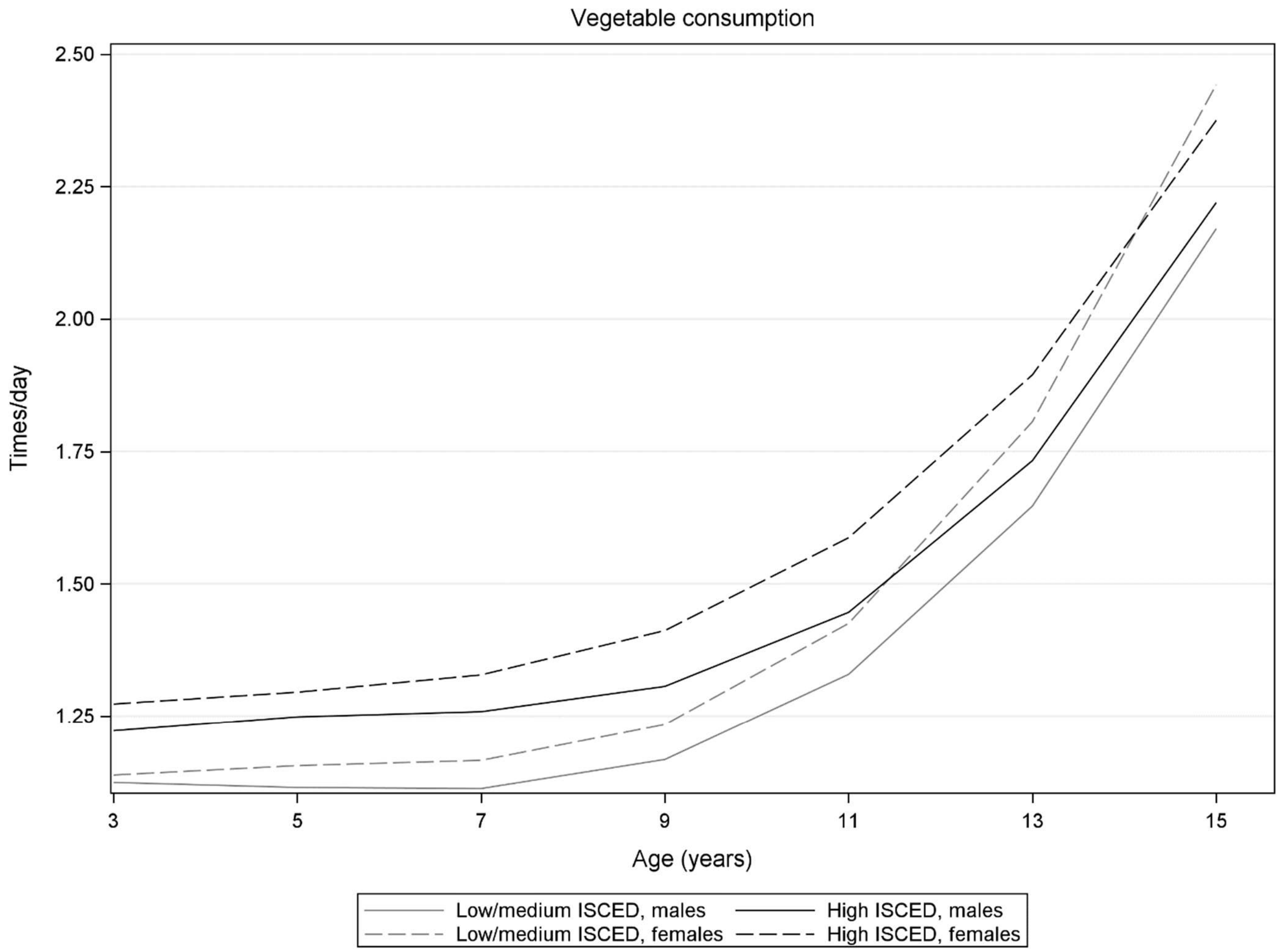

		Vegetable consumption (times/day)					
		Low/medium ISCED			High ISCED		
Sex	Age	Mean	LCL	UCL	Mean	LCL	UCL
Male	3	1.1	1.0	1.2	1.2	1.1	1.3
	5	1.1	1.1	1.2	1.2	1.2	1.3
	7	1.1	1.1	1.1	1.3	1.2	1.3
	9	1.2	1.1	1.2	1.3	1.3	1.3
	11	1.3	1.3	1.4	1.4	1.4	1.5
	13	1.6	1.5	1.7	1.7	1.7	1.8
	15	2.2	1.9	2.4	2.2	2.0	2.4
Female	3	1.1	1.0	1.3	1.3	1.2	1.4
	5	1.2	1.1	1.2	1.3	1.3	1.3
	7	1.2	1.1	1.2	1.3	1.3	1.4
	9	1.2	1.2	1.3	1.4	1.4	1.4
	11	1.4	1.4	1.5	1.6	1.5	1.6
	13	1.8	1.7	1.9	1.9	1.8	2.0
	15	2.4	2.2	2.7	2.4	2.2	2.6

Figure and Table: Marginal means of vegetable consumption stratified by sex and education level for the ages 3, 5, 7, 9, 11, 13 and 15 years.

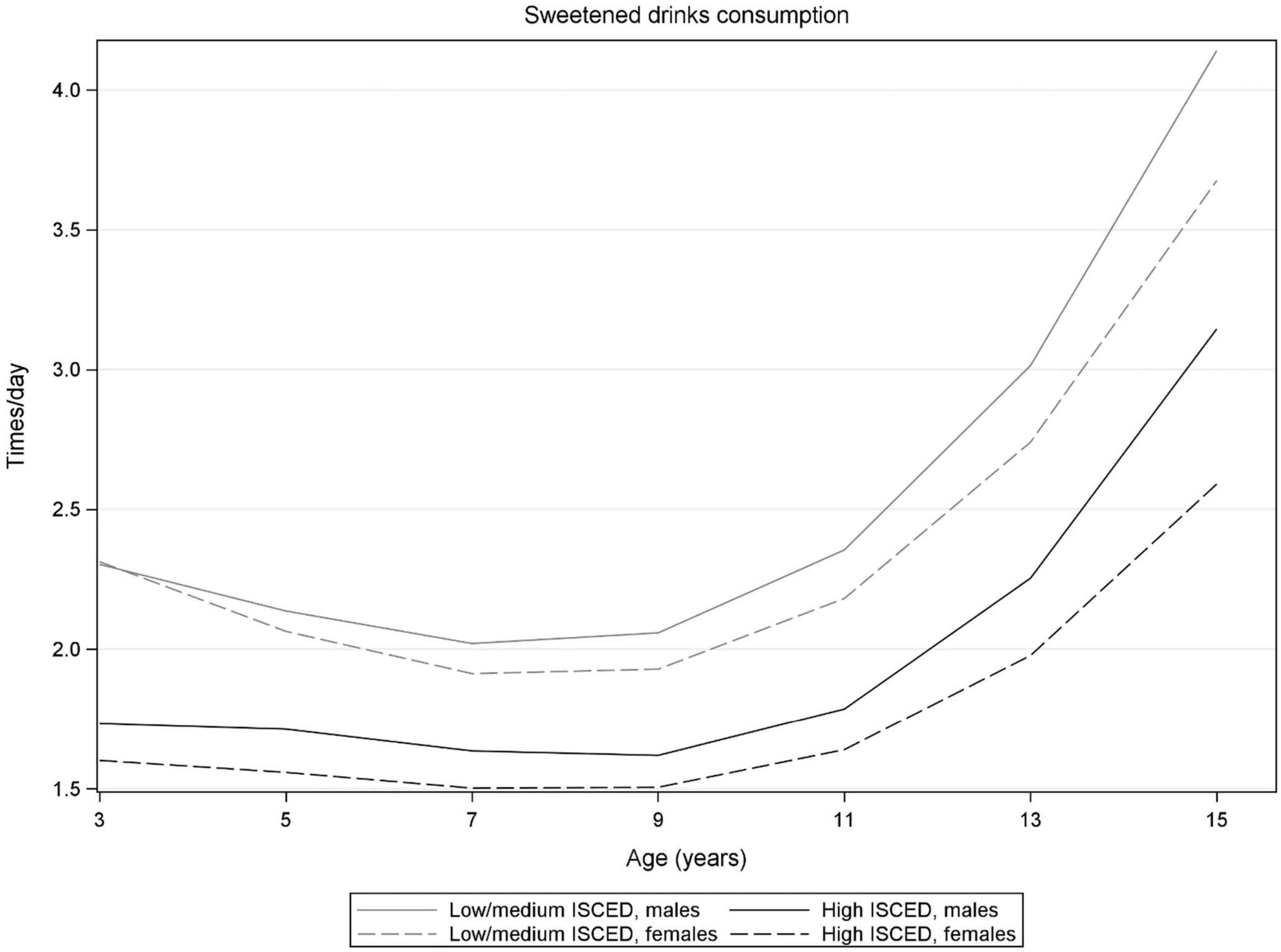

Sex	Age	Sweetened drinks (times/day) Low/medium ISCED Mean	LCL	UCL	High ISCED Mean	LCL	UCL
Male	3	2.3	2.1	2.5	1.7	1.6	1.9
	5	2.1	2.0	2.2	1.7	1.6	1.8
	7	2.0	1.9	2.1	1.6	1.6	1.7
	9	2.1	2.0	2.1	1.6	1.6	1.7
	11	2.4	2.2	2.5	1.8	1.7	1.9
	13	3.0	2.8	3.2	2.3	2.1	2.4
	15	4.1	3.7	4.6	3.1	2.9	3.4
Female	3	2.3	2.1	2.5	1.6	1.5	1.7
	5	2.1	2.0	2.2	1.6	1.5	1.6
	7	1.9	1.8	2.0	1.5	1.4	1.6
	9	1.9	1.8	2.0	1.5	1.5	1.6
	11	2.2	2.1	2.3	1.6	1.6	1.7
	13	2.7	2.6	2.9	2.0	1.9	2.1
	15	3.7	3.3	4.1	2.6	2.3	2.8

Figure and Table: Marginal means of sweetened drinks consumption stratified by sex and education level for the ages 3, 5, 7, 9, 11, 13 and 15 years.

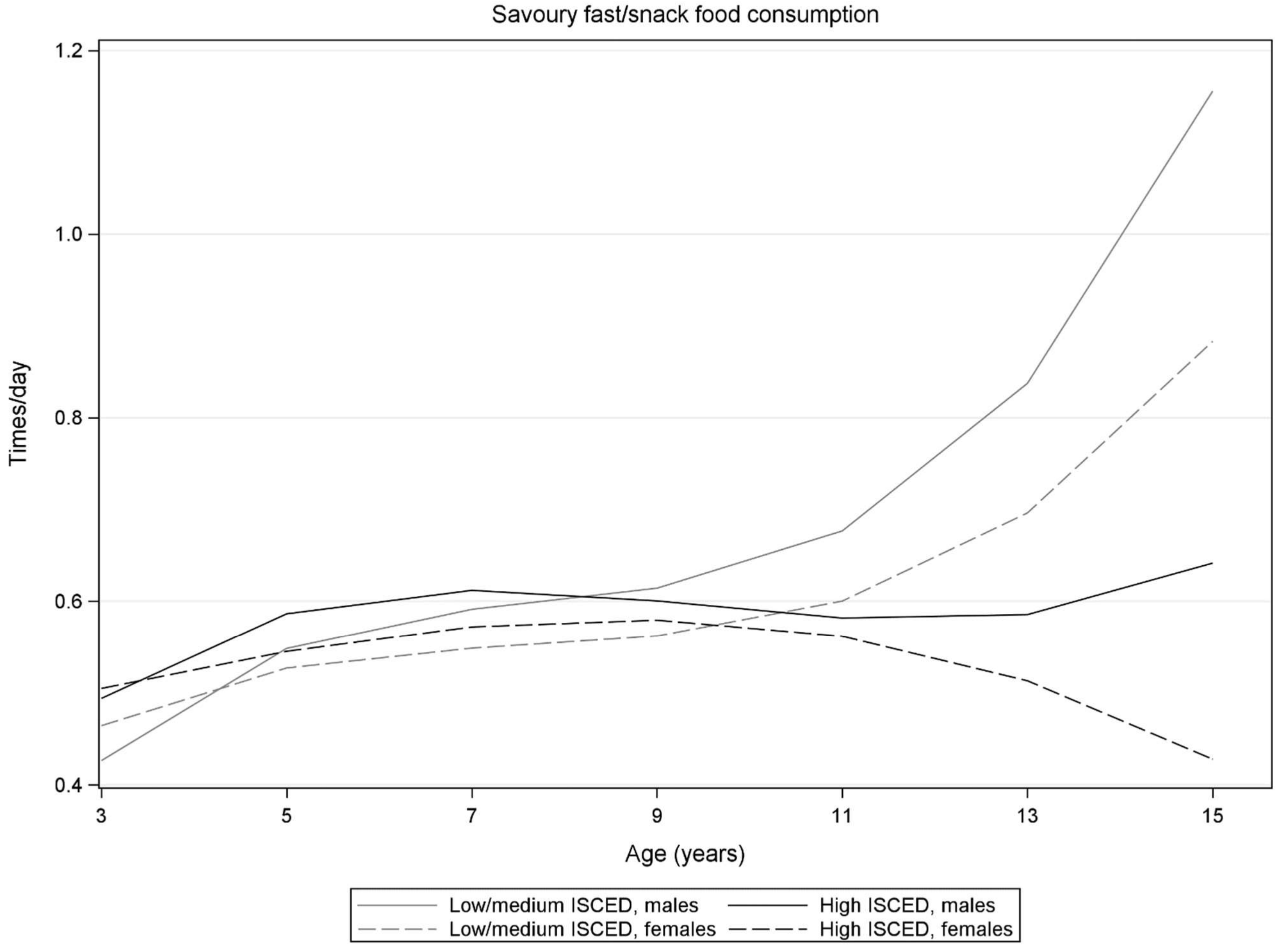

		Savoury fast and snack food consumption (times/day)					
		Low/medium ISCED			High ISCED		
**Sex**	**Age**	**Mean**	**LCL**	**UCL**	**Mean**	**LCL**	**UCL**
Male	3	0.4	0.4	0.5	0.5	0.4	0.5
	5	0.5	0.5	0.6	0.6	0.6	0.6
	7	0.6	0.6	0.6	0.6	0.6	0.6
	9	0.6	0.6	0.6	0.6	0.6	0.6
	11	0.7	0.6	0.7	0.6	0.6	0.6
	13	0.8	0.8	0.9	0.6	0.5	0.6
	15	1.2	1.0	1.3	0.6	0.5	0.7
Female	3	0.5	0.4	0.5	0.5	0.5	0.6
	5	0.5	0.5	0.6	0.5	0.5	0.6
	7	0.5	0.5	0.6	0.6	0.6	0.6
	9	0.6	0.5	0.6	0.6	0.6	0.6
	11	0.6	0.6	0.6	0.6	0.5	0.6
	13	0.7	0.6	0.8	0.5	0.5	0.6
	15	0.9	0.7	1.0	0.4	0.3	0.5

Figure and Table: Marginal means of savoury fast and snack food consumption stratified by sex and education level for the ages 3, 5, 7, 9, 11, 13 and 15 years.

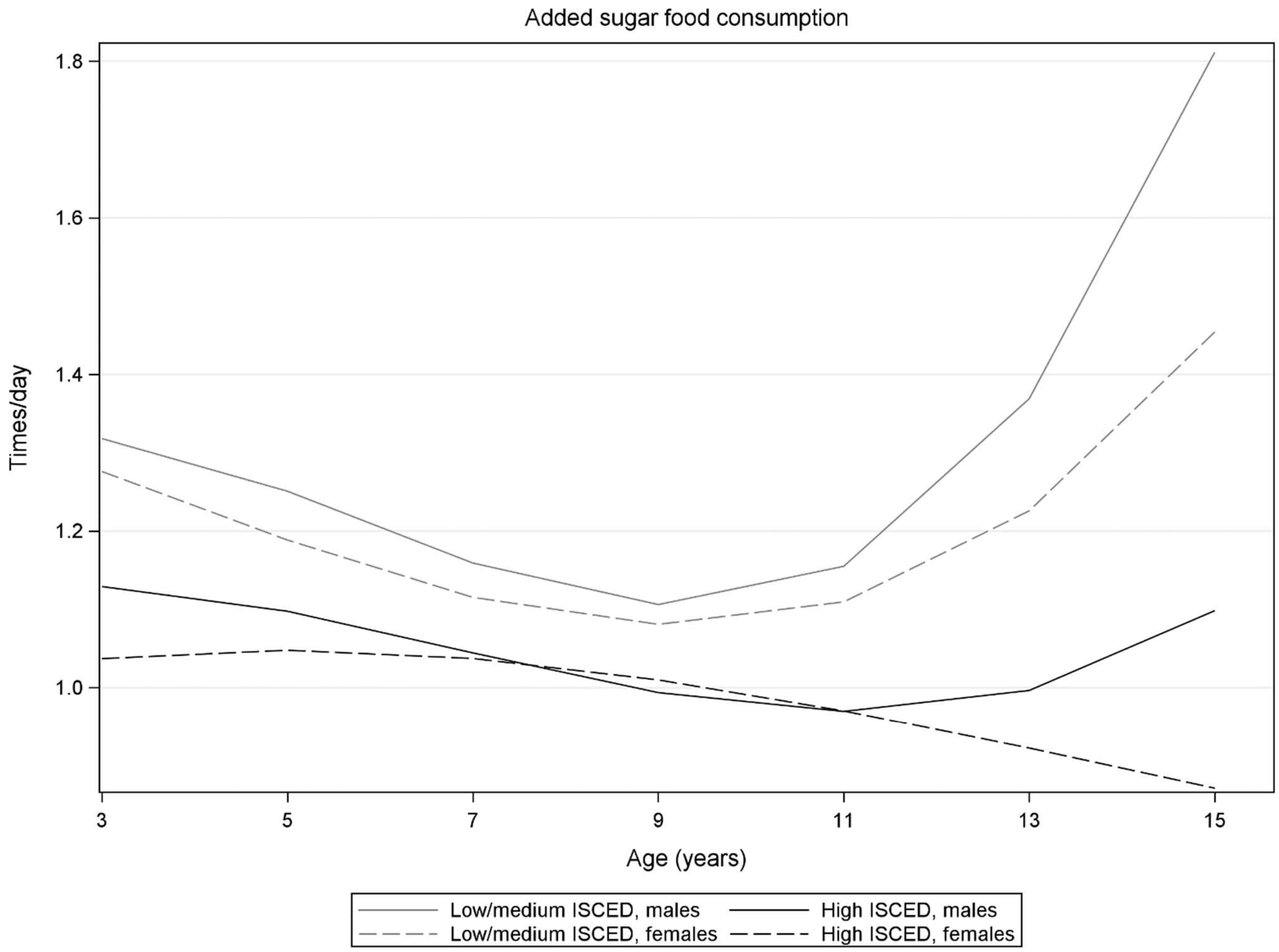

		Added sugar food consumption (times/day)					
		Low/medium ISCED			High ISCED		
Sex	Age	Mean	LCL	UCL	Mean	LCL	UCL
Male	3	1.3	1.2	1.4	1.1	1.1	1.2
	5	1.3	1.2	1.3	1.1	1.1	1.1
	7	1.2	1.1	1.2	1.0	1.0	1.1
	9	1.1	1.1	1.2	1.0	1.0	1.0
	11	1.2	1.1	1.2	1.0	0.9	1.0
	13	1.4	1.3	1.5	1.0	0.9	1.1
	15	1.8	1.6	2.0	1.1	1.0	1.2
Female	3	1.3	1.1	1.4	1.0	1.0	1.1
	5	1.2	1.1	1.2	1.0	1.0	1.1
	7	1.1	1.1	1.2	1.0	1.0	1.1
	9	1.1	1.0	1.1	1.0	1.0	1.0
	11	1.1	1.0	1.2	1.0	0.9	1.0
	13	1.2	1.1	1.3	0.9	0.9	1.0
	15	1.5	1.2	1.7	0.9	0.7	1.0

Figure and Table: Marginal means of added sugar food consumption stratified by sex and education level for the ages 3, 5, 7, 9, 11, 13 and 15 years.

Supplementary Material S7

The following figures and tables depict the marginal mean BMI values estimated in children at certain fixed exposure levels stratified by sex and parental educational level. For instance, on the following page the marginal mean BMI estimates of children with vs without migrant background are plotted against age, stratified by sex and education level. The table presents the corresponding numbers as well as lower and upper 95% confidence intervals.

LCL: lower 95% confidence interval

UCL: upper 95% confidence interval

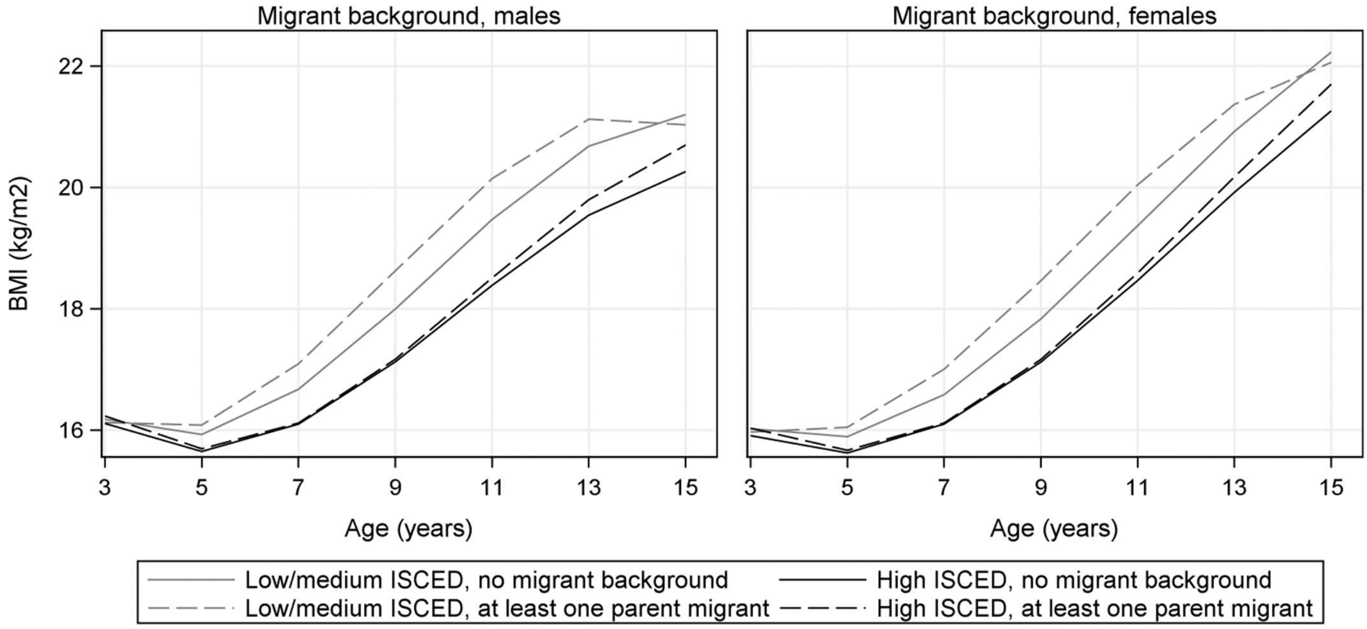

			Males			Females		
ISCED	Migrant background	Age	Estimated BMI	LCL	UCL	Estimated BMI	LCL	UCL
Low/medium	No	3	16.2	16.0	16.3	16.0	15.8	16.2
		5	15.9	15.8	16.0	15.9	15.8	16.0
		7	16.7	16.6	16.8	16.6	16.5	16.7
		9	18.0	17.9	18.1	17.8	17.7	18.0
		11	19.5	19.3	19.6	19.4	19.2	19.5
		13	20.7	20.5	20.9	20.9	20.7	21.1
		15	21.2	20.8	21.6	22.2	21.9	22.6
	At least one parent migrant	3	16.1	15.8	16.5	16.0	15.6	16.3
		5	16.1	15.9	16.3	16.0	15.8	16.2
		7	17.1	16.9	17.3	17.0	16.8	17.2
		9	18.6	18.4	18.9	18.5	18.2	18.7
		11	20.2	19.8	20.5	20.1	19.7	20.4
		13	21.1	20.7	21.6	21.4	21.0	21.8
		15	21.0	20.3	21.7	22.1	21.4	22.8
High	No	3	16.1	16.0	16.2	15.9	15.8	16.0
		5	15.6	15.6	15.7	15.6	15.5	15.7
		7	16.1	16.0	16.2	16.1	16.0	16.2
		9	17.1	17.0	17.2	17.1	17.0	17.2
		11	18.4	18.3	18.5	18.5	18.3	18.6
		13	19.5	19.4	19.7	19.9	19.8	20.1
		15	20.3	20.0	20.5	21.3	21.0	21.5
	At least one parent migrant	3	16.2	16.0	16.5	16.0	15.8	16.3
		5	15.7	15.5	15.9	15.7	15.5	15.8
		7	16.1	15.9	16.3	16.1	15.9	16.3
		9	17.2	16.9	17.4	17.2	16.9	17.4
		11	18.5	18.2	18.8	18.6	18.3	18.9
		13	19.8	19.4	20.2	20.2	19.8	20.5
		15	20.7	20.1	21.3	21.7	21.1	22.3

Figure and Table: Marginal mean BMI values comparing children with vs without parental migrant background stratified by sex and ISCED level.

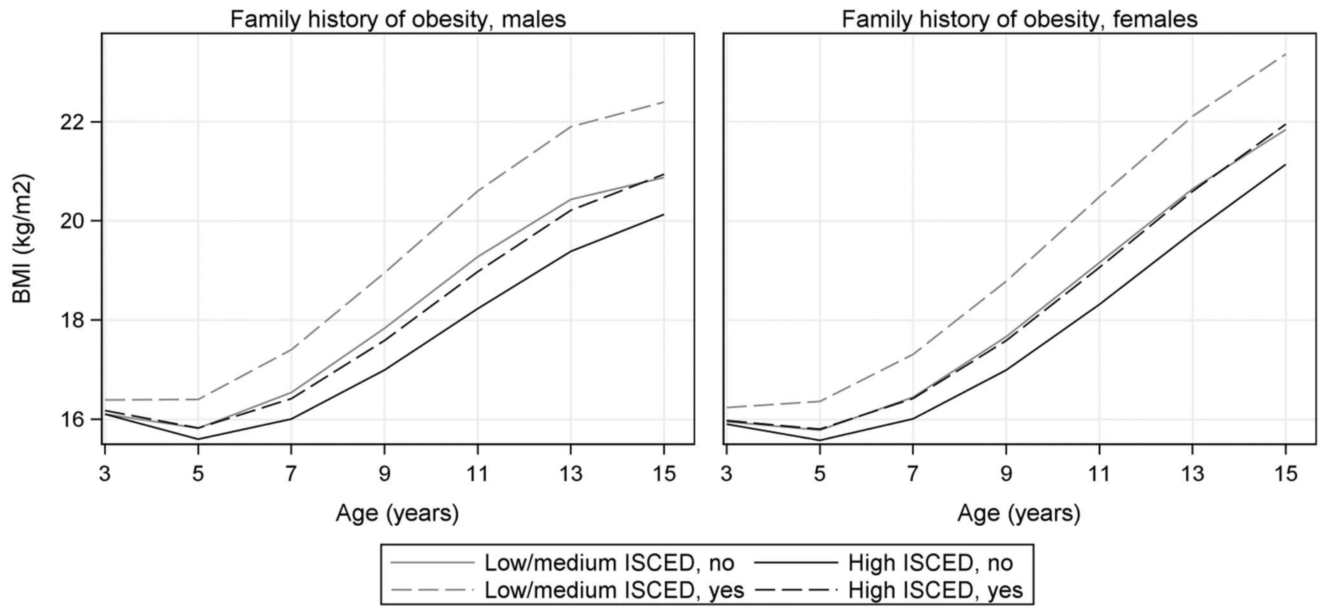

			Males			Females		
**ISCED**	**History of obesity**	**Age**	**Estimated BMI**	**LCL**	**UCL**	**Estimated BMI**	**LCL**	**UCL**
Low/medium	No	3	16.1	15.9	16.3	16.0	15.8	16.1
		5	15.8	15.7	15.9	15.8	15.7	15.9
		7	16.5	16.4	16.7	16.4	16.3	16.6
		9	17.8	17.7	18.0	17.7	17.5	17.8
		11	19.3	19.1	19.5	19.2	19.0	19.3
		13	20.4	20.2	20.7	20.6	20.4	20.9
		15	20.9	20.5	21.2	21.8	21.5	22.2
	Yes	3	16.4	16.1	16.7	16.2	15.9	16.5
		5	16.4	16.2	16.6	16.4	16.2	16.5
		7	17.4	17.2	17.6	17.3	17.1	17.5
		9	19.0	18.7	19.2	18.8	18.6	19.0
		11	20.6	20.3	20.9	20.5	20.2	20.8
		13	21.9	21.6	22.3	22.1	21.8	22.5
		15	22.4	21.8	23.0	23.4	22.8	23.9
High	No	3	16.1	16.0	16.2	15.9	15.8	16.0
		5	15.6	15.5	15.7	15.6	15.5	15.7
		7	16.0	15.9	16.1	16.0	15.9	16.1
		9	17.0	16.9	17.1	17.0	16.9	17.1
		11	18.2	18.1	18.4	18.3	18.2	18.4
		13	19.4	19.2	19.6	19.8	19.6	19.9
		15	20.1	19.8	20.4	21.1	20.8	21.4
	Yes	3	16.2	16.0	16.4	16.0	15.8	16.2
		5	15.8	15.7	16.0	15.8	15.7	15.9
		7	16.4	16.3	16.6	16.4	16.3	16.6
		9	17.6	17.4	17.8	17.6	17.4	17.8
		11	19.0	18.8	19.2	19.1	18.9	19.3
		13	20.2	20.0	20.5	20.6	20.3	20.9
		15	21.0	20.5	21.4	22.0	21.5	22.4

Figure and Table: Marginal mean BMI values comparing children with vs without family history of obesity stratified by sex and ISCED level.

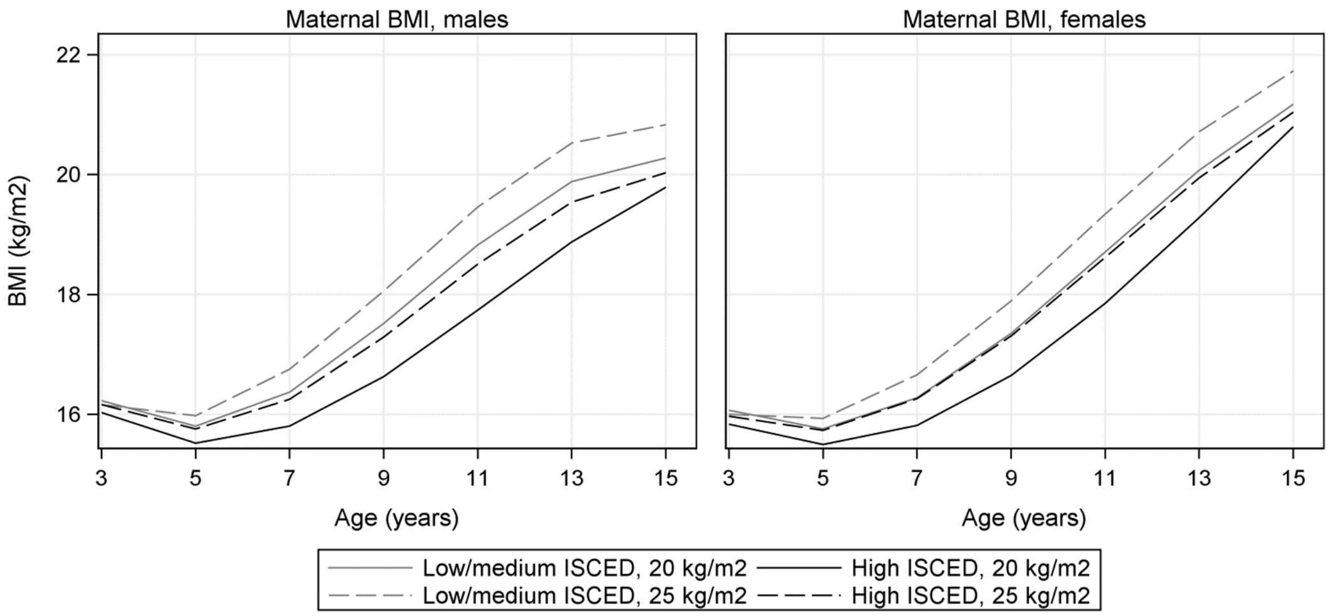

			Males			Females		
**ISCED**	**Maternal BMI**	**Age**	**Estimated BMI**	**LCL**	**UCL**	**Estimated BMI**	**LCL**	**UCL**
Low/medium	20 kg/m^2	3	16.3	16.1	16.5	16.1	15.9	16.3
		5	15.7	15.6	15.8	15.7	15.6	15.8
		7	16.3	16.2	16.5	16.2	16.1	16.4
		9	17.6	17.5	17.8	17.5	17.3	17.6
		11	19.0	18.9	19.2	18.9	18.8	19.1
		13	20.1	19.9	20.4	20.3	20.1	20.6
		15	20.4	19.9	20.8	21.3	20.8	21.7
	25 kg/m^2	3	16.2	16.0	16.3	16.0	15.8	16.2
		5	16.0	15.9	16.1	15.9	15.8	16.0
		7	16.8	16.6	16.9	16.7	16.6	16.8
		9	18.1	17.9	18.2	17.9	17.8	18.0
		11	19.5	19.3	19.7	19.4	19.2	19.5
		13	20.6	20.4	20.8	20.8	20.6	21.0
		15	20.9	20.6	21.3	21.8	21.5	22.2
High	20 kg/m^2	3	16.1	16.0	16.2	15.9	15.8	16.0
		5	15.5	15.4	15.5	15.4	15.3	15.5
		7	15.8	15.7	15.9	15.8	15.7	15.9
		9	16.7	16.6	16.8	16.7	16.6	16.9
		11	18.0	17.8	18.1	18.1	17.9	18.2
		13	19.1	19.0	19.3	19.5	19.4	19.7
		15	19.9	19.6	20.2	20.9	20.6	21.2
	25 kg/m^2	3	16.1	16.0	16.2	15.9	15.8	16.1
		5	15.8	15.7	15.9	15.8	15.7	15.8
		7	16.3	16.2	16.3	16.3	16.2	16.4
		9	17.3	17.2	17.4	17.3	17.2	17.4
		11	18.5	18.3	18.6	18.6	18.4	18.7
		13	19.5	19.4	19.7	19.9	19.8	20.1
		15	20.1	19.9	20.4	21.2	20.9	21.4

Figure and Table: Marginal mean BMI values comparing children with maternal BMI of 20 kg/m^2 vs 25 kg/m^2 stratified by sex and ISCED level.

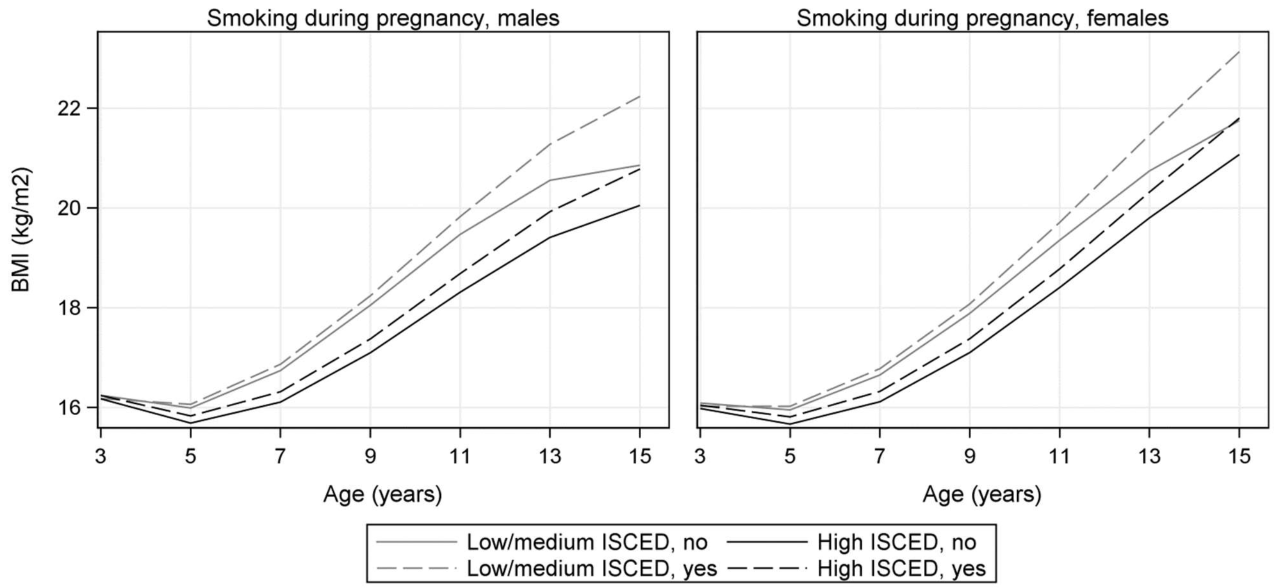

			Males			Females		
**ISCED**	**Smoking during pregnancy**	**Age**	**Estimated BMI**	**LCL**	**UCL**	**Estimated BMI**	**LCL**	**UCL**
Low/medium	No	3	16.2	16.1	16.4	16.1	15.9	16.3
		5	16.0	15.9	16.1	16.0	15.8	16.1
		7	16.7	16.6	16.9	16.7	16.5	16.8
		9	18.1	17.9	18.2	17.9	17.8	18.0
		11	19.5	19.3	19.6	19.4	19.2	19.5
		13	20.6	20.3	20.8	20.8	20.5	21.0
		15	20.9	20.5	21.2	21.8	21.4	22.1
	Yes	3	16.2	15.8	16.5	16.0	15.7	16.4
		5	16.1	15.8	16.3	16.0	15.8	16.2
		7	16.9	16.6	17.1	16.8	16.6	17.0
		9	18.2	18.0	18.5	18.1	17.8	18.3
		11	19.8	19.5	20.2	19.7	19.4	20.1
		13	21.3	20.8	21.7	21.5	21.0	21.9
		15	22.3	21.5	23.0	23.2	22.4	23.9
High	No	3	16.2	16.1	16.3	16.0	15.9	16.1
		5	15.7	15.6	15.8	15.7	15.6	15.7
		7	16.1	16.0	16.2	16.1	16.0	16.2
		9	17.1	17.0	17.2	17.1	17.0	17.2
		11	18.3	18.2	18.4	18.4	18.3	18.5
		13	19.4	19.3	19.6	19.8	19.6	20.0
		15	20.1	19.8	20.3	21.1	20.8	21.3
	Yes	3	16.3	15.8	16.7	16.1	15.6	16.5
		5	15.8	15.6	16.1	15.8	15.6	16.0
		7	16.3	16.1	16.6	16.3	16.1	16.6
		9	17.4	17.1	17.7	17.4	17.1	17.7
		11	18.7	18.3	19.1	18.8	18.4	19.2
		13	19.9	19.4	20.5	20.3	19.8	20.9
		15	20.8	19.9	21.7	21.8	20.9	22.7

Figure and Table: Marginal mean BMI values comparing children with mothers smoking vs not smoking during pregnancy stratified by sex and ISCED level.

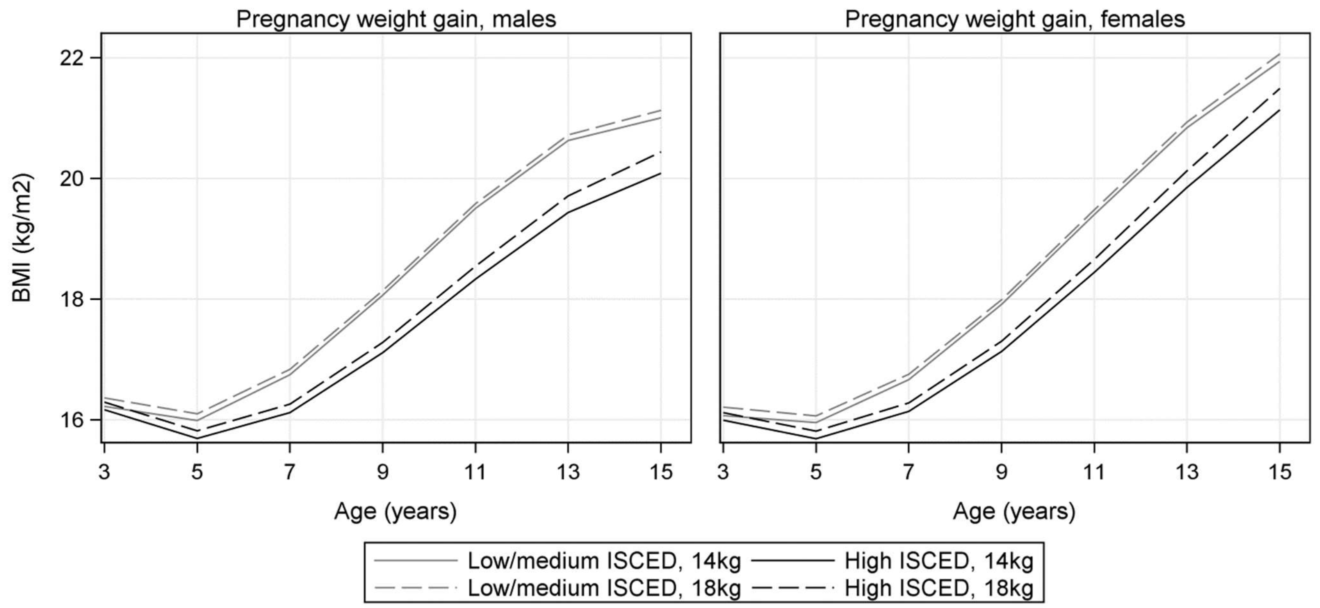

			Males			Females		
**ISCED**	**Pregnancy weight gain**	**Age**	**Estimated BMI**	**LCL**	**UCL**	**Estimated BMI**	**LCL**	**UCL**
Low/medium	14 kg	3	16.2	16.1	16.4	16.1	15.9	16.2
		5	16.0	15.9	16.1	16.0	15.8	16.1
		7	16.7	16.6	16.9	16.7	16.6	16.8
		9	18.1	17.9	18.2	17.9	17.8	18.0
		11	19.5	19.3	19.7	19.4	19.2	19.6
		13	20.6	20.4	20.8	20.8	20.6	21.1
		15	21.0	20.7	21.4	22.0	21.6	22.3
	18 kg	3	16.4	16.2	16.5	16.2	16.0	16.4
		5	16.1	16.0	16.2	16.1	15.9	16.2
		7	16.8	16.7	16.9	16.7	16.6	16.9
		9	18.1	18.0	18.3	18.0	17.8	18.1
		11	19.6	19.4	19.8	19.5	19.3	19.7
		13	20.7	20.5	21.0	20.9	20.7	21.2
		15	21.1	20.7	21.5	22.1	21.7	22.5
High	14 kg	3	16.2	16.0	16.3	16.0	15.9	16.1
		5	15.7	15.6	15.8	15.7	15.6	15.8
		7	16.1	16.0	16.2	16.1	16.1	16.2
		9	17.1	17.0	17.2	17.1	17.0	17.2
		11	18.3	18.2	18.5	18.4	18.3	18.6
		13	19.4	19.3	19.6	19.9	19.7	20.0
		15	20.1	19.8	20.4	21.1	20.9	21.4
	18 kg	3	16.3	16.2	16.4	16.1	16.0	16.2
		5	15.8	15.7	15.9	15.8	15.7	15.9
		7	16.3	16.2	16.3	16.3	16.2	16.4
		9	17.3	17.2	17.4	17.3	17.2	17.4
		11	18.5	18.4	18.7	18.7	18.5	18.8
		13	19.7	19.5	19.9	20.1	19.9	20.3
		15	20.4	20.1	20.8	21.5	21.2	21.8

Figure and Table: Marginal mean BMI values comparing children with maternal weight gain during pregnancy of 14 kg vs 18 kg stratified by sex and ISCED level.

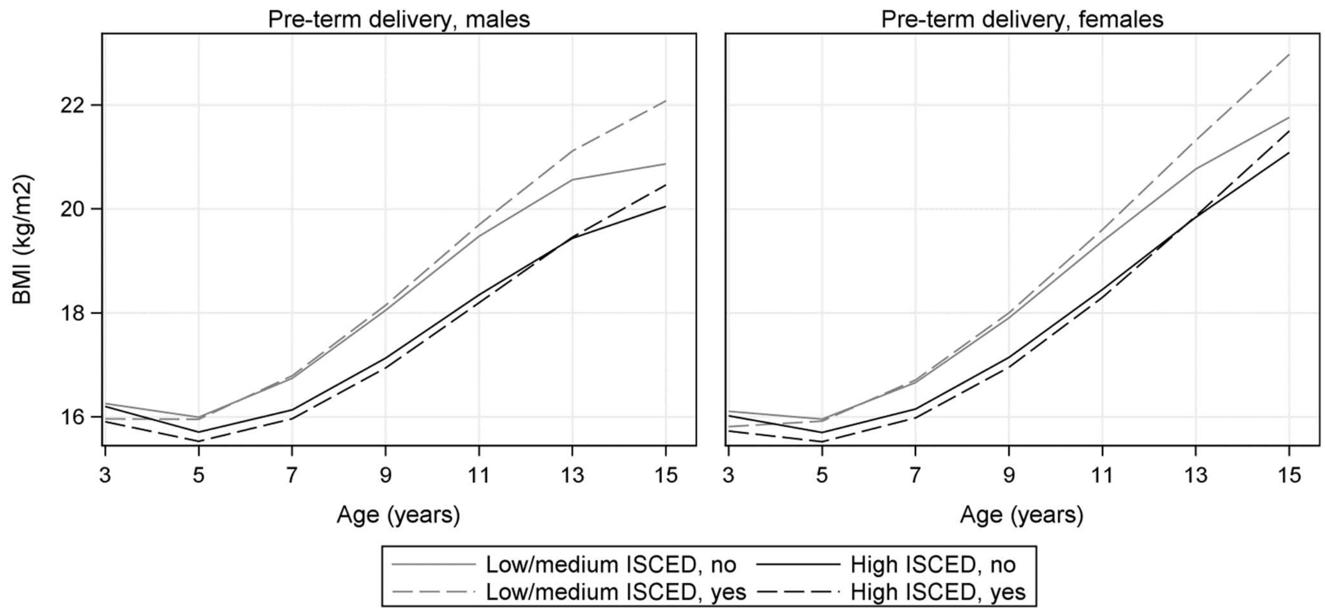

			Males			Females		
**ISCED**	**Preterm delivery**	**Age**	**Estimated BMI**	**LCL**	**UCL**	**Estimated BMI**	**LCL**	**UCL**
Low/medium	No	3	16.3	16.1	16.4	16.1	15.9	16.3
		5	16.0	15.9	16.1	16.0	15.9	16.1
		7	16.7	16.6	16.9	16.7	16.6	16.8
		9	18.1	17.9	18.2	17.9	17.8	18.0
		11	19.5	19.3	19.7	19.4	19.2	19.6
		13	20.6	20.3	20.8	20.8	20.6	21.0
		15	20.9	20.5	21.2	21.8	21.4	22.1
	Yes	3	15.9	15.6	16.3	15.8	15.4	16.2
		5	15.9	15.7	16.2	15.9	15.7	16.1
		7	16.8	16.5	17.0	16.7	16.5	16.9
		9	18.1	17.9	18.4	18.0	17.7	18.3
		11	19.7	19.3	20.0	19.6	19.2	20.0
		13	21.1	20.7	21.6	21.3	20.9	21.8
		15	22.1	21.3	22.8	23.0	22.3	23.7
High	No	3	16.2	16.1	16.3	16.0	15.9	16.1
		5	15.7	15.6	15.8	15.7	15.6	15.8
		7	16.1	16.1	16.2	16.2	16.1	16.2
		9	17.1	17.0	17.2	17.1	17.0	17.2
		11	18.4	18.2	18.5	18.5	18.3	18.6
		13	19.4	19.3	19.6	19.8	19.7	20.0
		15	20.1	19.8	20.3	21.1	20.8	21.4
	Yes	3	15.9	15.7	16.2	15.7	15.5	16.0
		5	15.5	15.4	15.7	15.5	15.4	15.7
		7	16.0	15.8	16.1	16.0	15.8	16.2
		9	16.9	16.7	17.2	16.9	16.7	17.2
		11	18.2	17.9	18.5	18.3	18.0	18.6
		13	19.4	19.1	19.8	19.8	19.5	20.2
		15	20.5	19.8	21.1	21.5	20.9	22.1

Figure and Table: Marginal mean BMI values comparing children with vs without preterm delivery stratified by sex and ISCED level.

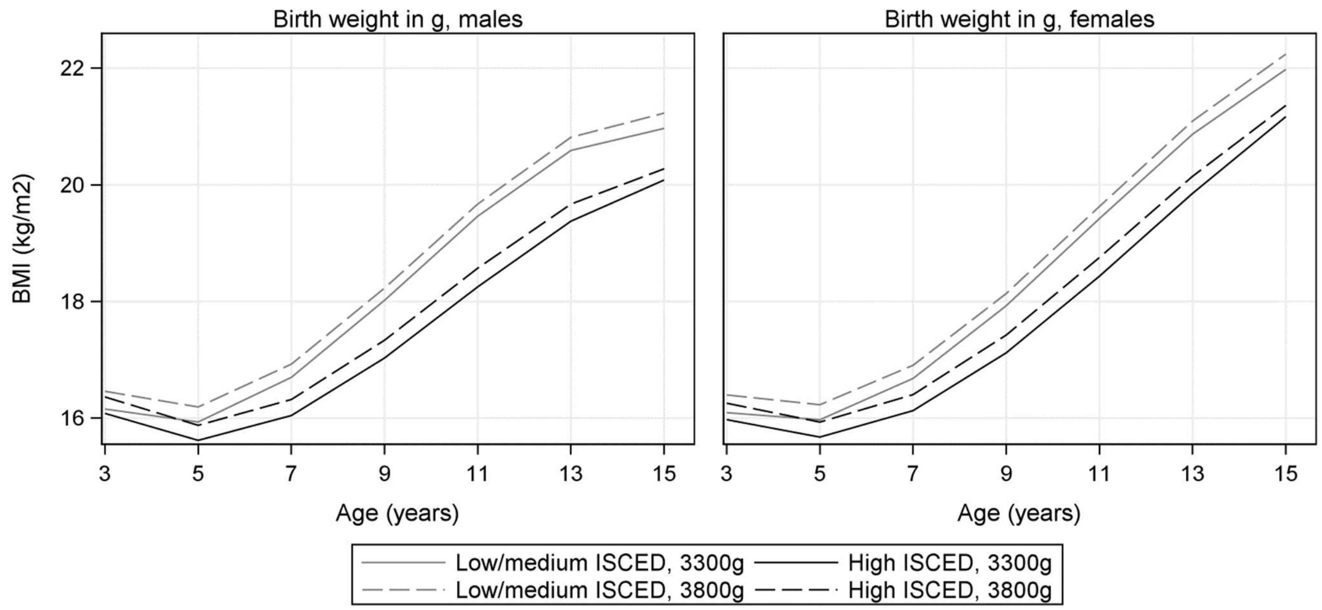

			Males			Females		
ISCED	Birth weight	Age	Estimated BMI	LCL	UCL	Estimated BMI	LCL	UCL
Low/medium	3300 g	3	16.2	16.0	16.3	16.1	15.9	16.3
		5	15.9	15.8	16.0	16.0	15.9	16.1
		7	16.7	16.6	16.8	16.7	16.6	16.8
		9	18.0	17.9	18.1	17.9	17.8	18.1
		11	19.5	19.3	19.6	19.4	19.3	19.6
		13	20.6	20.4	20.8	20.9	20.7	21.1
		15	21.0	20.6	21.3	22.0	21.6	22.3
	3800 g	3	16.5	16.3	16.6	16.4	16.2	16.6
		5	16.2	16.1	16.3	16.2	16.1	16.4
		7	16.9	16.8	17.0	16.9	16.8	17.0
		9	18.2	18.1	18.4	18.1	18.0	18.3
		11	19.7	19.5	19.9	19.6	19.4	19.8
		13	20.8	20.6	21.1	21.1	20.9	21.4
		15	21.2	20.9	21.6	22.3	21.8	22.7
High	3300 g	3	16.1	16.0	16.2	16.0	15.9	16.1
		5	15.6	15.5	15.7	15.7	15.6	15.7
		7	16.0	16.0	16.1	16.1	16.0	16.2
		9	17.0	16.9	17.1	17.1	17.0	17.2
		11	18.3	18.1	18.4	18.4	18.3	18.6
		13	19.4	19.2	19.5	19.9	19.7	20.0
		15	20.1	19.8	20.4	21.2	20.9	21.4
	3800 g	3	16.4	16.2	16.5	16.3	16.1	16.4
		5	15.9	15.8	16.0	15.9	15.8	16.0
		7	16.3	16.2	16.4	16.4	16.3	16.5
		9	17.3	17.2	17.4	17.4	17.3	17.5
		11	18.6	18.4	18.7	18.8	18.6	18.9
		13	19.7	19.5	19.9	20.1	20.0	20.3
		15	20.3	20.0	20.6	21.4	21.0	21.7

Figure and Table: Marginal mean BMI values comparing children with birth weight 3300 g vs 3800 g stratified by sex and ISCED level.

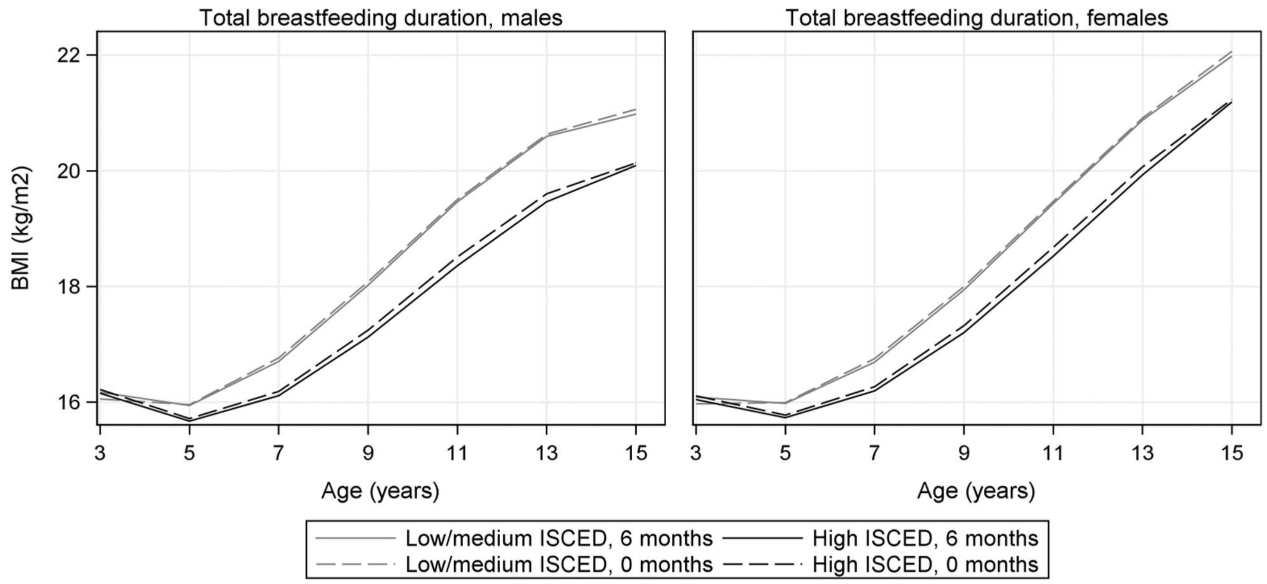

ISCED	Total breastfeeding duration	Age	Males: Estimated BMI	Males: LCL	Males: UCL	Females: Estimated BMI	Females: LCL	Females: UCL
Low/medium	0 months	3	16.1	15.9	16.3	16.0	15.8	16.2
		5	15.9	15.8	16.1	16.0	15.9	16.1
		7	16.8	16.6	16.9	16.7	16.6	16.9
		9	18.1	17.9	18.2	18.0	17.8	18.2
		11	19.5	19.3	19.7	19.5	19.3	19.7
		13	20.6	20.4	20.9	20.9	20.6	21.2
		15	21.0	20.6	21.4	22.0	21.6	22.5
	6 months	3	16.2	16.0	16.3	16.1	15.9	16.3
		5	15.9	15.8	16.0	16.0	15.9	16.1
		7	16.7	16.6	16.8	16.7	16.6	16.8
		9	18.0	17.9	18.2	17.9	17.8	18.1
		11	19.5	19.3	19.6	19.4	19.3	19.6
		13	20.6	20.4	20.8	20.9	20.7	21.1
		15	21.0	20.6	21.3	22.0	21.7	22.3
High	0 months	3	16.2	16.0	16.4	16.1	15.9	16.3
		5	15.7	15.6	15.8	15.8	15.7	15.9
		7	16.2	16.1	16.3	16.3	16.2	16.4
		9	17.2	17.1	17.4	17.3	17.2	17.4
		11	18.5	18.3	18.7	18.7	18.5	18.8
		13	19.6	19.4	19.8	20.1	19.8	20.3
		15	20.1	19.8	20.5	21.2	20.9	21.6
	6 months	3	16.2	16.0	16.3	16.0	15.9	16.2
		5	15.7	15.6	15.7	15.7	15.7	15.8
		7	16.1	16.0	16.2	16.2	16.1	16.3
		9	17.1	17.0	17.2	17.2	17.1	17.3
		11	18.4	18.2	18.5	18.5	18.4	18.7
		13	19.5	19.3	19.6	19.9	19.8	20.1
		15	20.1	19.8	20.4	21.2	20.9	21.5

Figure and Table: Marginal mean BMI values comparing children that where breastfed 0 vs 6 months stratified by sex and ISCED level.

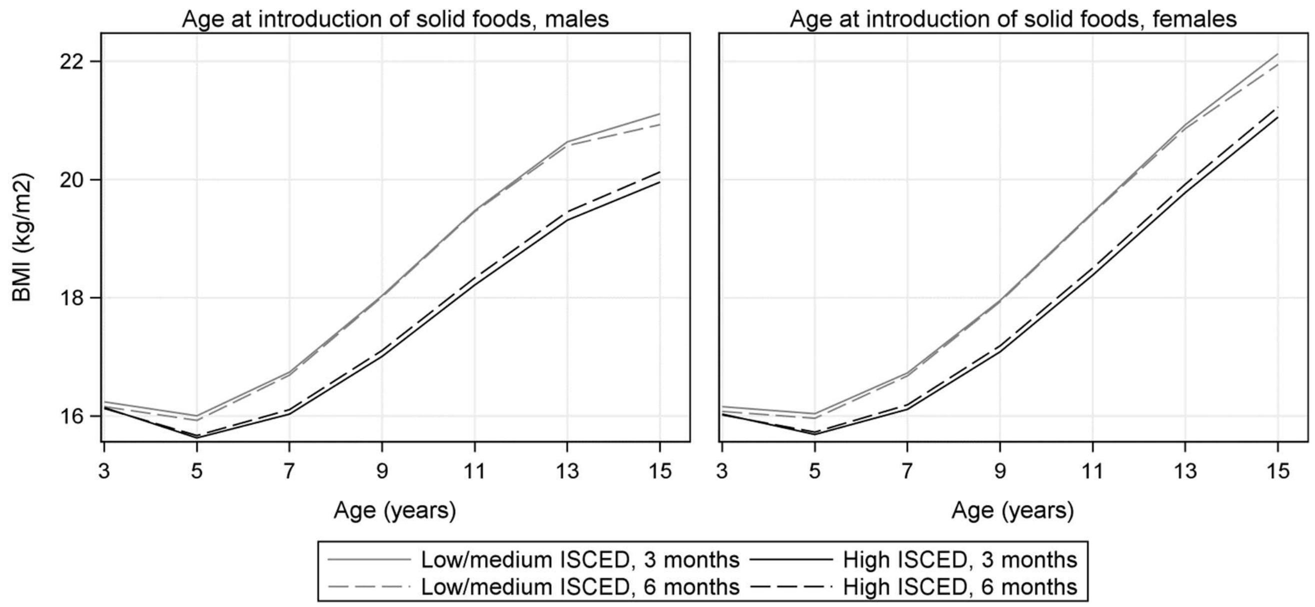

			Males			Females		
**ISCED**	**Age at intro on of solid foods**	**Age**	**Estimated BMI**	**LCL**	**UCL**	**Estimated BMI**	**LCL**	**UCL**
Low/medium	3 months	3	16.2	16.0	16.4	16.1	15.9	16.4
		5	16.0	15.9	16.1	16.0	15.9	16.2
		7	16.7	16.6	16.9	16.7	16.6	16.9
		9	18.0	17.9	18.2	18.0	17.8	18.1
		11	19.5	19.3	19.7	19.4	19.2	19.6
		13	20.6	20.4	20.9	20.9	20.7	21.2
		15	21.1	20.7	21.5	22.1	21.7	22.5
	6 months	3	16.2	16.0	16.3	16.1	15.9	16.3
		5	15.9	15.8	16.0	16.0	15.9	16.1
		7	16.7	16.6	16.8	16.7	16.6	16.8
		9	18.0	17.9	18.1	17.9	17.8	18.1
		11	19.5	19.3	19.6	19.4	19.3	19.6
		13	20.6	20.4	20.8	20.9	20.7	21.1
		15	20.9	20.6	21.3	22.0	21.6	22.3
High	3 months	3	16.1	16.0	16.3	16.0	15.9	16.2
		5	15.6	15.5	15.7	15.7	15.6	15.8
		7	16.0	15.9	16.1	16.1	16.0	16.2
		9	17.0	16.9	17.1	17.1	17.0	17.2
		11	18.2	18.1	18.4	18.4	18.2	18.5
		13	19.3	19.1	19.5	19.8	19.6	20.0
		15	19.9	19.6	20.3	21.0	20.7	21.4
	6 months	3	16.1	16.0	16.2	16.0	15.9	16.1
		5	15.7	15.6	15.7	15.7	15.7	15.8
		7	16.1	16.0	16.2	16.2	16.1	16.3
		9	17.1	17.0	17.2	17.2	17.1	17.3
		11	18.3	18.2	18.5	18.5	18.4	18.6
		13	19.5	19.3	19.6	19.9	19.8	20.1
		15	20.1	19.9	20.4	21.2	21.0	21.5

Figure and Table: Marginal mean BMI values comparing children with age at introduction of solid foods of 3 vs 6 months stratified by sex and ISCED level.

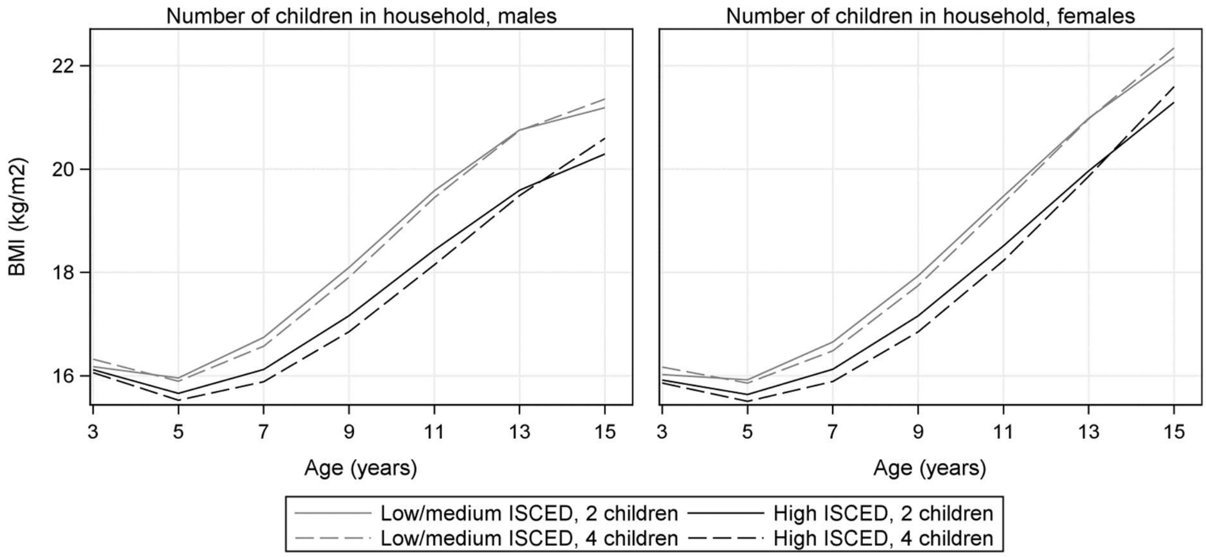

			Males			Females		
**ISCED**	**Number of children**	**Age**	**Estimated BMI**	**LCL**	**UCL**	**Estimated BMI**	**LCL**	**UCL**
Low/medium	2 children	3	16.2	16.0	16.3	16.0	15.9	16.2
		5	16.0	15.9	16.1	15.9	15.8	16.0
		7	16.7	16.6	16.9	16.7	16.5	16.8
		9	18.1	18.0	18.2	17.9	17.8	18.1
		11	19.6	19.4	19.8	19.5	19.3	19.6
		13	20.8	20.5	21.0	21.0	20.8	21.2
		15	21.2	20.8	21.5	22.2	21.8	22.5
	4 children	3	16.3	16.0	16.7	16.2	15.8	16.5
		5	15.9	15.7	16.1	15.9	15.7	16.0
		7	16.6	16.4	16.7	16.5	16.3	16.6
		9	17.9	17.7	18.1	17.7	17.6	17.9
		11	19.4	19.2	19.7	19.3	19.1	19.6
		13	20.7	20.4	21.1	21.0	20.6	21.3
		15	21.3	20.7	22.0	22.3	21.6	23.0
High	2 children	3	16.1	16.0	16.2	15.9	15.8	16.0
		5	15.7	15.6	15.7	15.6	15.6	15.7
		7	16.1	16.0	16.2	16.1	16.0	16.2
		9	17.2	17.1	17.3	17.2	17.1	17.3
		11	18.4	18.3	18.6	18.5	18.4	18.6
		13	19.6	19.4	19.8	20.0	19.8	20.1
		15	20.3	20.0	20.6	21.3	21.0	21.6
	4 children	3	16.1	15.8	16.3	15.9	15.6	16.1
		5	15.5	15.4	15.7	15.5	15.4	15.6
		7	15.9	15.8	16.0	15.9	15.8	16.0
		9	16.8	16.7	17.0	16.8	16.7	17.0
		11	18.1	18.0	18.3	18.2	18.0	18.4
		13	19.5	19.2	19.7	19.9	19.6	20.1
		15	20.6	20.1	21.1	21.6	21.1	22.1

Figure and Table: Marginal mean BMI values comparing children with 2 vs 4 children in household stratified by sex and ISCED level.

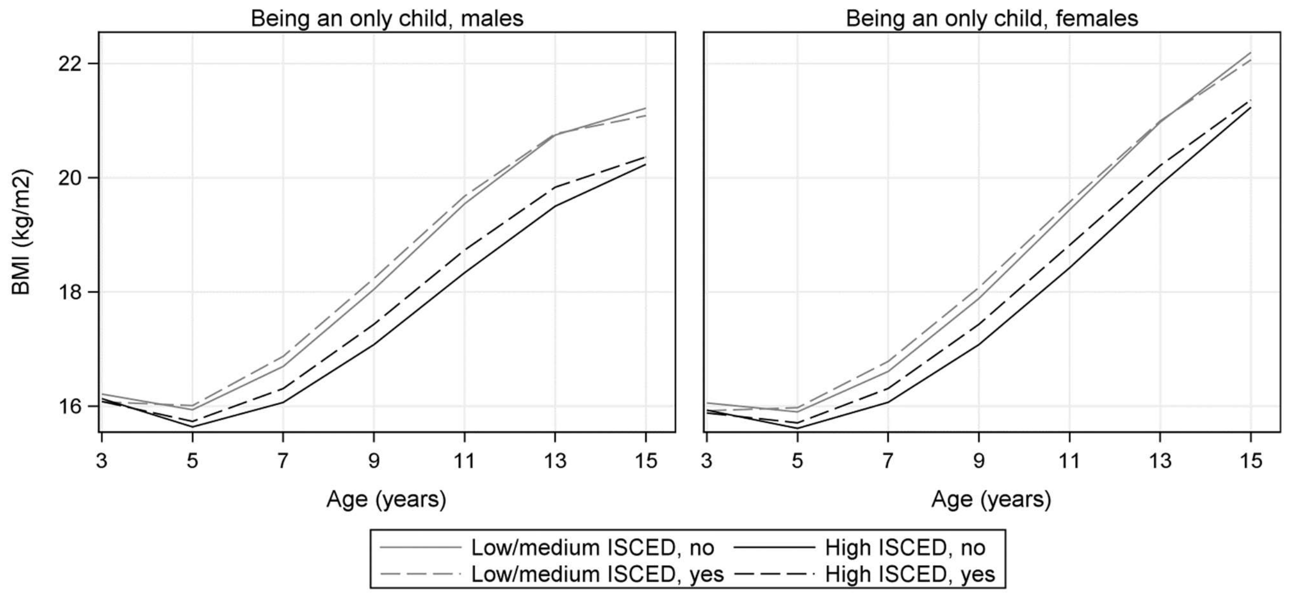

			Males			Females		
ISCED	Being an only child	Age	Estimated BMI	LCL	UCL	Estimated BMI	LCL	UCL
Low/medium	No	3	16.2	16.0	16.4	16.1	15.9	16.2
		5	15.9	15.8	16.0	15.9	15.8	16.0
		7	16.7	16.6	16.8	16.6	16.5	16.7
		9	18.0	17.9	18.2	17.9	17.7	18.0
		11	19.5	19.4	19.7	19.4	19.3	19.6
		13	20.7	20.5	21.0	21.0	20.7	21.2
		15	21.2	20.8	21.6	22.2	21.8	22.6
	Yes	3	16.1	15.9	16.3	15.9	15.7	16.2
		5	16.0	15.9	16.1	16.0	15.8	16.1
		7	16.9	16.7	17.0	16.8	16.6	16.9
		9	18.2	18.1	18.4	18.1	17.9	18.3
		11	19.7	19.5	19.9	19.6	19.4	19.8
		13	20.8	20.5	21.0	21.0	20.7	21.3
		15	21.1	20.6	21.6	22.1	21.6	22.5
High	No	3	16.1	16.0	16.3	15.9	15.8	16.1
		5	15.6	15.6	15.7	15.6	15.5	15.7
		7	16.1	16.0	16.1	16.1	16.0	16.1
		9	17.1	17.0	17.2	17.1	17.0	17.2
		11	18.3	18.2	18.5	18.4	18.3	18.6
		13	19.5	19.3	19.7	19.9	19.7	20.0
		15	20.2	19.9	20.5	21.2	20.9	21.5
	Yes	3	16.1	15.9	16.3	15.9	15.7	16.1
		5	15.7	15.6	15.9	15.7	15.6	15.8
		7	16.3	16.2	16.4	16.3	16.2	16.4
		9	17.4	17.3	17.6	17.4	17.3	17.6
		11	18.7	18.6	18.9	18.8	18.6	19.0
		13	19.8	19.6	20.1	20.2	20.0	20.4
		15	20.4	20.0	20.7	21.4	21.0	21.7

Figure and Table: Marginal mean BMI values comparing children being an only child vs no only child stratified by sex and ISCED level.

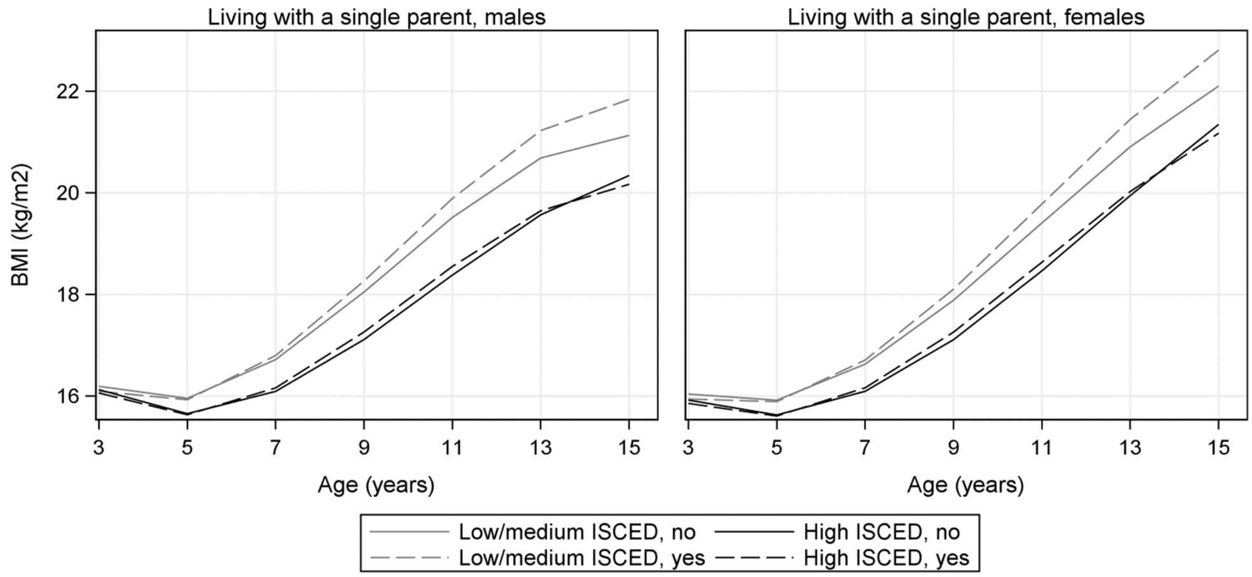

ISCED	Single parent	Age	Males Estimated BMI	Males LCL	Males UCL	Females Estimated BMI	Females LCL	Females UCL
Low/medium	No	3	16.2	16.0	16.4	16.0	15.9	16.2
		5	16.0	15.9	16.1	15.9	15.8	16.0
		7	16.7	16.6	16.8	16.6	16.5	16.7
		9	18.0	17.9	18.2	17.9	17.8	18.0
		11	19.5	19.3	19.7	19.4	19.2	19.6
		13	20.7	20.5	20.9	20.9	20.7	21.1
		15	21.1	20.7	21.5	22.1	21.7	22.4
	Yes	3	16.1	15.8	16.4	15.9	15.7	16.2
		5	15.9	15.8	16.1	15.9	15.8	16.0
		7	16.8	16.7	16.9	16.7	16.6	16.8
		9	18.3	18.1	18.4	18.1	17.9	18.3
		11	19.9	19.7	20.1	19.8	19.6	20.0
		13	21.3	20.9	21.6	21.5	21.2	21.8
		15	21.9	21.3	22.6	22.9	22.3	23.5
High	No	3	16.1	16.0	16.2	15.9	15.8	16.0
		5	15.7	15.6	15.7	15.6	15.6	15.7
		7	16.1	16.0	16.2	16.1	16.0	16.2
		9	17.1	17.0	17.2	17.1	17.0	17.2
		11	18.4	18.3	18.5	18.5	18.3	18.6
		13	19.6	19.4	19.7	19.9	19.8	20.1
		15	20.3	20.1	20.6	21.3	21.1	21.6
	Yes	3	16.1	15.8	16.3	15.8	15.5	16.2
		5	15.6	15.5	15.8	15.6	15.5	15.8
		7	16.2	16.0	16.3	16.2	16.0	16.3
		9	17.3	17.1	17.4	17.3	17.1	17.4
		11	18.5	18.3	18.7	18.6	18.4	18.8
		13	19.6	19.4	19.9	20.0	19.7	20.3
		15	20.1	19.6	20.6	21.1	20.6	21.7

Figure and Table: Marginal mean BMI values comparing children living/not living in a single parent household stratified by sex and ISCED level.

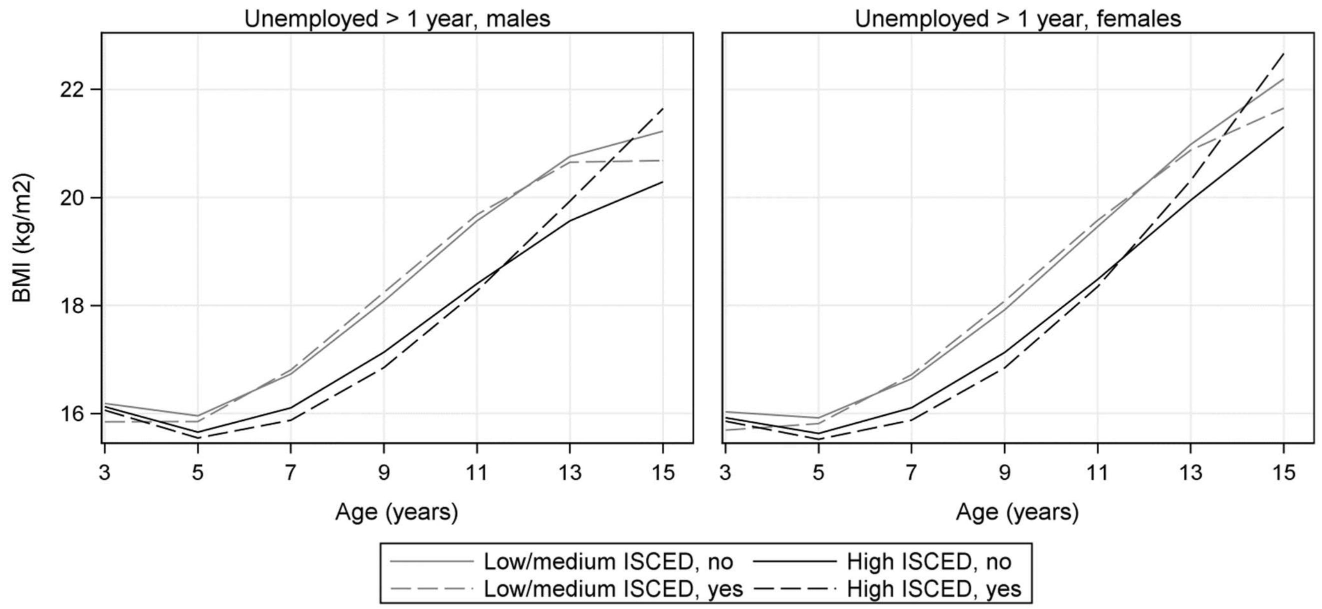

			Males			Females		
ISCED	Unemployment > 1 year	Age	Estimated BMI	LCL	UCL	Estimated BMI	LCL	UCL
Low/medium	No	3	16.2	16.0	16.3	16.0	15.9	16.2
		5	16.0	15.9	16.1	15.9	15.8	16.0
		7	16.7	16.6	16.8	16.6	16.5	16.8
		9	18.1	17.9	18.2	17.9	17.8	18.1
		11	19.6	19.4	19.7	19.5	19.3	19.6
		13	20.8	20.5	21.0	21.0	20.8	21.2
		15	21.2	20.9	21.6	22.2	21.9	22.5
	Yes	3	15.8	15.2	16.5	15.7	15.1	16.3
		5	15.9	15.6	16.1	15.8	15.6	16.1
		7	16.8	16.6	17.0	16.7	16.5	16.9
		9	18.2	18.0	18.5	18.1	17.8	18.3
		11	19.7	19.3	20.0	19.6	19.2	19.9
		13	20.6	20.1	21.2	20.8	20.3	21.4
		15	20.7	19.2	22.1	21.6	20.2	23.1
High	No	3	16.1	16.0	16.2	15.9	15.8	16.0
		5	15.7	15.6	15.7	15.6	15.6	15.7
		7	16.1	16.0	16.2	16.1	16.0	16.2
		9	17.1	17.0	17.2	17.1	17.0	17.2
		11	18.4	18.3	18.5	18.5	18.4	18.6
		13	19.6	19.4	19.7	19.9	19.8	20.1
		15	20.3	20.0	20.6	21.3	21.0	21.6
	Yes	3	16.1	15.2	16.9	15.9	15.0	16.7
		5	15.5	15.2	15.9	15.5	15.2	15.9
		7	15.9	15.6	16.2	15.9	15.6	16.2
		9	16.8	16.6	17.1	16.8	16.6	17.1
		11	18.3	17.9	18.7	18.3	18.0	18.7
		13	19.9	19.3	20.6	20.3	19.6	21.0
		15	21.6	19.8	23.5	22.6	20.8	24.5

Figure and Table: Marginal mean BMI values comparing children with vs without parental unemployment stratified by sex and ISCED level.

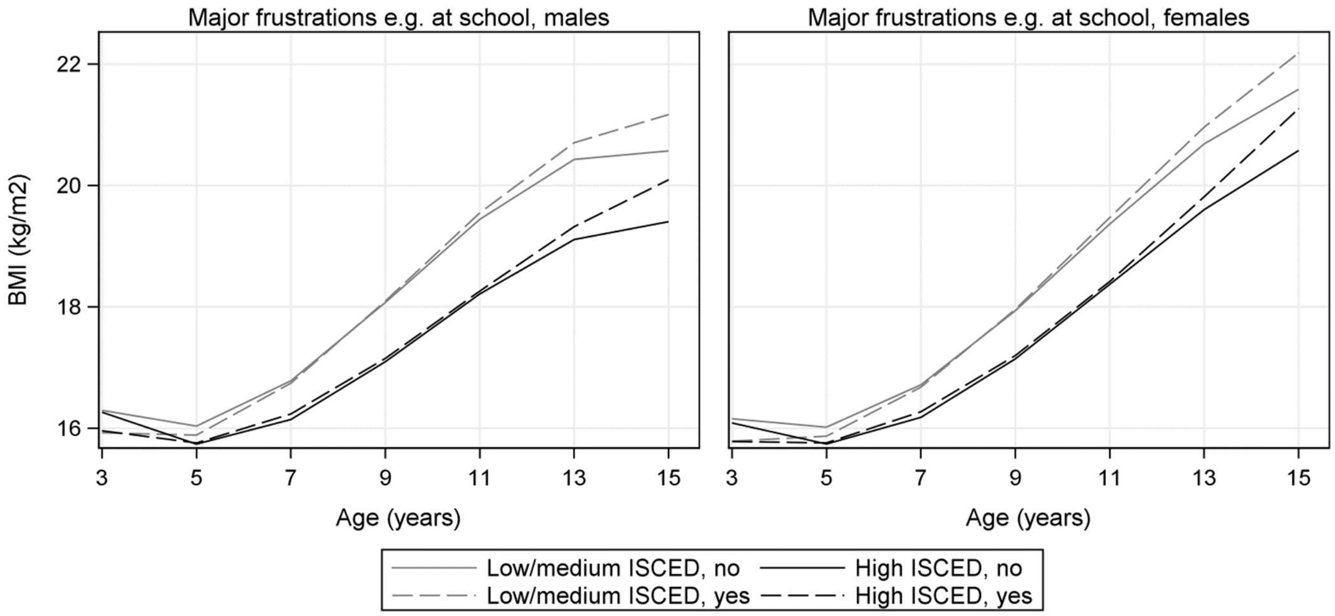

			Males			Females		
**ISCED**	**Major frustrations**	**Age**	**Estimated BMI**	**LCL**	**UCL**	**Estimated BMI**	**LCL**	**UCL**
Low/medium	No	3	16.3	16.1	16.5	16.2	16.0	16.3
		5	16.0	15.9	16.1	16.0	15.9	16.1
		7	16.8	16.7	16.9	16.7	16.6	16.8
		9	18.1	17.9	18.2	17.9	17.8	18.1
		11	19.4	19.3	19.6	19.4	19.2	19.5
		13	20.5	20.2	20.7	20.7	20.5	20.9
		15	20.7	20.2	21.1	21.7	21.3	22.1
	Yes	3	16.1	15.5	16.7	16.0	15.4	16.6
		5	15.8	15.6	16.1	15.8	15.6	16.1
		7	16.7	16.5	16.9	16.6	16.4	16.8
		9	18.1	18.0	18.3	18.0	17.8	18.2
		11	19.7	19.4	19.9	19.6	19.3	19.9
		13	20.7	19.6	21.8	21.0	19.8	22.1
		15	20.7	17.7	23.8	21.8	18.7	24.8
High	No	3	16.3	16.1	16.4	16.1	16.0	16.2
		5	15.7	15.7	15.8	15.7	15.7	15.8
		7	16.1	16.1	16.2	16.2	16.1	16.3
		9	17.1	17.0	17.2	17.1	17.0	17.2
		11	18.2	18.1	18.4	18.4	18.3	18.5
		13	19.1	19.0	19.3	19.6	19.4	19.8
		15	19.5	19.1	19.8	20.6	20.3	21.0
	Yes	3	15.7	15.1	16.2	15.5	15.0	16.0
		5	15.8	15.6	16.0	15.8	15.6	16.0
		7	16.3	16.1	16.4	16.3	16.2	16.4
		9	17.1	16.9	17.2	17.1	17.0	17.3
		11	18.4	18.1	18.6	18.5	18.3	18.8
		13	20.2	19.2	21.2	20.7	19.7	21.7
		15	22.7	19.9	25.5	23.8	21.0	26.6

Figure and Table: Marginal mean BMI values comparing children with/without major frustrations stratified by sex and ISCED level.

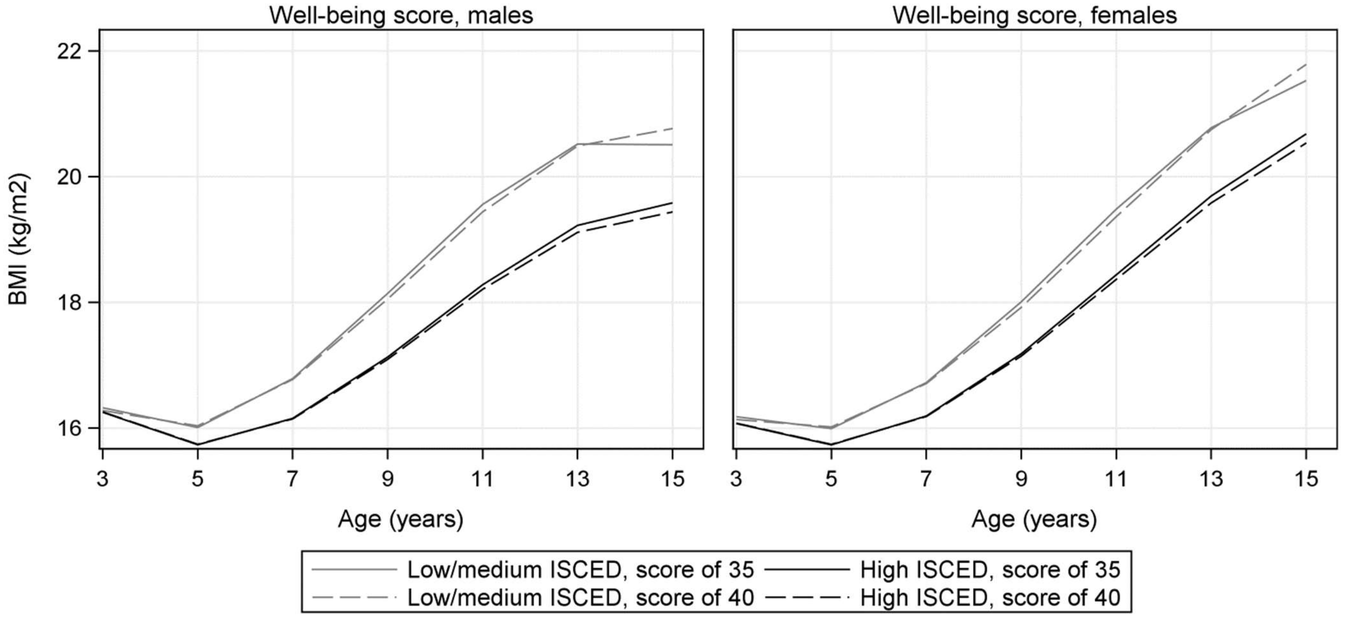

			Males			Females		
**ISCED**	**Wellbeing Score**	**Age**	**Estimated BMI**	**LCL**	**UCL**	**Estimated BMI**	**LCL**	**UCL**
Low/medium	Score of 35	3	16.3	16.1	16.5	16.2	16.0	16.4
		5	16.0	15.9	16.1	16.0	15.9	16.1
		7	16.8	16.7	16.9	16.7	16.6	16.8
		9	18.2	18.0	18.3	18.0	17.9	18.2
		11	19.6	19.4	19.7	19.5	19.3	19.7
		13	20.5	20.2	20.7	20.8	20.5	21.0
		15	20.4	20.0	20.9	21.5	21.0	21.9
	Score of 40	3	16.3	16.1	16.5	16.1	16.0	16.3
		5	16.0	15.9	16.1	16.0	15.9	16.1
		7	16.8	16.7	16.9	16.7	16.6	16.8
		9	18.1	17.9	18.2	17.9	17.8	18.1
		11	19.4	19.3	19.6	19.4	19.2	19.5
		13	20.5	20.2	20.7	20.7	20.5	21.0
		15	20.7	20.3	21.2	21.8	21.3	22.2
High	Score of 35	3	16.3	16.1	16.4	16.1	15.9	16.3
		5	15.7	15.7	15.8	15.7	15.6	15.8
		7	16.2	16.1	16.3	16.2	16.1	16.3
		9	17.1	17.0	17.2	17.2	17.1	17.3
		11	18.3	18.2	18.4	18.4	18.3	18.6
		13	19.2	19.0	19.4	19.7	19.5	19.9
		15	19.6	19.2	20.0	20.7	20.3	21.0
	Score of 40	3	16.3	16.1	16.4	16.1	15.9	16.2
		5	15.7	15.7	15.8	15.7	15.7	15.8
		7	16.1	16.1	16.2	16.2	16.1	16.3
		9	17.1	17.0	17.2	17.1	17.0	17.2
		11	18.2	18.1	18.3	18.4	18.2	18.5
		13	19.1	18.9	19.3	19.6	19.4	19.8
		15	19.5	19.1	19.8	20.5	20.2	20.9

Figure and Table: Marginal mean BMI values comparing children with well-being score of 35 vs 40 stratified by sex and ISCED level.

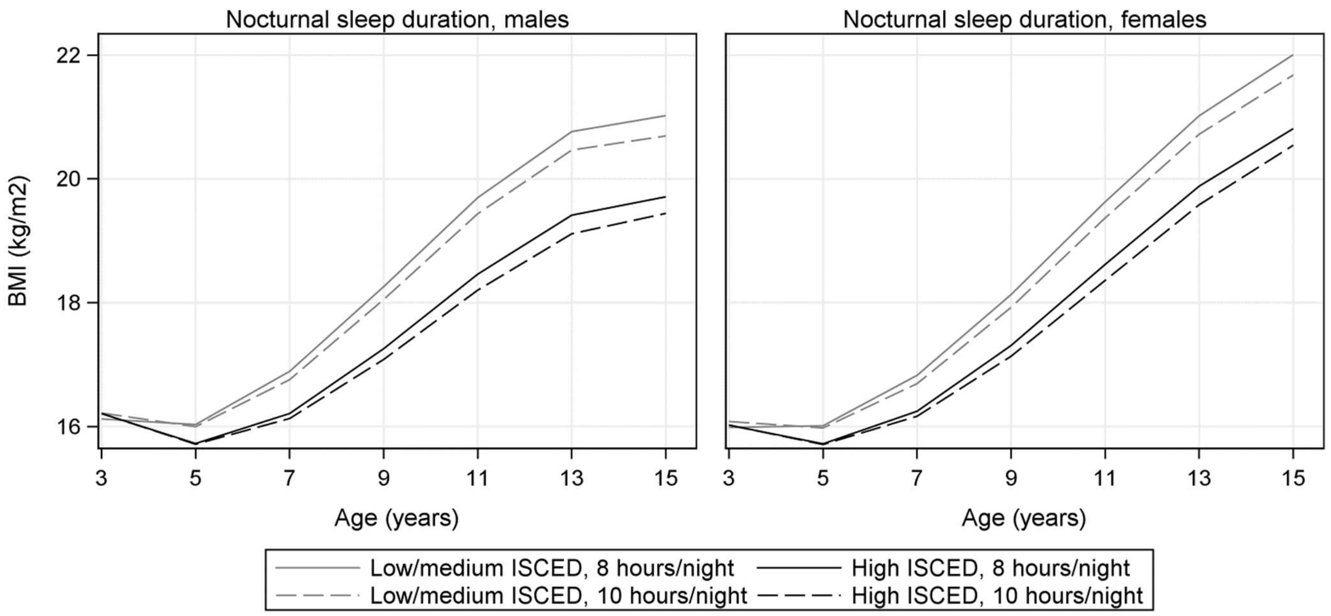

			Males			Females		
ISCED	Nocturnal sleep duration	Age	Estimated BMI	LCL	UCL	Estimated BMI	LCL	UCL
Low/medium	8 hours/night	3	16.1	15.8	16.4	16.0	15.7	16.3
		5	16.0	15.9	16.2	16.0	15.8	16.2
		7	16.9	16.8	17.1	16.8	16.7	17.0
		9	18.3	18.1	18.4	18.1	18.0	18.3
		11	19.7	19.5	19.9	19.6	19.4	19.8
		13	20.8	20.5	21.0	21.0	20.8	21.3
		15	21.0	20.5	21.5	22.0	21.5	22.5
	10 hours/night	3	16.2	16.1	16.4	16.1	15.9	16.3
		5	16.0	15.9	16.1	16.0	15.9	16.1
		7	16.8	16.6	16.9	16.7	16.6	16.8
		9	18.0	17.9	18.2	17.9	17.8	18.1
		11	19.4	19.3	19.6	19.4	19.2	19.5
		13	20.5	20.2	20.7	20.7	20.5	21.0
		15	20.7	20.2	21.2	21.7	21.2	22.2
High	8 hours/night	3	16.2	16.0	16.4	16.0	15.8	16.2
		5	15.7	15.6	15.9	15.7	15.6	15.9
		7	16.2	16.1	16.3	16.2	16.1	16.4
		9	17.2	17.1	17.4	17.3	17.2	17.4
		11	18.4	18.3	18.6	18.6	18.4	18.8
		13	19.4	19.2	19.6	19.9	19.7	20.1
		15	19.8	19.4	20.2	20.9	20.5	21.2
	10 hours/night	3	16.2	16.1	16.3	16.0	15.9	16.1
		5	15.7	15.6	15.8	15.7	15.6	15.8
		7	16.1	16.0	16.2	16.2	16.1	16.2
		9	17.1	17.0	17.2	17.1	17.0	17.2
		11	18.2	18.1	18.3	18.4	18.2	18.5
		13	19.1	18.9	19.3	19.6	19.4	19.8
		15	19.5	19.1	19.9	20.6	20.1	21.0

Figure and Table: Marginal mean BMI values comparing children with 8 vs 10 hours nocturnal sleep duration stratified by sex and ISCED level.

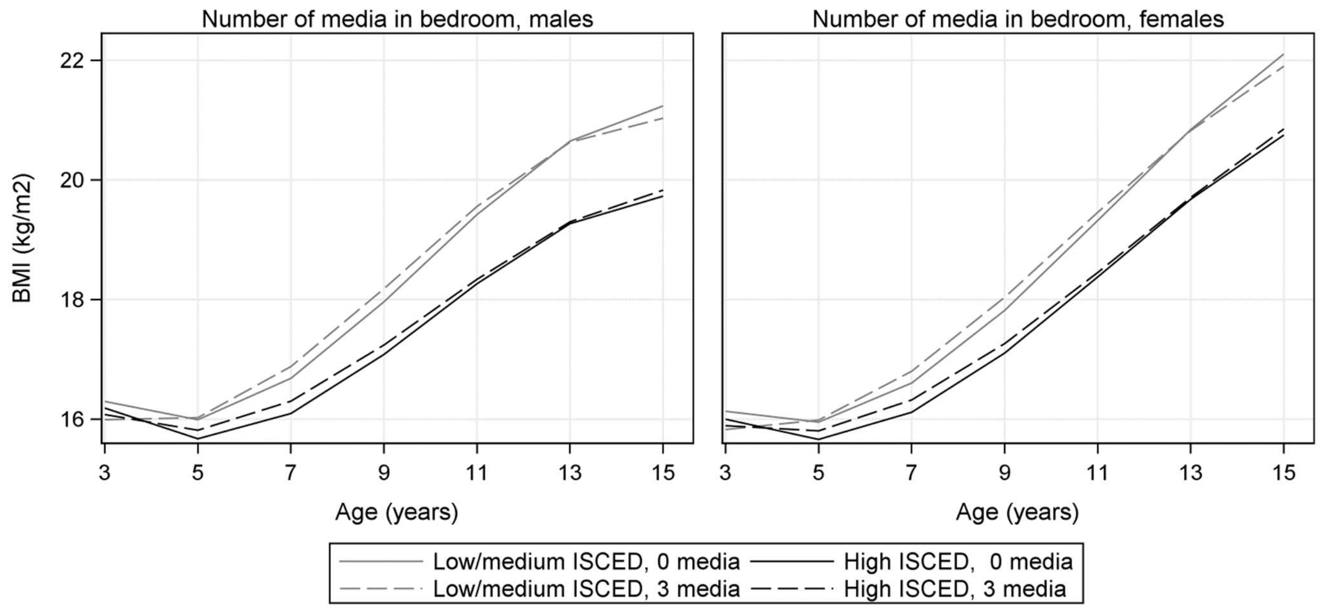

ISCED	Number of media in bedroom	Age	Males: Estimated BMI	Males: LCL	Males: UCL	Females: Estimated BMI	Females: LCL	Females: UCL
Low/medium	0 media	3	16.3	16.1	16.5	16.1	15.9	16.3
		5	16.0	15.9	16.1	15.9	15.8	16.0
		7	16.7	16.6	16.8	16.6	16.5	16.7
		9	18.0	17.8	18.1	17.8	17.7	18.0
		11	19.4	19.2	19.6	19.3	19.1	19.5
		13	20.6	20.3	20.9	20.8	20.5	21.1
		15	21.1	20.4	21.8	22.0	21.4	22.6
	3 media	3	16.0	15.7	16.2	15.8	15.5	16.1
		5	16.0	15.9	16.2	16.0	15.9	16.1
		7	16.9	16.8	17.0	16.8	16.7	16.9
		9	18.2	18.1	18.3	18.0	17.9	18.2
		11	19.6	19.4	19.7	19.5	19.3	19.6
		13	20.6	20.4	20.9	20.8	20.6	21.1
		15	21.0	20.5	21.4	21.9	21.5	22.3
High	0 media	3	16.2	16.1	16.3	16.0	15.9	16.1
		5	15.7	15.6	15.7	15.7	15.6	15.7
		7	16.1	16.0	16.2	16.1	16.0	16.2
		9	17.1	17.0	17.2	17.1	17.0	17.2
		11	18.3	18.1	18.4	18.4	18.2	18.5
		13	19.3	19.0	19.5	19.7	19.5	19.9
		15	19.7	19.3	20.2	20.8	20.3	21.2
	3 media	3	16.1	15.8	16.4	15.9	15.6	16.2
		5	15.8	15.7	15.9	15.8	15.7	15.9
		7	16.3	16.2	16.4	16.3	16.2	16.4
		9	17.2	17.1	17.3	17.3	17.1	17.4
		11	18.3	18.2	18.5	18.5	18.3	18.6
		13	19.3	19.1	19.5	19.7	19.5	19.9
		15	19.8	19.5	20.2	20.9	20.5	21.2

Figure and Table: Marginal mean BMI values comparing children with 0 vs 3 media in bedroom stratified by sex and ISCED level.

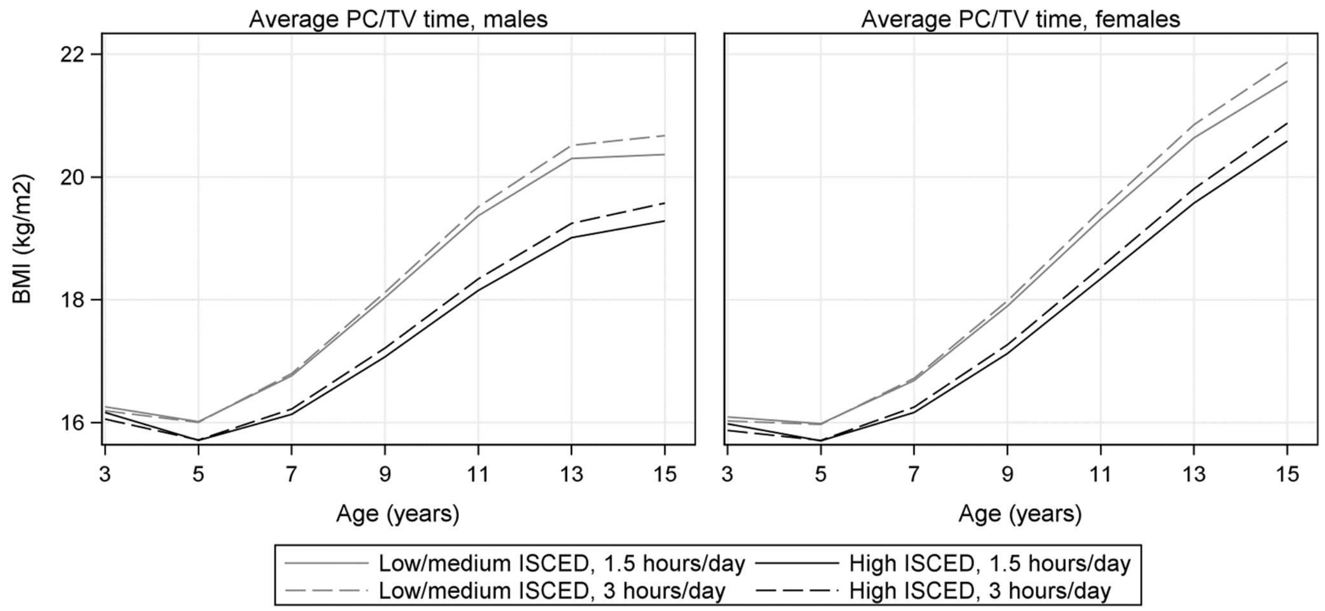

			Males			Females		
**ISCED**	**Average PC/TV time**	**Age**	**Estimated BMI**	**LCL**	**UCL**	**Estimated BMI**	**LCL**	**UCL**
Low/medium	1.5 hours/day	3	16.3	16.1	16.4	16.1	15.9	16.3
		5	16.0	15.9	16.1	16.0	15.9	16.1
		7	16.8	16.7	16.9	16.7	16.6	16.8
		9	18.0	17.9	18.2	17.9	17.8	18.0
		11	19.4	19.2	19.5	19.3	19.1	19.5
		13	20.3	20.0	20.5	20.6	20.4	20.9
		15	20.3	19.8	20.8	21.6	21.1	22.0
	3 hours/day	3	16.2	16.0	16.4	16.0	15.8	16.3
		5	16.0	15.9	16.1	16.0	15.8	16.1
		7	16.8	16.7	16.9	16.7	16.6	16.8
		9	18.1	18.0	18.3	18.0	17.8	18.1
		11	19.5	19.3	19.7	19.5	19.3	19.6
		13	20.5	20.3	20.7	20.9	20.6	21.1
		15	20.7	20.2	21.1	21.9	21.4	22.3
High	1.5 hours/day	3	16.2	16.0	16.3	16.0	15.8	16.1
		5	15.7	15.6	15.8	15.7	15.6	15.8
		7	16.1	16.1	16.2	16.2	16.1	16.2
		9	17.1	17.0	17.2	17.1	17.0	17.2
		11	18.2	18.0	18.3	18.3	18.2	18.5
		13	19.0	18.8	19.2	19.6	19.4	19.8
		15	19.3	18.9	19.7	20.6	20.3	21.0
	3 hours/day	3	16.0	15.8	16.3	15.9	15.6	16.1
		5	15.7	15.6	15.8	15.7	15.6	15.8
		7	16.2	16.1	16.3	16.2	16.2	16.3
		9	17.2	17.1	17.3	17.3	17.2	17.4
		11	18.3	18.2	18.5	18.5	18.4	18.7
		13	19.3	19.1	19.4	19.8	19.6	20.0
		15	19.6	19.2	20.0	20.9	20.5	21.2

Figure and Table: Marginal mean BMI values comparing children with average TV/PC time of 1.5 vs 3 hours/day stratified by sex and ISCED level.

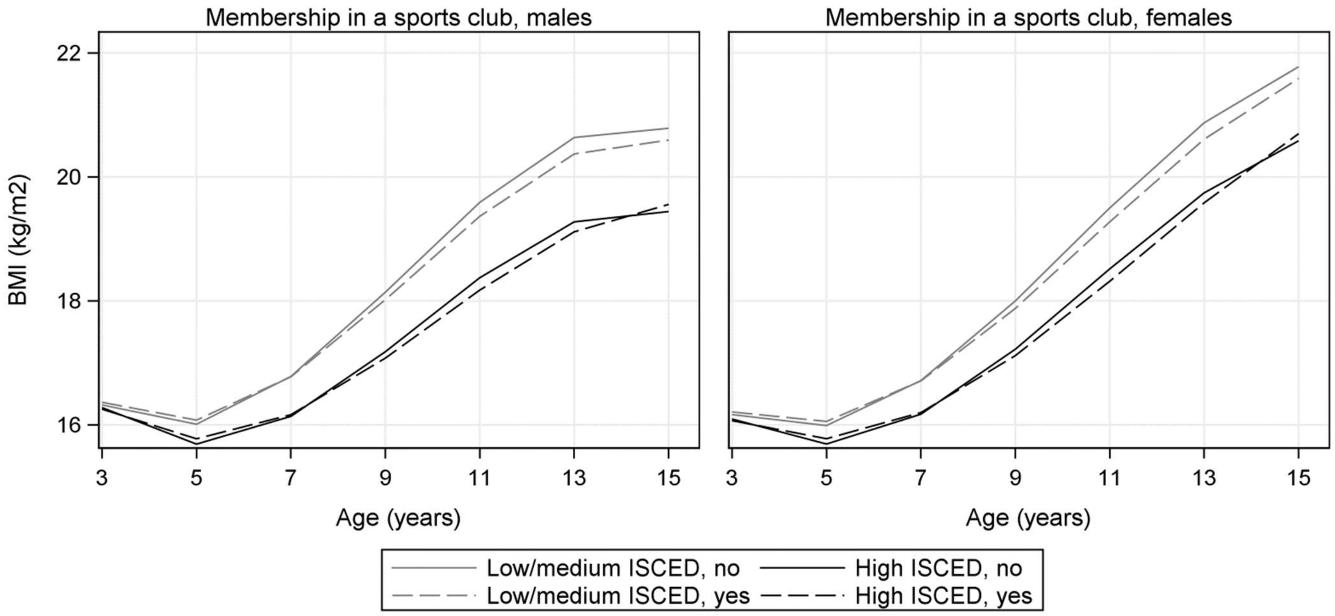

			Males			Females		
**ISCED**	**Membership in sports club**	**Age**	**Estimated BMI**	**LCL**	**UCL**	**Estimated BMI**	**LCL**	**UCL**
Low/medium	No	3	16.4	15.9	16.9	16.3	15.7	16.8
		5	16.1	15.9	16.3	16.1	15.9	16.3
		7	16.8	16.6	16.9	16.7	16.5	16.9
		9	17.9	17.7	18.1	17.8	17.6	17.9
		11	19.1	18.9	19.4	19.0	18.8	19.3
		13	20.1	19.8	20.4	20.3	20.0	20.7
		15	20.4	19.6	21.2	21.4	20.6	22.2
	Yes	3	16.4	16.1	16.7	16.2	15.9	16.5
		5	16.1	16.0	16.2	16.1	15.9	16.2
		7	16.8	16.7	16.9	16.7	16.6	16.8
		9	18.0	17.9	18.2	17.9	17.7	18.0
		11	19.4	19.2	19.5	19.3	19.1	19.4
		13	20.4	20.1	20.6	20.6	20.4	20.9
		15	20.6	20.1	21.1	21.6	21.1	22.1
High	No	3	16.2	15.9	16.6	16.0	15.7	16.4
		5	15.9	15.7	16.0	15.9	15.7	16.0
		7	16.2	16.1	16.3	16.2	16.1	16.3
		9	17.0	16.8	17.1	17.0	16.9	17.2
		11	18.0	17.8	18.2	18.1	17.9	18.3
		13	19.0	18.7	19.2	19.4	19.1	19.7
		15	19.7	19.1	20.2	20.8	20.2	21.4
	Yes	3	16.3	16.1	16.4	16.1	15.9	16.3
		5	15.8	15.7	15.9	15.8	15.7	15.9
		7	16.2	16.1	16.2	16.2	16.1	16.3
		9	17.1	17.0	17.2	17.1	17.0	17.2
		11	18.2	18.0	18.3	18.3	18.2	18.4
		13	19.1	18.9	19.3	19.6	19.4	19.8
		15	19.5	19.2	19.9	20.7	20.3	21.1

Figure and Table: Marginal mean BMI values comparing children with/without membership in sports club stratified by sex and ISCED level.

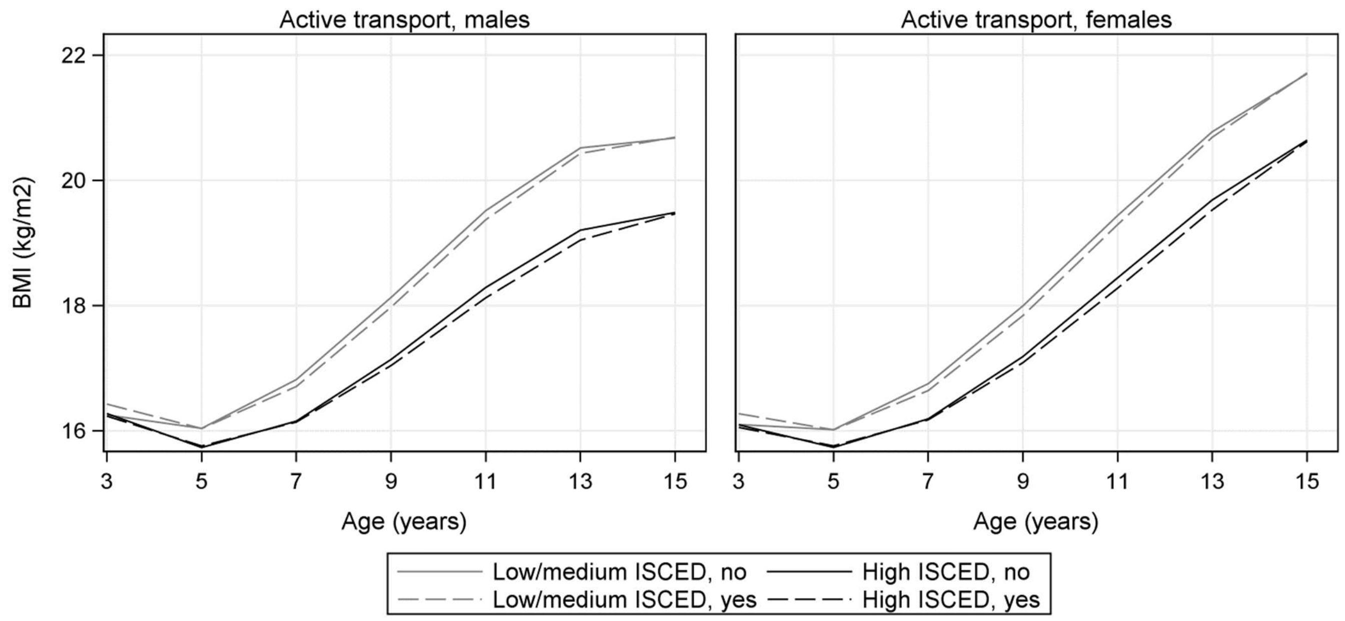

			Males			Females		
**ISCED**	**Active transport**	**Age**	**Estimated BMI**	**LCL**	**UCL**	**Estimated BMI**	**LCL**	**UCL**
Low/medium	No	3	16.3	16.1	16.4	16.1	15.9	16.3
		5	16.0	15.9	16.1	16.0	15.9	16.1
		7	16.8	16.7	16.9	16.8	16.6	16.9
		9	18.1	18.0	18.3	18.0	17.9	18.1
		11	19.5	19.3	19.7	19.4	19.3	19.6
		13	20.5	20.3	20.8	20.8	20.5	21.0
		15	20.6	20.2	21.1	21.7	21.2	22.1
	Yes	3	16.4	16.2	16.6	16.3	16.0	16.5
		5	16.0	15.9	16.2	16.0	15.9	16.1
		7	16.7	16.6	16.8	16.6	16.5	16.8
		9	18.0	17.8	18.1	17.8	17.7	18.0
		11	19.4	19.2	19.5	19.3	19.1	19.5
		13	20.4	20.2	20.7	20.7	20.4	20.9
		15	20.7	20.2	21.2	21.7	21.2	22.3
High	No	3	16.3	16.1	16.4	16.1	16.0	16.2
		5	15.7	15.7	15.8	15.7	15.7	15.8
		7	16.2	16.1	16.2	16.2	16.1	16.3
		9	17.1	17.0	17.2	17.2	17.1	17.3
		11	18.3	18.2	18.4	18.4	18.3	18.6
		13	19.2	19.0	19.4	19.7	19.5	19.9
		15	19.5	19.1	19.9	20.6	20.3	21.0
	Yes	3	16.2	16.1	16.4	16.0	15.9	16.2
		5	15.8	15.7	15.8	15.8	15.7	15.9
		7	16.1	16.0	16.2	16.2	16.1	16.3
		9	17.0	16.9	17.1	17.1	17.0	17.2
		11	18.1	18.0	18.3	18.3	18.1	18.4
		13	19.1	18.8	19.3	19.5	19.3	19.7
		15	19.5	19.1	19.9	20.6	20.2	21.0

Figure and Table: Marginal mean BMI values comparing using/not using an active form of transport stratified by sex and ISCED level.

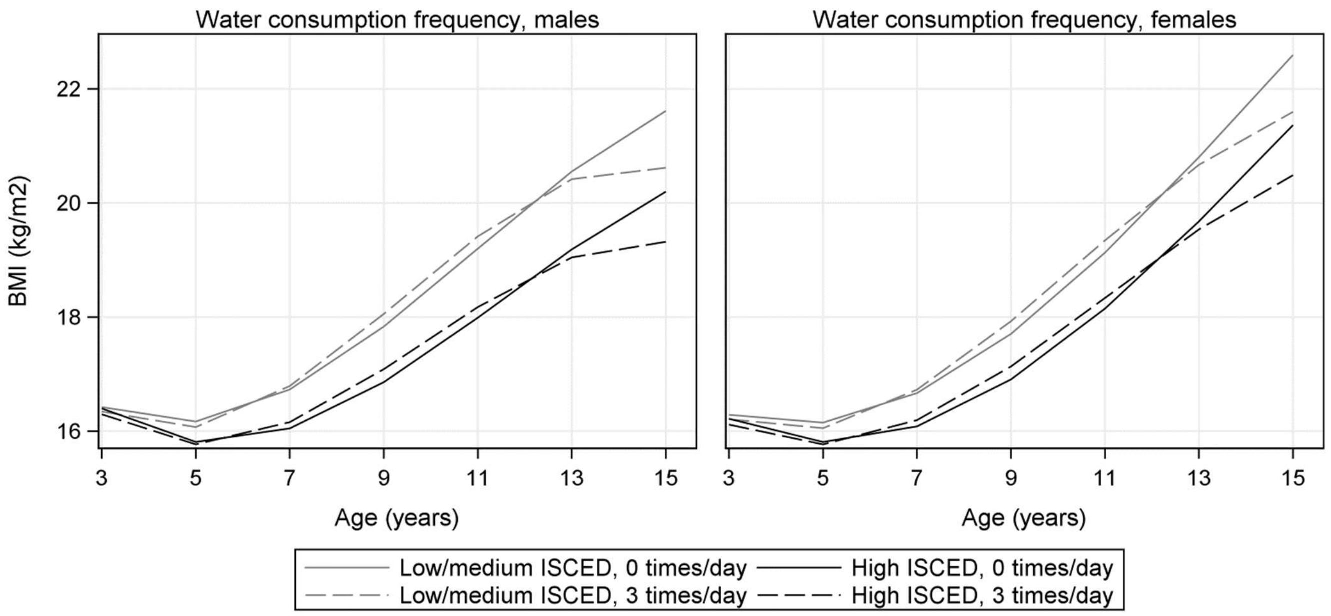

			Males			Females		
ISCED	Water consumption	Age	Estimated BMI	LCL	UCL	Estimated BMI	LCL	UCL
Low/medium	0 times/day	3	16.5	16.2	16.7	16.3	16.0	16.6
		5	16.2	16.0	16.3	16.2	16.0	16.3
		7	16.7	16.6	16.9	16.7	16.5	16.8
		9	17.8	17.7	18.0	17.7	17.5	17.9
		11	19.2	19.0	19.4	19.1	18.9	19.3
		13	20.5	20.3	20.8	20.8	20.5	21.1
		15	21.6	21.0	22.2	22.6	22.0	23.2
	3 times/day	3	16.4	16.2	16.5	16.2	16.0	16.4
		5	16.1	16.0	16.2	16.1	16.0	16.2
		7	16.8	16.7	16.9	16.7	16.6	16.8
		9	18.1	17.9	18.2	17.9	17.8	18.1
		11	19.4	19.2	19.6	19.3	19.2	19.5
		13	20.4	20.2	20.6	20.7	20.4	20.9
		15	20.6	20.1	21.0	21.6	21.1	22.0
High	0 times/day	3	16.4	16.2	16.6	16.2	16.0	16.4
		5	15.8	15.7	15.9	15.8	15.7	15.9
		7	16.0	15.9	16.1	16.1	16.0	16.2
		9	16.9	16.7	17.0	16.9	16.8	17.0
		11	18.0	17.8	18.2	18.1	18.0	18.3
		13	19.2	18.9	19.4	19.7	19.4	19.9
		15	20.2	19.7	20.7	21.4	20.9	21.8
	3 times/day	3	16.3	16.2	16.4	16.1	16.0	16.2
		5	15.8	15.7	15.8	15.8	15.7	15.8
		7	16.2	16.1	16.2	16.2	16.1	16.3
		9	17.1	17.0	17.2	17.1	17.0	17.2
		11	18.2	18.0	18.3	18.3	18.2	18.5
		13	19.1	18.9	19.3	19.5	19.4	19.7
		15	19.3	19.0	19.7	20.5	20.1	20.8

Figure and Table: Marginal mean BMI values comparing children consuming water 0 vs 3 times/day stratified by sex and ISCED level.

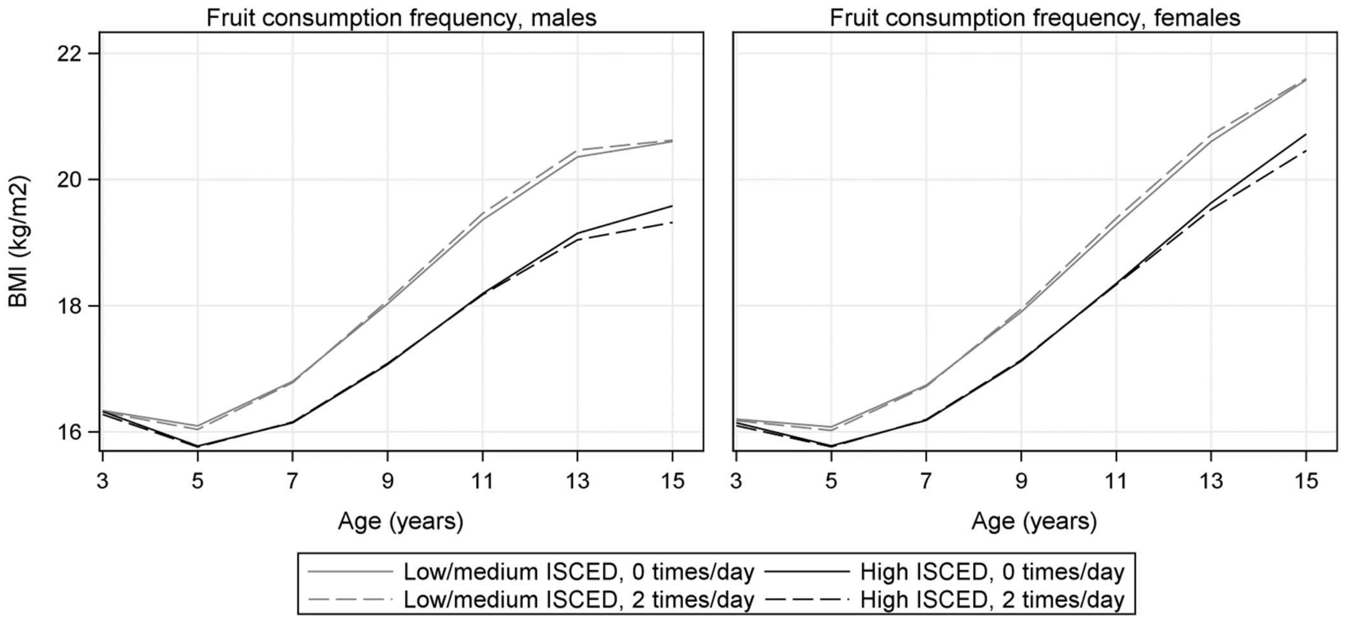

			Males			Females		
ISCED	Fruit consumption	Age	Estimated BMI	LCL	UCL	Estimated BMI	LCL	UCL
Low/medium	0 times/day	3	16.3	16.1	16.6	16.2	16.0	16.4
		5	16.1	16.0	16.2	16.1	16.0	16.2
		7	16.8	16.7	16.9	16.7	16.6	16.9
		9	18.0	17.9	18.2	17.9	17.8	18.0
		11	19.4	19.2	19.5	19.3	19.1	19.5
		13	20.3	20.1	20.6	20.6	20.3	20.8
		15	20.6	20.1	21.1	21.6	21.1	22.1
	2 times/day	3	16.3	16.1	16.5	16.2	16.0	16.4
		5	16.0	15.9	16.1	16.0	15.9	16.1
		7	16.8	16.7	16.9	16.7	16.6	16.8
		9	18.1	17.9	18.2	18.0	17.8	18.1
		11	19.5	19.3	19.6	19.4	19.2	19.6
		13	20.5	20.2	20.7	20.7	20.5	20.9
		15	20.6	20.1	21.0	21.6	21.1	22.0
High	0 times/day	3	16.3	16.2	16.5	16.1	16.0	16.3
		5	15.8	15.7	15.9	15.8	15.7	15.9
		7	16.1	16.1	16.2	16.2	16.1	16.3
		9	17.1	17.0	17.2	17.1	17.0	17.2
		11	18.2	18.1	18.3	18.4	18.2	18.5
		13	19.2	19.0	19.4	19.6	19.4	19.8
		15	19.6	19.2	20.0	20.7	20.3	21.1
	2 times/day	3	16.3	16.1	16.4	16.1	16.0	16.2
		5	15.8	15.7	15.8	15.8	15.7	15.8
		7	16.2	16.1	16.2	16.2	16.1	16.3
		9	17.1	17.0	17.2	17.1	17.0	17.2
		11	18.2	18.0	18.3	18.3	18.2	18.5
		13	19.1	18.9	19.3	19.5	19.3	19.7
		15	19.3	19.0	19.7	20.5	20.1	20.8

Figure and Table: Marginal mean BMI values comparing children consuming fruits 0 vs 2 times/day stratified by sex and ISCED level.

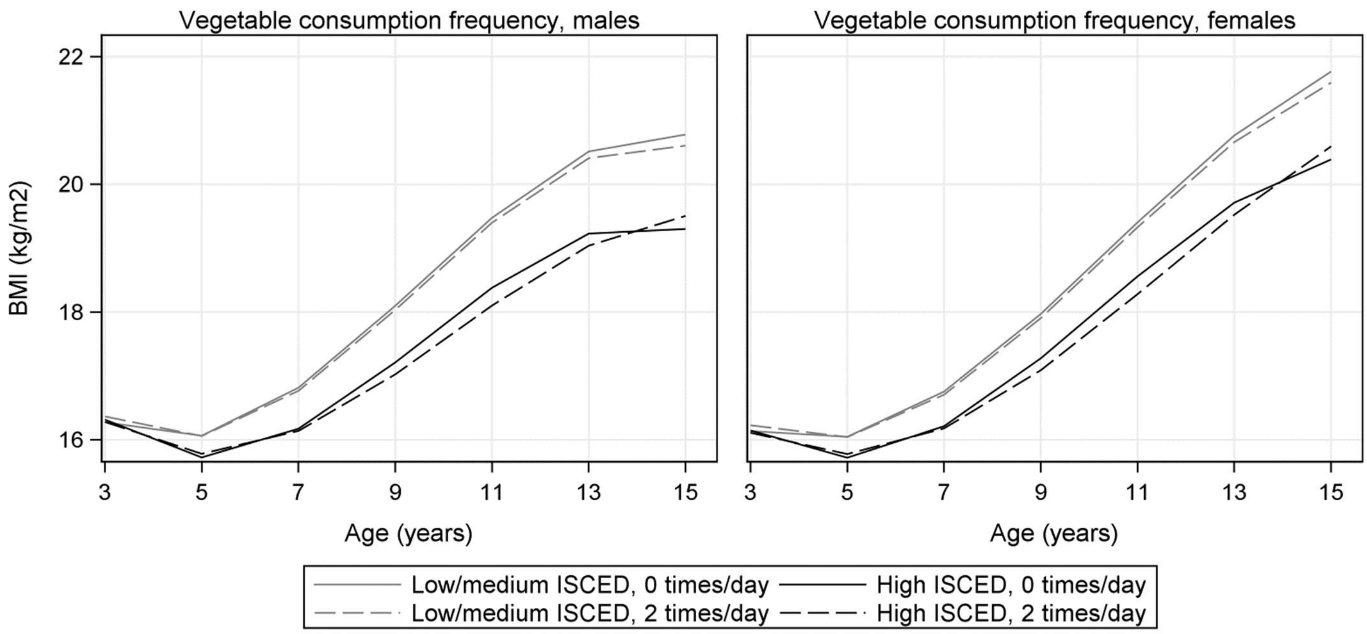

ISCED	Vegetable consumption	Age	Males Estimated BMI	Males LCL	Males UCL	Females Estimated BMI	Females LCL	Females UCL
Low/medium	0 times/day	3	16.3	16.1	16.5	16.1	15.9	16.4
		5	16.1	15.9	16.2	16.0	15.9	16.2
		7	16.8	16.7	16.9	16.8	16.6	16.9
		9	18.1	18.0	18.2	18.0	17.8	18.1
		11	19.5	19.3	19.6	19.4	19.2	19.6
		13	20.5	20.2	20.8	20.8	20.5	21.0
		15	20.7	20.3	21.2	21.8	21.2	22.3
	2 times/day	3	16.4	16.2	16.6	16.2	16.0	16.4
		5	16.1	16.0	16.2	16.0	15.9	16.2
		7	16.8	16.7	16.9	16.7	16.6	16.8
		9	18.0	17.9	18.2	17.9	17.8	18.0
		11	19.4	19.2	19.6	19.3	19.2	19.5
		13	20.4	20.2	20.6	20.7	20.4	20.9
		15	20.6	20.1	21.0	21.6	21.1	22.0
High	0 times/day	3	16.3	16.1	16.5	16.1	16.0	16.3
		5	15.7	15.6	15.8	15.7	15.6	15.8
		7	16.2	16.1	16.3	16.2	16.1	16.3
		9	17.2	17.1	17.3	17.3	17.2	17.4
		11	18.4	18.2	18.5	18.6	18.4	18.7
		13	19.2	19.0	19.4	19.7	19.5	19.9
		15	19.3	18.9	19.7	20.4	20.0	20.8
	2 times/day	3	16.3	16.1	16.4	16.1	16.0	16.2
		5	15.8	15.7	15.9	15.8	15.7	15.9
		7	16.1	16.1	16.2	16.2	16.1	16.3
		9	17.0	16.9	17.1	17.1	17.0	17.2
		11	18.1	18.0	18.2	18.3	18.2	18.4
		13	19.1	18.9	19.2	19.5	19.3	19.7
		15	19.5	19.2	19.9	20.6	20.3	21.0

Figure and Table: Marginal mean BMI values comparing children consuming vegetables 0 vs 2 times/day stratified by sex and ISCED level.

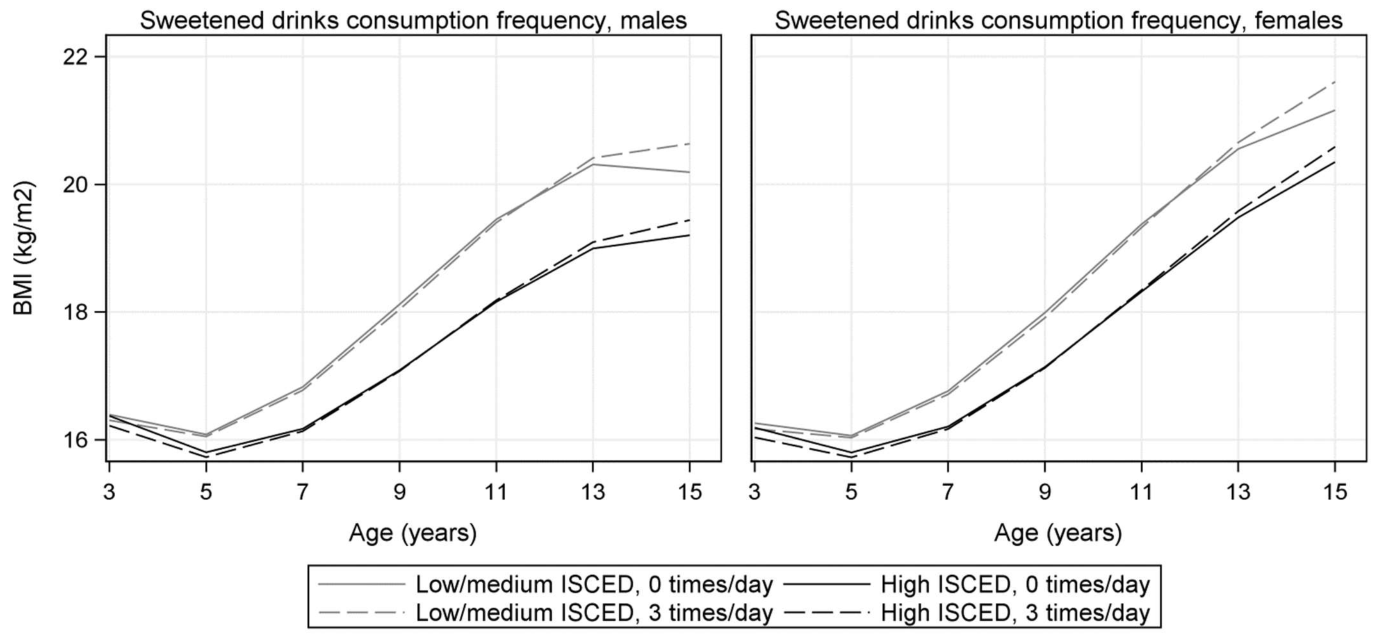

			Males			Females		
**ISCED**	**Sweetened drinks**	**Age**	**Estimated BMI**	**LCL**	**UCL**	**Estimated BMI**	**LCL**	**UCL**
Low/medium	0 times/day	3	16.4	16.2	16.6	16.3	16.0	16.5
		5	16.1	16.0	16.2	16.1	16.0	16.2
		7	16.8	16.7	16.9	16.8	16.6	16.9
		9	18.1	18.0	18.3	18.0	17.9	18.1
		11	19.4	19.3	19.6	19.4	19.2	19.5
		13	20.3	20.0	20.5	20.5	20.3	20.8
		15	20.2	19.7	20.6	21.1	20.7	21.6
	3 times/day	3	16.3	16.1	16.5	16.2	16.0	16.4
		5	16.1	15.9	16.2	16.0	15.9	16.1
		7	16.8	16.7	16.9	16.7	16.6	16.8
		9	18.0	17.9	18.2	17.9	17.8	18.0
		11	19.4	19.2	19.6	19.3	19.2	19.5
		13	20.4	20.2	20.6	20.6	20.4	20.9
		15	20.6	20.2	21.0	21.6	21.1	22.0
High	0 times/day	3	16.4	16.2	16.5	16.2	16.0	16.3
		5	15.8	15.7	15.9	15.8	15.7	15.9
		7	16.2	16.1	16.3	16.2	16.1	16.3
		9	17.1	17.0	17.2	17.1	17.0	17.2
		11	18.2	18.0	18.3	18.3	18.2	18.5
		13	19.0	18.8	19.2	19.5	19.3	19.7
		15	19.2	18.8	19.6	20.4	20.0	20.7
	3 times/day	3	16.2	16.1	16.4	16.0	15.9	16.2
		5	15.7	15.6	15.8	15.7	15.6	15.8
		7	16.1	16.0	16.2	16.2	16.1	16.3
		9	17.1	17.0	17.2	17.1	17.0	17.2
		11	18.2	18.1	18.3	18.3	18.2	18.5
		13	19.1	18.9	19.3	19.6	19.4	19.8
		15	19.5	19.1	19.8	20.6	20.2	21.0

Figure and Table: Marginal mean BMI values comparing children consuming sweetened drinks 0 vs 3 times/day stratified by sex and ISCED level.

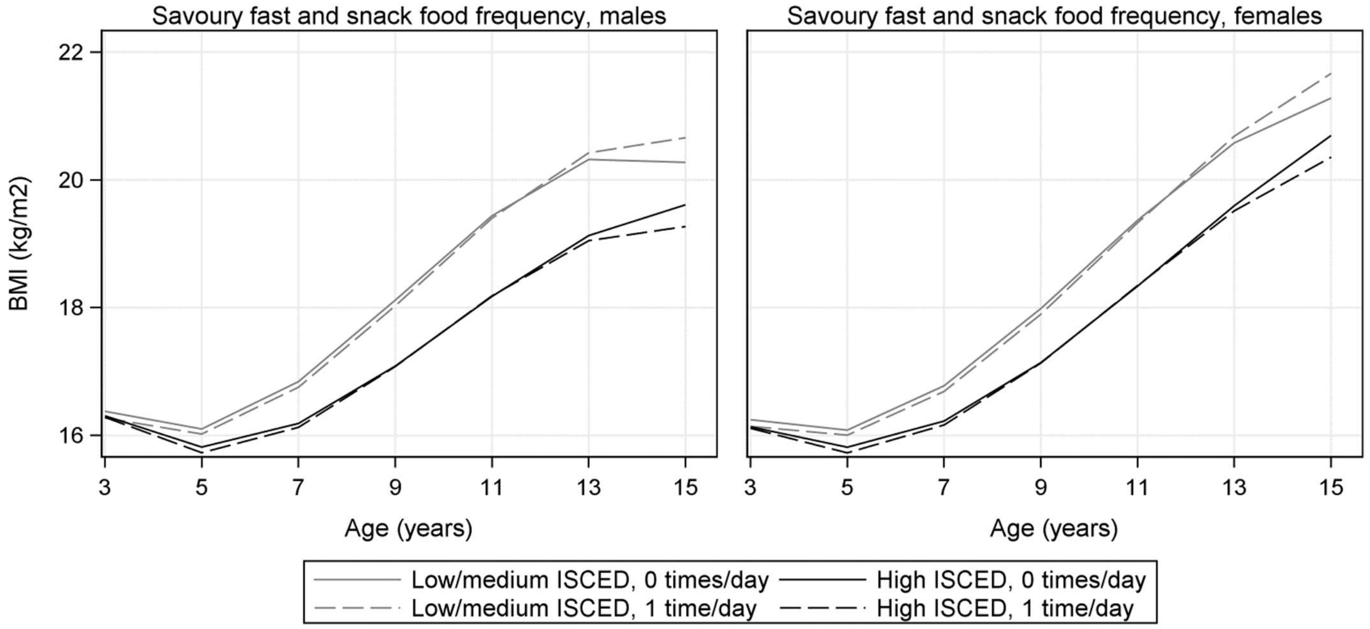

			Males			Females		
ISCED	Savoury fast/snack food	Age	Estimated BMI	LCL	UCL	Estimated BMI	LCL	UCL
Low/medium	0 times/day	3	16.4	16.2	16.6	16.2	16.0	16.5
		5	16.1	16.0	16.2	16.1	16.0	16.2
		7	16.8	16.7	17.0	16.8	16.7	16.9
		9	18.1	18.0	18.3	18.0	17.9	18.1
		11	19.4	19.3	19.6	19.4	19.2	19.5
		13	20.3	20.1	20.6	20.6	20.3	20.8
		15	20.3	19.8	20.7	21.3	20.8	21.8
	1 time/day	3	16.3	16.1	16.5	16.2	15.9	16.4
		5	16.0	15.9	16.1	16.0	15.9	16.1
		7	16.8	16.6	16.9	16.7	16.6	16.8
		9	18.0	17.9	18.2	17.9	17.8	18.0
		11	19.4	19.2	19.6	19.3	19.2	19.5
		13	20.4	20.2	20.7	20.7	20.4	20.9
		15	20.6	20.2	21.1	21.6	21.2	22.1
High	0 times/day	3	16.3	16.2	16.4	16.1	16.0	16.3
		5	15.8	15.7	15.9	15.8	15.7	15.9
		7	16.2	16.1	16.3	16.2	16.1	16.3
		9	17.1	17.0	17.2	17.1	17.0	17.2
		11	18.2	18.0	18.3	18.3	18.2	18.5
		13	19.1	18.9	19.3	19.6	19.4	19.8
		15	19.6	19.2	20.0	20.7	20.3	21.1
	1 time/day	3	16.3	16.1	16.4	16.1	15.9	16.3
		5	15.7	15.6	15.8	15.7	15.6	15.8
		7	16.1	16.0	16.2	16.2	16.1	16.2
		9	17.1	17.0	17.2	17.1	17.0	17.2
		11	18.2	18.1	18.3	18.3	18.2	18.5
		13	19.1	18.9	19.3	19.5	19.3	19.7
		15	19.3	18.9	19.7	20.4	20.0	20.7

Figure and Table: Marginal mean BMI values comparing children consuming savoury fast/snack foods 0 vs 1 times/day stratified by sex and ISCED level.

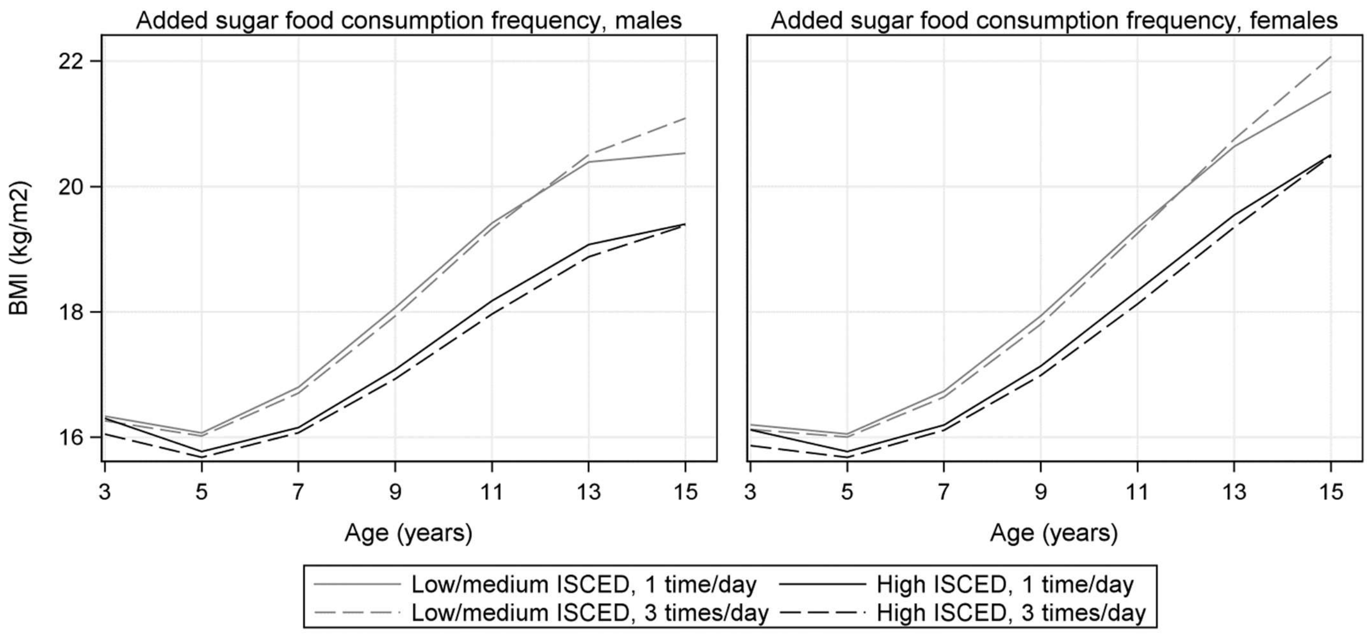

ISCED	Added sugar foods	Age	Males Estimated BMI	Males LCL	Males UCL	Females Estimated BMI	Females LCL	Females UCL
Low/medium	1 time/day	3	16.3	16.2	16.5	16.2	16.0	16.4
		5	16.1	16.0	16.2	16.1	16.0	16.2
		7	16.8	16.7	16.9	16.7	16.6	16.8
		9	18.1	17.9	18.2	17.9	17.8	18.1
		11	19.4	19.2	19.6	19.3	19.2	19.5
		13	20.4	20.1	20.6	20.6	20.4	20.9
		15	20.5	20.1	20.9	21.5	21.1	21.9
	3 times/day	3	16.3	16.0	16.5	16.1	15.9	16.4
		5	16.0	15.9	16.1	16.0	15.9	16.1
		7	16.7	16.6	16.8	16.6	16.5	16.8
		9	17.9	17.8	18.1	17.8	17.7	18.0
		11	19.3	19.1	19.5	19.2	19.1	19.4
		13	20.5	20.2	20.7	20.7	20.5	21.0
		15	21.0	20.5	21.5	22.0	21.5	22.5
High	1 time/day	3	16.3	16.2	16.4	16.1	16.0	16.2
		5	15.8	15.7	15.8	15.8	15.7	15.8
		7	16.2	16.1	16.2	16.2	16.1	16.3
		9	17.1	17.0	17.2	17.1	17.0	17.2
		11	18.2	18.1	18.3	18.3	18.2	18.5
		13	19.1	18.9	19.3	19.6	19.4	19.7
		15	19.4	19.1	19.8	20.5	20.2	20.9
	3 times/day	3	16.0	15.8	16.2	15.9	15.7	16.1
		5	15.7	15.6	15.8	15.7	15.6	15.8
		7	16.1	16.0	16.2	16.1	16.0	16.2
		9	16.9	16.8	17.1	17.0	16.9	17.1
		11	18.0	17.8	18.1	18.1	18.0	18.3
		13	18.9	18.7	19.2	19.4	19.1	19.6
		15	19.5	19.0	20.0	20.6	20.0	21.1

Figure and Table: Marginal mean BMI values comparing children consuming added sugar foods 1 vs 3 times/day stratified by sex and ISCED level.

References

1. United Nations Educational Scientific and Cultural Organization (UNESCO) (2012) International Standard Classification of Education, ISCED 2011. UNESCO Institute for Statistics, Canada
2. Bullinger M, Brutt AL, Erhart M et al. (2008) Psychometric properties of the KINDL-R questionnaire: results of the BELLA study Eur Child Adolesc Psychiatry 17 Suppl 1:125-132 doi:10.1007/s00787-008-1014-z
3. Ravens-Sieberer U, Bullinger M (2000) Kindl-R English questionnaire for measuring health-related quality of life in children and adolescents. Revised Version Manual. . Ulrike Ravens-Sieberer & Monika Bullinger,
4. Hense S, Barba G, Pohlabeln H et al. (2011) Factors that influence weekday sleep duration in European children Sleep 34:633-639 doi:10.1093/sleep/34.5.633
5. Santaliestra-Pasias AM, Mouratidou T, Verbestel V et al. (2014) Physical activity and sedentary behaviour in European children: the IDEFICS study Public health nutrition 17:2295-2306 doi:10.1017/s1368980013002486
6. Carskadon MA, Acebo C (1993) A self-administered rating scale for pubertal development J Adolesc Health 14:190-195
7. Huque MH, Moreno-Betancur M, Quartagno M et al. (2020) Multiple imputation methods for handling incomplete longitudinal and clustered data where the target analysis is a linear mixed effects model Biometrical Journal 62:444-466 doi:https://doi.org/10.1002/bimj.201900051
